# Holonomy of the Planar Brownian Motion in a Poisson Punctured Plane

**DOI:** 10.1007/s00220-024-05019-1

**Published:** 2024-05-27

**Authors:** Isao Sauzedde

**Affiliations:** Department of Statistics, University of Warwick, Coventry, UK

## Abstract

We define a family of diffeomorphism-invariant models of random connections on principal *G*-bundles over the plane, whose curvatures are concentrated on singular points. We study the limit when the number of points grows to infinity whilst the singular curvature on each point diminishes, and prove that the holonomy along a Brownian trajectory converges towards an explicit limit.

## Introduction

This paper is concerned with the way in which the Brownian motion winds around points in the plane. This question was studied in the past mostly from a homological point of view, that is by studying the winding of the Brownian motion around the points. One can mention in particular the celebrated result of Spitzer [26] about the Cauchy behaviour of the asymptotic winding around one point, when the Brownian motion runs up to a time that tends to infinity. It paved the way for an exact computation by Yor [29] of the distribution of this winding, at a fixed time, and also for further limits theorems when time tends to infinity [22]. Asymptotic winding around a family of points are also well known from the homological perspective (see e.g. [21]).

In [9, 10, 11, ...] (see also [13]), the effect of magnetic impurities on an electron in a 2 dimensional medium is studied by looking at the total winding (i.e. the sum of the winding) between this Brownian electron *X* and the Poisson distributed impurities. A particular attention is given to the limit when the number of impurities (i.e. the intensity of the Poisson process) goes to infinity. Following ideas of Werner on the set of points with given winding [27, 28], we showed in [24] that, when the intensity *K* of the Poisson process ${\mathcal {P}}$ of impurities goes to infinity, *X*-almost surely, the averaged winding converges in distribution (with respect to ${\mathcal {P}}$) towards a Cauchy variable centered at the Lévy area of *X*.

The model presented here corresponds to a more general interaction, associated with an arbitrary connected compact Lie group *G*, typically non-abelian. We consider a random flat *G*-bundle over $\mathbb {R}^2\setminus {\mathcal {P}}$, each point of ${\mathcal {P}}$ being associated with a monodromy element whose distance to 1 in *G* is proportional to $K^{-1}$; this correspond in the litterature to a Markovian holonomy field [16, 20], for which the underlying Lévy process is a Poisson one.

Our main theorem proves that the same kind of law arises at the limit as in the abelian case from [24]: *X*-almost surely, the holonomy along a Brownian curve *X* independent from ${\mathcal {P}}$ converges in distribution, as $K\rightarrow \infty$, towards the analogue in *G* of a Cauchy distribution, first introduced in [14].

Apart from the insight this gives in the structure of windings of planar Browian motion, we are motivated initially by relation with the Yang–Mills field (see e.g. [12, 17, 19] for construction and study of the Yang–Mills, or [7] for a more recent treatment built upon Parisi–Wu stochastic quantization and regularity structures). The 2-dimensional Yang–Mills field over the plane is a random map from a set of sufficiently smooth loops on $\mathbb {R}^2$ to a compact connected Lie group *G*. This map satisfies the algebraic properties of the holonomy function associated to a smooth connection on the trivial *G*-bundle over $\mathbb {R}^2$, but with a much lower regularity. The Yang–Mills field is therefore heuristically understood as the holonomy map of a very irregular random connection on this bundle. In Sobolev scale, the regularity of this random connection is expected to be $H^{-\varepsilon }$ (in a suitable gauge choice), which by far is too low to allow for a reasonable definition of its holonomy along a typical trajectories of the Brownian motion.

However, being able to define this holonomy along Brownian motion would have significant impact in quantum field theory, for it would allow to define probabilistically and compute the *k*-point functions of a Gaussian free field coupled with Yang–Mills field through Symanzik identities (see e.g. [18, Theorem 7.3] for a discrete analogous). The idea of replacing the Yang–Mills field with Markovian holonomy field can be found in [1].

Besides, Yang–Mills holonomy along Brownian path has already been looked at long ago, in the commutative case, by Albeverio and Kusuoka [2], with a radically different approach than ours. There renormalization procedure consists in cutting off the high frequency component of the Yang–Mills field, compute the Brownian holonomy in *G*, embedded as a matrix group so that one can average this holonomy, and then they rescale this average to get a non-trivial limit as they remove the cut-off.

In the particular case where the group *G* is abelian with Lie algebra ${\mathfrak {g}}$, the Yang–Mills field can be built from a ${\mathfrak {g}}$-valued white noise $\phi$ on $\mathbb {R}^2$. For a loop $\ell$ based at 0 and smooth enough, the Yang–Mills holonomy along $\ell$ is then simply given by $\phi (\theta _\ell )$, where $\theta _\ell$ is the winding function associated with $\ell$. $\phi (\theta _\ell )$ is well-defined for $\theta _\ell \in L^2(\mathbb {R}^2)$, and in particular for $\ell \in {\mathcal {C}}^1$.

Another way to define a map from a set of loops based on 0 to *G* in a multiplicative way is to take a Poisson process ${\mathcal {P}}$, and then to define a group homomorphism *h* from the fundamental group $\pi _1(\mathbb {R}^2\setminus {\mathcal {P}},0)$ to *G*. We will define a family of such random pairs $({\mathcal {P}},h)$, indexed by two parameters *K* (the intensity of the point process) and $\iota$ (the *curvature* or *charge* associated with each point, that is the distance between $1_G$ and the holonomy of a small loop winding once around one of these points) in such a way that their law is invariant under volume-preserving diffeomorphisms and gauge transformations. When $\iota$ is proportional to $K^{-\frac{1}{2}}$ and *K* tends to infinity, these random connections converge towards a Yang–Mills field, in the sense of finite-dimensional marginals. Our goal, however, is to study *another* scaling regime, when $\iota$ is proportional to *K* instead. Then, the holonomy of any given smooth loop converges in distribution towards $1_G$, but the holonomy along the Brownian curve (made into a loop by joining the endpoints smoothly) does not. Instead, it converges towards a 1-stable distribution in *G*.

## Definition of the Model and Presentation of the Main Result

Let *G* be a connected compact Lie group, of which we denote the unit element by 1. We endow its Lie algebra ${\mathfrak {g}}$ with a bi-invariant scalar product, and we denote by $\Vert \cdot \Vert$ the associated norm. This Euclidean structure on $\mathfrak g$ gives rise to a bi-invariant Riemannian structure on *G*, and we denote by $d_G$ the corresponding distance. For a positive real number *K*, the uniform probability distribution on the sphere of radius of $\frac{1}{K}$ of ${\mathfrak {g}}$ is denoted $\mu _K$, and we abbreviate $\mu _1$ into $\mu$.

Let ${\mathcal {P}}^{\mathfrak {g}}_K$ be a Poisson process on $\mathbb {R}^2\times {\mathfrak {g}}$, with intensity $K \lambda \otimes \mu _K$, where $\lambda$ is the Lebesgue measure on $\mathbb {R}^2$. Let also

$$\begin{aligned} {\mathcal {P}}^G_K:=\{(x,\exp (Z)): (x,Z)\in {\mathcal {P}}^{\mathfrak {g}}_K\} \end{aligned}$$

and ${\mathcal {P}}_K$ be the projection of ${\mathcal {P}}^G_K$ on $\mathbb {R}^2$. The projection $\pi :{\mathcal {P}}^G_K\rightarrow {\mathcal {P}}_K$ is almost surely injective, and we define $h_K:{\mathcal {P}}_K\rightarrow G$ the unique map such that $({{\,\textrm{Id}\,}}_{\mathbb {R}^2},h_K)=\pi ^{-1}$. It can be uniquely extended into a group homomorphism ${\bar{h}}_K$ from the free group ${\mathbb {F}}_{ {\mathcal {P}}_K}$ generated by ${\mathcal {P}}_K$ to *G*.

To define a group homomorphism from the fundamental group $\pi _1({\mathbb {R}}^2\setminus {\mathcal {P}},o)$ to *G*, it thus suffices to fix an isomorphism between ${\mathbb {F}}_{ {\mathcal {P}}_K}$ and $\pi _1({\mathbb {R}}^2{\setminus } {\mathcal {P}},o)$. This, however, cannot be done in a canonical way.

For any locally finite set ${\mathcal {P}}\subset \mathbb {R}^2$, we will define (Definition 3.6) a family ${{\,\textrm{Prop}\,}}_{\mathcal {P}} \subset {{\,\textrm{Iso}\,}}(\pi _1({\mathcal {P}}),{\mathbb {F}}_{ {\mathcal {P}}})$ of group isomorphisms from $\pi _1({\mathcal {P}}):=\pi _1(\mathbb {R}^2{\setminus } {\mathcal {P}},0)$ to ${\mathbb {F}}_{ {\mathcal {P}}}$ that we will call *proper*. This family as the following properties:

- It is uniquely defined from the data of the smooth oriented surface $\mathbb {R}^2$ and the subset ${\mathcal {P}}$. In particular, it is invariant by volume-preserving diffeomorphisms.
- If $(h_x)_{x\in {\mathcal {P}}}\in G^{\mathcal {P}}$ is a collection of independent random variables with conjugation invariant distributions, for any (deterministic) $\phi ,\psi \in {{\,\textrm{Prop}\,}}_{{\mathcal {P}}}$, the homomorphisms ${\bar{h}}\circ \phi$ and ${\bar{h}}\circ \psi$ are equal in distribution.

Then, for any family of deterministic loops $(\ell _i)_{i\in I}$ such that the $\lambda \Big (\bigcup _{i\in I} {{\,\textrm{Range}\,}}(\ell _i)\Big )=0$, a family of *G* valued random variables $(\omega _i)_{i\in I}$ is defined by $\omega _i={\bar{h}}\circ \phi ([\ell _i]_{\pi _1({\mathcal {P}}_K)})$. The distribution of this family does only depend on the specific choice of $\phi \in {{\,\textrm{Prop}\,}}_{{\mathcal {P}}_K}$, provided that it is independent from ${\mathcal {P}}^G_K$ conditional to ${\mathcal {P}}_K$.

Let also $X:[0,1]\rightarrow \mathbb {R}^2$ be a planar Brownian motion started from 0, independent from ${\mathcal {P}}^G_K$, and defined on a probability space $(\Omega ^X,{\mathcal {F}}^X,{\mathbb {P}}^X)$, and let ${\bar{X}}$ be its concatenation with the straight line segment between its endpoints. Then, ${\bar{X}}$ almost surely avoids ${\mathcal {P}}_K$, and we set $\omega _{\phi ,K}({\bar{X}})={\bar{h}}_K\circ \phi ([{\bar{X}}]_{\pi _1({\mathcal {P}}_K)})$.

The main result of this paper is the following.

### Theorem 1

Assume that the proper isomorphism $\phi$ between $\pi _1({\mathcal {P}}_K)$ and ${\mathbb {F}}_{ {\mathcal {P}}_K}$ is independent of $(X,{\mathcal {P}}^G_K)$ conditional on ${\mathcal {P}}_K$.

Then, ${\mathbb {P}}^X$-almost surely, as $K\rightarrow \infty$, $\omega _{\phi ,K}({\bar{X}})$ converges in distribution towards a 1-stable distribution on *G* that does not depend on $\phi$.

The limiting distribution $\nu ^*$ that we call 1-stable^1^ can be described as follows. Let *d* denote the dimension of *G* and

$$\begin{aligned} \sigma = \frac{\Gamma \big (\tfrac{d}{2})}{ 2 \sqrt{\pi } \Gamma \big (\tfrac{d+1}{2}\big )}. \end{aligned}$$

Let $\nu ^{\sigma }$ be the symmetric 1-stable probability distribution on ${\mathfrak {g}}$ with scale parameter $\sigma$, defined by its density with respect to the Lebesgue measure

1 $$\begin{aligned}{} & {} \frac{\;\textrm{d}\nu ^{\sigma }(Z)}{\;\textrm{d}Z}=\frac{1}{C_1}\frac{1}{\sigma ^d (1+\sigma ^{-2}\Vert Z\Vert ^2)^{\frac{d+1}{2}} }, \nonumber \\{} & {} C_1=\int _{{\mathfrak {g}}} \frac{1}{\sigma ^d (1+\sigma ^{-2}\Vert Z\Vert ^2)^{\frac{d+1}{2}} }\;\textrm{d}Z=\frac{\pi ^{\frac{d+1}{2}} }{\Gamma (\frac{d+1}{2}) }. \end{aligned}$$

There exists then a $\mathfrak g$-valued symmetric 1-stable process ${\tilde{Y}}$ such that ${\tilde{Y}}(1)$ is distributed according to $\nu ^{\sigma }$. This process has jumps, but we can ‘fill’ them with straight-line segments into a continuous curve $Y:[0,1]\rightarrow {\mathfrak {g}}$, defined up to increasing parametrization of [0, 1]. Such a parametrization can [5, Theorem 4.1] be chosen in such a way that *Y* has finite *p*-variation for $p>1$. In particular, taking $p<2$ allows to define the Cartan development *y* of *Y* on *G*. Then, $\nu ^*$ is equal to the distribution of *y*(1).

### Remark 2.1

The informed reader will have recognizd that the random group homomorphism ${\bar{h}}_K\circ \phi$ can be described as a Markovian holonomy field (see e.g. [16, 20] for the definition of Markovian holonomy fields).

To be more specific, let ${\mathscr {P}}({\mathbb {R}}^2)$ be a set of smooth enough paths in ${\mathbb {R}}^2$ (say, Lipschitz-continuous, although some other slightly different classes of paths can be used as well) and let $A:{\mathscr {P}}({\mathbb {R}}^2)\rightarrow G$ be a Markovian holonomy field, whose distribution is determined by its associated *G*-valued Lévy process *Z*, which jumps at rate *K* from $Z_{t-}$ to $\exp _G(V_t) Z_{t-}$, where $V_t$ is uniformly distributed on $\partial B(0, K^{-1})\subset {\mathfrak {g}}$, and stays constant in between these jumps. Then, it can be shown that there exists an underlying set ${\mathcal {P}}$ such that the restriction of *A* to loops which avoids ${\mathcal {P}}$ passes to the quotient into a group homomorphism from $\pi _1({\mathbb {R}}^2 )$ to *G*. This quotient is then equal in distribution to the group homomorphism ${\bar{h}}_K\circ \phi$ we will defined.

It is conceptually not difficult to see that these two objects agree in distribution, but it is technical in practice and is not the goal of this paper, which is very inspired indeed from the theory of Markovian holonomy fields (and in particular from [20]) but do not use it directly. Besides, the fact that the associated Lévy process is pure jump and only contains big jumps allow for much simplification compared to the general theory of Markovian holonomy fields. In fact, it is technically closer from random ramified coverings (see [20]) than from Markovian holonomy fields (although, since *G* isn’t discrete, it does not define a covering but a gauge-equivalence class of flat *G*-bundle instead). In particular, for *any* continuous loop, the holonomy along that loop is well defined with probability 1; whilst for general Markovian holonomy fields the loop need a bit of regularity (such as Lipschitz-continuity).

We thus provide an independent proof that the distribution of ${\bar{h}}_K\circ \phi$ does not depend on the specific choice of $\phi$. This would follow automatically from the equality in distribution we just mention. We would like to point however that this independent proof extends beyond the framework of Markovian holonomy field, since it extend to any locally finite point process, which need not be Poisson distributed (hence corresponding to a non-Markovian holonomy field).

### Strategy of the proof

In order to guide us through the proof, we first give an idea of why the main theorem holds, and of the main difficulty that arises, before giving a summary of the steps involved to solve it.

When a proper isomorphism has been fixed, the homotopy class $[\ell ]\in \pi _1({\mathcal {P}}_K)$ of a loop $\ell$ can be understood as a word on the letters $x,x^{-1}: x\in {\mathcal {P}}_K$. If we count algebraically each apparition of a letter *x* in $[\ell ]$, the relative integer that we obtain is equal to the winding number $\theta (x,\ell )$ of $\ell$ around the point *x*. If the order in which these letters appear were not important, the class $[\ell ]$ would thus be equal to

$$\begin{aligned}x_1^{\theta (x_1,\ell )}\dots x_n^{\theta (x_n,\ell )},\end{aligned}$$

where $\{x_1,\dots , x_n\}={\mathcal {P}}_K$. In Sect. 4, we will prove that Theorem  1 does hold if we replace $\omega _{\phi ,K}({\bar{X}})$ with

$$\begin{aligned}g_1^{\theta (x_1,\ell )}\dots g_n^{\theta (x_n,\ell )},\end{aligned}$$

where $\{(x_1,g_1),\dots , (x_n,g_n)\}={\mathcal {P}}_K^G$ is an ordering of ${\mathcal {P}}_K^G$, independent of ${\mathcal {P}}_K^G$ conditionally to ${\mathcal {P}}_K$. This will follow rather easily from the corresponding result with *G* replaced with ${\mathbb {R}}$, and indeed proves the theorem in the case the group *G* is abelian.

Let us now explain why it is reasonable to think that the order doesn’t matter indeed. First of all, with the kind of scaling that we have, and with the heavily tailed Cauchy distribution appearing, only the extremely large exponents (of the order of *K*) should contribute to the limit.

If a point appear once with an exponent of the order of *K*, it likely has a winding number which is at least of the order of *K*. The number of such points in ${\mathcal {P}}$ is only of the order of 1. Let us consider *x* one of them. Then, the Brownian motion *X* must go extremely close to *x*. Let us say that [*s*, *t*] is some small amount of time such that $X_s$ and $X_t$ are relatively far from *x*, but during which *X* goes very close to *x*. During that time, it might wind a large amount of time, say ${\hat{\theta }}(x)$, around *x*. Then, it is very unlikely that there is a *second* period of time during which *X* goes close to *x*, and since this is the only reasonable way for *X* to accumulate winding around *x*, we can deduce that $\theta (x)\simeq {\hat{\theta }}(x)$.

During the same period [*s*, *t*], the homotopy class of *X* will vary a lot, it will start from say *g* to $g h x^{{\hat{\theta }}(x)} h'$.^2^ Actually, it is possible to choose *s* and *t* so that additionally $h=h'=1$, provided that the proper isomorphism is chosen so that *x* corresponds to the path going straight ahead from 0 to *x*, winding once around *x*, and then going back straight to 0. If $x_1,\dots , x_k$ are these special points that appears once with a large exponent, we end up with a writing of $[{\bar{X}}]$ as

$$\begin{aligned} {[}{\bar{X}}]&=[X_{0,s_1}] x_1^{{\hat{\theta }}(x_1)} [X_{t_1,s_2}] x_2^{{\hat{\theta }}(x_2)} \dots [X_{t_k,1}]\\&= \big ([X_{0,s_1}]x_1^{{\hat{\theta }}(x_1)}[X_{0,s_1}]^{-1}\big ) \dots \\&\qquad \big ([X_{0,s_1}][X_{t_1,s_2}]\dots [X_{t_{k-1},s_{k}}] x_k^{{\hat{\theta }}(x_k)}[X_{t_{k-1},s_{k}}]^{-1}\dots [X_{0,s_1}]^{-1}\big ) \\&\qquad [X_{0,s_1}]\dots [X_{t_{k-1},s_{k}}] [X_{t_k,1}] \end{aligned}$$

where $X_{s,t}$ is the loop that starts from 0, goes straight to $X_s$, then follows *X* up to $X_t$, and then goes straight to 0. Since the loops $X_{t_i,s_{i+1}}$ do not have large winding around any point, it is expected that $\omega _{\phi ,K}([X_{0,s_1}]\dots [X_{t_k,1}])$ converges towards $1_G$ as $K\rightarrow \infty$. From the conjugation invariance of the variable associated with $x_1,\dots , x_k$, one can then expect that the distribution of $\omega _{\phi ,K}([{\bar{X}}])$ is very close from the one of $g_1^{{\hat{\theta }}(x_1)}\dots g_k^{{\hat{\theta }}(x_k)}$, which itself is very close from $g_1^{\theta (x_1)}\dots g_n^{\theta (x_n)}$.

One of the main problems we face when trying to make this rigorous is to actually get some control on $\omega _{\phi ,K}([X_{0,s_1}]\dots [X_{t_k,1}])$. The problem is that the length of the word corresponding to $[X_{0,s_1}]\dots [X_{t_k,1}]$ cannot be easily bounded, and we expect it to be way too large in general for a naive approach to be conclusive. What happens is that each time the Brownian motion does a ‘large winding’ around $x\in {\mathcal {P}}$ (that is a winding during which it is not specifically close from *x*), what will appear in $[X_{t_{i},s_{i+1}}]$ is not only $x^{\pm 1}$, but instead a conjugate $c x^{\pm 1}c^{-1}$, where *c* is a word whose length is of order *K* in general. Though the variables corresponding to *x* and $cxc^{-1}$ are identical in law, replacing one with the other would break the independence between the variables –as soon as *X* does a second turn around *x*, we get both $cxc^{-1}$ and $c'x{c'}^{-1}$ appearing in $[X_{0,s_1}]\dots [X_{t_k,1}]$. This independence is needed to apply some law of large numbers and show that $\omega _{\phi ,K}([X_{0,s_1}]\dots [X_{t_k,1}])$ converges to $1_G$. We solve this problem by finding a hierarchical structure between the different points that allows to simultaneously keep some independence and ignore the length of the conjugators *c*.

To be more specific, we will order the points in ${\mathcal {P}}=\{x_1,x_2,\dots \}$ and decompose the class $h=[{\bar{X}}]$ into a product

$$\begin{aligned} h=h_1\dots h_n, \end{aligned}$$

and decompose further each $h_i$ as

2 $$\begin{aligned} h_i=h_{i,1}\dots h_{i,j_i}\end{aligned}$$

in such a way that each $h_{i,j}$ is a conjugate of $x_i^{\pm 1}$, say $h_{i,j}=c_{i,j}x_i^{\pm _{i,j} 1} c_{i,j}^{-1}$, with only the letters $(x_k)_{k<i}$ allowed to appear in $c_{i,j}$. The goal of Sect. 5 is to show that such a decomposition is always possible, and to understand some of its aspects.

It is only on Sect. 9, with Proposition 8.3, that we will study the probabilistic interest of such a decomposition. To each $x_i$ will be associated a random element $X_i\in G$, very close from the unit, and we will study the product $X_h=X_{i_1}\dots X_{i_k}$, where $h=x_{i_1}\dots x_{i_k}$. Roughly speaking, we then manage to control the distance from $X_h$ to 1 in a way that depends only on the $j_i$ that appears in (2), and with a bound which is almost the same as when the group is commutative.

The *geometric* interest of this decomposition is studied in Sect. 6. Here, we make a specific choice for the order between the points of ${\mathcal {P}}_K$. We order them depending on their distance to the origin. This choice allows to show that for each point $x_i$, the number $j_i$ cannot be larger than some number $\theta _{\frac{1}{2}}(x_i)$ which, roughly speaking, counts some number of *half-turns* of *X* around $x_i$. The point is that this number $\theta _{\frac{1}{2}}(x_i)$ depends on *X* and on $x_i$, but not on the other points on ${\mathcal {P}}_K$.

In Sect. 7, we finally use the fact that our path is Brownian. We will see that the quantity $\theta _{\frac{1}{2}}(x_i)$ is then strongly related to the winding number $\theta (x_i)$, and that it is of order roughly $\theta (x_i)^2$. We will also consider the partition $\beta _{i,l}$ of $j_i$, defined by the indices *k* such that the conjugators $c_{i,k}$ and $c_{i,k+1}$ are different. We will give various upper bounds (with large probability) on the highest values $\beta _{i,1},\beta _{i,2},\beta _{i,3},\dots$ and on the possible size of set of indices *i* for which these values can be large.

In Sect. 8, we will arrange these bounds together to finally prove the main theorem.

Section 3 is devoted to prove that the distribution of $\omega _{\phi ,K}$ does not depend on $\phi$.

## Invariance by Volume-Preserving Diffeomorphism: From a Point Set to a Connection

In this section, we define the family ${{\,\textrm{Prop}\,}}_{\mathcal {P}}$ of proper group isomorphisms from $\pi _1({\mathcal {P}})$ to the group ${\mathbb {F}}_{{\mathcal {P}}}$ freely generated by ${\mathcal {P}}$. This family does not depend on any additional data, such as a Riemannian structure on the plane. We then show that the random connection $\omega _{\phi ,K}$ defined in the previous section does not depend, in distribution, from the isomorphism $\phi \in {{\,\textrm{Prop}\,}}_{\mathcal {P}}$. We will see that the braid groups acts freely and transitively on ${{\,\textrm{Prop}\,}}_{\mathcal {P}}$, so that we should first understand how braids act on the distribution of random variables.

### An easy lemma

Let $B_n$ be the Artin braid group with *n* strands,

$$\begin{aligned}{} & {} B_n=\langle b_1,\dots , b_{n-1}| \forall i\le n-2: b_ib_{i+1}b_i=b_{i+1}b_i b_{i+1}; \\ {}{} & {} \qquad \qquad \forall i,j\le n-1, |j-i|\ge 2: b_ib_j=b_jb_i \rangle .\end{aligned}$$

It admits a group homomorphism onto the symmetric group $S_n$, which maps the generator $b_i$ to the permutation $\sigma (b_i)=\sigma _i=(i,i+1)$. We denote by $\sigma$ this projection. It also admits a natural action on $G^n$, given by

$$\begin{aligned} b_i\cdot (g_1,\dots , g_n)= (g_1,\dots , g_{i-1}, g_{i+1}, g_{i+1}^{-1}g_i g_{i+1}, g_{i+2},\dots , g_n), \end{aligned}$$

as well as an action $\rho$ on ${\mathbb {F}}_n$, given by

$$\begin{aligned} \rho (b_i)(x_j)=\left\{ \begin{array}{ll} x_j &{} \hbox {if }j\notin \{i,i+1\},\\ x_{i}x_{i+1}x_i^{-1} &{}\hbox {if } j=i, \\ x_i &{}\hbox {if } j=i+1 \end{array} \right. , \quad \rho (b)(xy)=\rho (b)(x)\rho (b)(y). \end{aligned}$$

We propose here a simple lemma, which we do not use directly but which is somehow the key point to understand the distributional invariance of $\omega _{\phi ,K}$. This was first proved by [6, Lemma 6.12].

#### Lemma 3.1

Let $(X_1,\dots , X_n)$ be a family of independent *G*-valued random variables, each conjugation invariant. Then, the following equalities in distribution hold:

- for all $$i\in \{1,\dots , n-1\}$$,
  $$\begin{aligned} b_i\cdot (X_1,\dots , X_n)\overset{(d)}{=}(X_{\sigma _i(1)}, \dots , X_{\sigma _i(n)}). \end{aligned}$$
- More generally, for all $b\in B_n$,
  $$\begin{aligned} b\cdot (X_1,\dots , X_n)\overset{(d)}{=}(X_{\sigma (b)(1)}, \dots , X_{\sigma (b)(n)}). \end{aligned}$$

#### Proof

For the first item, we need to prove is that for all $i\in \{1,\dots , n-1\}$,

$$\begin{aligned}(X_1,\dots , X_{i-1},X_{i+1}, X_{i+1}^{-1}X_i X_{i+1}, X_{i+2},\dots , X_n )\overset{(d)}{=} (X_1,\dots X_{i-1}, X_{i+1},X_{i}, X_{i+2}, \dots X_n). \end{aligned}$$

From the independence assumption, this reduces to show that

$$\begin{aligned} (X_{i+1}, X_{i+1}^{-1}X_i X_{i+1})\overset{(d)}{=} (X_{i+1},X_i). \end{aligned}$$

For $j\in \{i,i+1\}$, let ${\mathbb {P}}^j$ be the law of $X_j$. Then, for all bounded ${\mathbb {P}}^i\otimes {\mathbb {P}}^{i+1}$-measurable function *f* from $G^2$ to $\mathbb {R}$,

$$\begin{aligned}&{\mathbb {E}}[f(X_i,X_{i+1} )]\\&\quad = \int _G \Big ( \int _G f(u,v) \;\textrm{d}{\mathbb {P}}^{i}_u \Big ) \;\textrm{d}{\mathbb {P}}^{i+1}_v\\&\quad = \int _G \Big ( \int _G f(v^{-1}uv,v) \;\textrm{d}{\mathbb {P}}^{i}_u \Big ) \;\textrm{d}{\mathbb {P}}^{i+1}_v \qquad \hbox {(from conjugation invariance of }{\mathbb {P}}^i\hbox {)}\\&\quad = {\mathbb {E}}[f(X_{i+1}^{-1}X_iX_{i+1}, X_{i+1})], \end{aligned}$$

which concludes the proof of the first item.

For a general $b\in B_n$, it suffices to write $b=b_{i_n}^{\varepsilon _n}\dots b_{i_1}^{\varepsilon _1}=b_{i_n}^{\varepsilon _n} b'$, with $\varepsilon _i\in \{\pm 1\}$ for all $i\in \{1,\dots , n\}$, and to proceed by recursion on *n*. It is easily seen that $(X_{\sigma (b')(1)}, \dots , X_{\sigma (b')(n)} )$ satisfies the same assumptions as $(X_1,\dots X_n)$.

We can thus apply the first item in the lemma to this family, and using also the recursion assumption we deduce that

$$\begin{aligned} b\cdot ( X_1,\dots , X_n)&\overset{(d)}{=} b_{i_n}^{\varepsilon _n} \cdot (X_{\sigma (b')(1)}, \dots , X_{\sigma (b')(n)} )\\&= (X_{\sigma (b_{i_n}^{\varepsilon _n})\circ \sigma (b')(1)}, \dots , X_{\sigma (b_{i_n}^{\varepsilon _n})\circ \sigma (b')(n)} )\\&=(X_{\sigma (b)(1)}, \dots , X_{\sigma (b)(n)} ), \end{aligned}$$

which concludes the recursion hence the proof. $\square$

### Finite set of punctures

The plane is endowed with its differential structure and orientation, and a point 0 is fixed. Let ${\mathcal {P}}$ be a finite subset of $\mathbb {R}^2 \setminus \{0\}$.

**Fig. 1:**
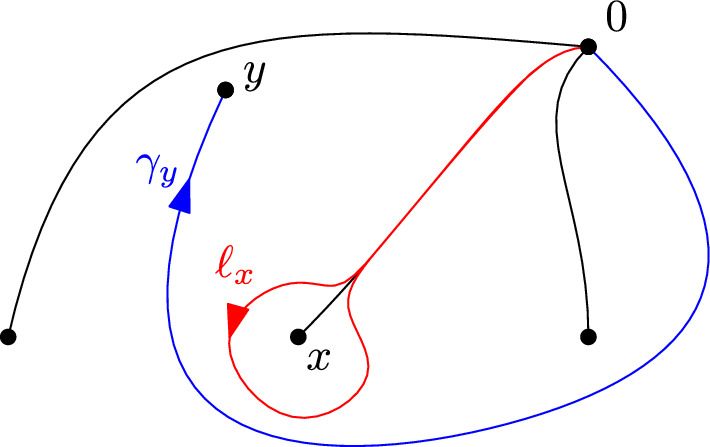
An example for $\# {\mathcal {P}}=4$

The fundamental group $\pi _1({\mathcal {P}})=\pi _1(\mathbb {R}^2\setminus {\mathcal {P}},0)$ is a free group with $\# {\mathcal {P}}$ generators. A basis can be obtained by first choosing $\# {\mathcal {P}}$ non-intersecting and non self-intersecting smooth paths $(\gamma _x)_{x\in {\mathcal {P}}}$, parameterized by [0, 1], with nowhere vanishing derivative $\dot{\gamma _x}$, and such that for all $x\in {\mathcal {P}}$, $\gamma _x$ is a path from 0 to *x*. Any such path $\gamma _x$ then determines a unique element $\ell _x$ in $\pi _1({\mathcal {P}} )$, a representative of which is given by a path that goes from 0 to a small neighbourhood of *x* following $\gamma _x$, then turns once around *x* in trigonometric order, and finally goes back to 0 following $\gamma$ backward. The family $(\ell _x)_{x\in {\mathcal {P}}}$ then freely generates $\pi _1({\mathcal {P}})$, and determines therefore a unique group isomorphism $\phi ((\ell _x)_{x\in {\mathcal {P}}})$ between $\pi _1({\mathcal {P}})$ and ${\mathbb {F}}_{{\mathcal {P}}}$. Figure 1 below represents a possible choice for the $\gamma _x$, and a path in one of the corresponding classes $\ell _x$. We define ${{\,\textrm{Prop}\,}}_{\mathcal {P}}$ as the set of isomorphisms obtained that way, which we call *proper*.

For a given map $h:{\mathcal {P}}\rightarrow G$, in order to understand how $\omega _{\phi ,h}:={\bar{h}}\circ \phi$ depends on $\phi$, one should first understand how proper isomorphisms are related one to the other. To this end, let us define ${{\,\textrm{Aut}\,}}$ the set of orientation-preserving homeomorphisms of $\mathbb {R}^2$ that maps 0 to itself and ${\mathcal {P}}$ to itself (possibly permuting the elements of ${\mathcal {P}}$). A function $f\in {{\,\textrm{Aut}\,}}$ induces a group automorphism $\rho (f)$ of $\pi _1({\mathcal {P}})$, given by $\rho (f)[\gamma ]=[f\circ \gamma ]$. It also induces a group automorphism $\sigma (f)$ of ${\mathbb {F}}_{\mathcal {P}}$, which maps $x\in {\mathcal {P}}$ to *f*(*x*). Finally, it defines an automorphism $\chi (f)$ of ${{\,\textrm{Prop}\,}}_{\mathcal {P}}$ given by

$$\begin{aligned} \chi (f)(\phi )=\sigma (f)\circ \phi \circ \rho (f)^{-1}.\end{aligned}$$

The three applications $\rho$, $\sigma$ and $\chi$ are then easily seen to be group actions of ${{\,\textrm{Aut}\,}}$.

#### Lemma 3.2

The action $\chi$ is transitive.

#### Proof

We fix a Euclidean structure on $\mathbb {R}^2$. Let $\phi , \psi$ be two proper isomorphisms, respectively obtained from the family of paths $(\gamma _x)_{x\in {\mathcal {P}}}$ and $(\delta _x)_{x\in {\mathcal {P}}}$. If there exists $f\in {{\,\textrm{Aut}\,}}$ such that for all $x\in {\mathcal {P}}$, $f\circ \gamma _x=\delta _{f(x)}$, then $\chi (f)(\psi )=\phi$. Thus, it suffices to show that such an homeomorphism *f* of $\mathbb {R}^2$ exists for all families $(\gamma _x)_{x\in {\mathcal {P}}}$ and $(\delta _x)_{x\in {\mathcal {P}}}$. We can assume that for all $x\in {\mathcal {P}}$, $\delta _x$ is the piecewise linear path from 0 to *x*.

**Fig. 2:**
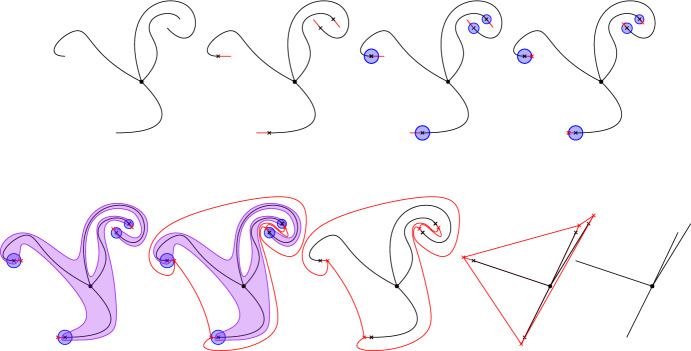
Black lines: the different paths $\gamma _x$. Red lines: the geodesic continuations $\gamma ^\varepsilon _x$. Blue circles: The neighbourhoods $U_x$. Red crosses: the points ${\tilde{x}}$. Purple set: the set $U_\infty$. Red curves: the curves ${\tilde{\gamma }}_x$. Eight picture: image of the previous one after application of an homeomorphism on each of the connected components

Figure 2 below pictures the different steps we use to build such an homeomorhism.

For each $x\in {\mathcal {P}}$, and $\varepsilon >0$, let $\gamma ^\varepsilon _x$ be the linear segment of length $\varepsilon$, starting from *x* and with direction $\dot{\gamma _x}(1)$. For $\varepsilon _0>0$ small enough, none of this path $\gamma ^\varepsilon _x$ cross any of the path $\gamma _y$ for $y\in {\mathcal {P}}$, nor any $\gamma ^{\varepsilon }_y$ for $y\ne x$. For $x\in {\mathcal {P}}$, let then $U_x$ be the ball of center *x* and radius $\varepsilon _1=\frac{\varepsilon _0}{2}$. In particular, these balls are disjoints. Let ${\tilde{x}}=x+ \varepsilon _1 \dot{\gamma _x}(1)$ be the point where $\gamma ^\varepsilon _x$ reaches the boundary of $U_x$.

Let then $\Gamma$ be a relatively compact, connected and simply connected neighbourhood of the union of the ranges of the $\gamma _x$, with a smooth boundary, small enough for all the ${\tilde{x}}$ to lie outside it. Set $U_\infty =\mathbb {R}^2{\setminus } (\Gamma \cup \bigcup _{x\in {\mathcal {P}}} U_x)$.

The boundary of $U_\infty$ is smooth by part, thus homeomorphic to a circle, which induces a cyclic order between the points ${\tilde{x}}$. Since $K_\infty$ is homeomorphic to $\mathbb {R}^2{\setminus } B(0,1)$, one can find for each *x* a continuous path ${\tilde{\gamma }}_x$ from ${\tilde{x}}$ to its successor $s({\tilde{x}})=\widetilde{s(x)}$, in such a way that these paths stay on $K_\infty$ and do not intersect each other.

Then, for each *x*, the concatenation of $\gamma _x$, $\gamma ^{\varepsilon _1}_x$, ${\tilde{\gamma }}_x$ and $\gamma _{s(x)}^{-1}$ is a continuous loop, and Jordan theorem ensures the existence of an homeomorphism from its interior to an open triangle. Applying this to each of these loop, and to the unbounded component delimited by the concatenation ${\tilde{\gamma }}_x {\tilde{\gamma }}_{s(x)} {\tilde{\gamma }}_{s^2(x)}\dots$, we obtain the desired homeomorphism. $\square$

#### Lemma 3.3

Let ${{\,\textrm{Aut}\,}}_0$ be the set of elements of ${{\,\textrm{Aut}\,}}$ isotopic to the identity. Then, the restriction of $\chi$ to ${{\,\textrm{Aut}\,}}_0$ is trivial. That is, for every $f\in {{\,\textrm{Aut}\,}}_0$ and $\phi \in {{\,\textrm{Prop}\,}}_{\mathcal {P}}$, $\chi (f)(\phi )=\phi$. Stated otherwise, $\chi$ passes to the quotient into a (transitive) action ${\tilde{\chi }}$ of the mapping class group $\pi _0({{\,\textrm{Aut}\,}})={{\,\textrm{Aut}\,}}/{{\,\textrm{Aut}\,}}_0$.

#### Proof

Let $f\in {{\,\textrm{Aut}\,}}_0$, and *h* be an isotopy from *f* to the identity. Then, for all $x\in {\mathcal {P}}$, $\gamma _x=(\gamma _{x,s})_{s\in S^1}$ is homotopic to $f(\gamma _x)$ by $(s,t)\mapsto h_t(\gamma _{x,s})$. Therefore, $[\gamma _x]=[f(\gamma _x)]$ and $\phi =\chi (f)(\phi )$. $\square$

This allows us to deduce the following result.

#### Lemma 3.4

Let ${\mathcal {P}}$ be a finite set and $(h_x)_{x\in {\mathcal {P}}}\in G^{\mathcal {P}}$ be a family of independent random variables, each having a distribution invariant by conjugation. Then, the law of $\omega _{\phi ,h}$ does not depend on $\phi \in {{\,\textrm{Prop}\,}}_{\mathcal {P}}$.

#### Proof

Since the mapping class group $\pi _0({{\,\textrm{Aut}\,}})$ acts transitively on ${{\,\textrm{Prop}\,}}$, it suffices to show that for one given proper isomorphism $\phi$ and $[f]\in \pi _0({{\,\textrm{Aut}\,}})$, $\omega _{\phi ,h}\overset{(d)}{=}\omega _{{\tilde{\chi }}(f)(\phi ),h}$. The mapping class group $\pi _0({{\,\textrm{Aut}\,}})$ is known (See e.g. [3, 4]) to be isomorphic to the Artin braid group group $B_n$. An isomorphism can be obtain as follow. Fix an identification between the plane and ${\mathbb {C}}$, such that the *n* points of ${\mathcal {P}}$ are identified with $1,\dots ,n$, and that the origin is located on $\{z: \Im (z)< -\frac{3}{4}$. For $j\in \{1,\dots , n-1\}$, we define the function $f_j\in {{\,\textrm{Aut}\,}}$ which maps $j+\frac{1}{2}+re^{i\theta }$ to $j+\frac{1}{2}+re^{i(\theta -\psi (r))}$, where

$$\begin{aligned}\psi (r)=\left\{ \begin{array}{ll} 0&{} \hbox { if } r\ge 1, \\ 4\pi (1-r)&{} \hbox {if } r\in \big [\tfrac{3}{4},1\big ],\\ \pi &{}\hbox {if } r\le \tfrac{3}{4}. \end{array}\right. \end{aligned}$$

**Fig. 3:**
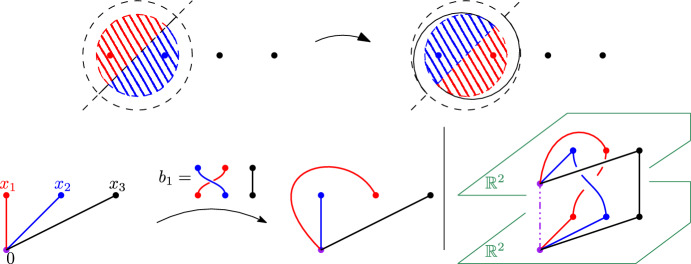
The map $f_1$, and the corresponding braid $b_{1}$ acting on a proper isomorphism. On the right-hand side, the colored loops can be extended into ‘sheets’ that do not cross each other but on the axe above 0

This function maps *x* to itself for $x\in \{1,\dots , n\}{\setminus } \{j,j+1\}$ and exchanges *j* with $j+1$ (see Fig. 3 below), so that $\sigma (f_j)=\sigma _j$. In fact, setting for $\gamma _x$ the straight line segment from the origin to *x* (and $\phi \in {{\,\textrm{Prop}\,}}_{\mathcal {P}}$ the element associated to this family of paths), we see that

$$\begin{aligned}{} & {} {[}f_j\circ \ell _x]= \left\{ \begin{array}{ll}[\ell _x]&{} \hbox {if } x\notin \{j,j+1\},\\ {[}\ell _j][\ell _{j+1}][\ell _{j}]^{-1} &{} \hbox {if } x=j,\\ {[}\ell _j]&{} \hbox {if } x=j+1, \end{array} \right. \\{} & {} {} [f^{-1}_j\circ \ell _x]= \left\{ \begin{array}{ll}[\ell _x] &{} \hbox {if }x\notin \{j,j+1\},\\ {[}\ell _{j+1} ] &{} \hbox {if } x=j, \\ {[}\ell _{j+1}]^{-1}[\ell _{j}][\ell _{j+1}] &{}\hbox {if } x=j+1. \end{array}\right. \end{aligned}$$

The family $([f_j])_{j\in \{1,\dots , n-1\}}$ generates $\pi _0({{\,\textrm{Aut}\,}})$, and the isomorphism $\iota$ betwen $\pi _0({{\,\textrm{Aut}\,}})$ and $B_n$ is obtained by mapping $[f_j]$ to the generator $b_j$.

In particular, since this family is generating, it suffices to show that for all $j\in \{1,\dots , n-1\}$, $\omega _{\phi ,h}\overset{(d)}{=}\omega _{{\tilde{\chi }}([f_j])(\phi ),h}$. Since both $\omega _{\phi ,h}$ and $\omega _{{\tilde{\chi }}([f_j])(\phi ),h}$ are group morphism and since $(\phi ^{-1}(i))_{i\in \{1,\dots , n\}}$ generates $\pi _1({\mathcal {P}})$, it suffices to show that

$$\begin{aligned} (\omega _{\phi ,h}(\phi ^{-1}(1)), \dots ,\omega _{\phi ,h}(\phi ^{-1}(n) ))\overset{(d)}{=}(\omega _{ {\tilde{\chi }}([f_j])(\phi ),h}(\phi ^{-1}(1)),\dots , \omega _{{\tilde{\chi }}([f_j])(\phi ),h}(\phi ^{-1}(n)) ). \end{aligned}$$

The left hand side is simply $(h_1,\dots , h_n)$. The right hand side is equal to

$$\begin{aligned}&({\bar{h}}\circ \sigma (f_j)\circ \phi [f_j^{-1}\circ \ell _1],\dots , {\bar{h}}\circ \sigma (f_j)\circ \phi [f_j^{-1}\circ \ell _n] )\\&\quad = (h_1,\dots , h_{j-1}, {\bar{h}}\circ \sigma (f_j)\circ \phi ([\ell _{j+1}] ) , \\&\qquad {\bar{h}}\circ \sigma (f_j)\circ \phi ( [\ell _{j+1}]^{-1}[\ell _{j}][\ell _{j+1}] ), h_{j+2},\dots , h_n )\\&\quad = (h_1,\dots , h_{j-1}, {\bar{h}}\circ \sigma _j\circ \phi ([\ell _{j+1}] ) ,\\&\qquad {\bar{h}}\circ \sigma _j\circ \phi ( [\ell _{j+1}]^{-1}[\ell _{j}][\ell _{j+1}] ), h_{j+2},\dots , h_n )\\&\quad = (h_1,\dots , h_{j-1}, {\bar{h}}\circ \sigma _j (x_{j+1}) ,{\bar{h}}\circ \sigma _j( x_{j+1}^{-1}x_j x_{j+1}^{-1}), h_{j+2},\dots , h_n )\\&\quad =(h_1,\dots , h_{j-1}, h_j , h_j^{-1}h_{j+1}h_j, h_{j+2},\dots , h_n ). \end{aligned}$$

We conclude as in Lemma 3.1, that is by using the fact that $(h_j,h_j^{-1}h_{j+1}h_j)\overset{(d)}{=} (h_j,h_{j+1})$. $\square$

#### Remark 3.5

If we assume that the variables $(\omega _x)_{x\in {\mathcal {P}}}$ are identically distributed, the proof can be slightly simplified by using the fact that both the action of $B_n$ at the source and the action of $S_n$ at the target preserve the distribution of $\omega _{\phi ,h}$.

We now extend Lemma 3.4 to the case when ${\mathcal {P}}$ is no longer finite but only locally finite.

### Locally finite set of punctures

#### Definition 3.6

Let ${\mathcal {P}}$ be a locally finite subset of $\mathbb {R}^2$. We say that a group morphism $\phi : \pi _1({\mathcal {P}})\rightarrow {\mathbb {F}}_{{\mathcal {P}}}$ is *proper* if there exists a family $(\gamma _x)_{x\in {\mathcal {P}}}$ which satisfies the following conditions.

- For all $x\in {\mathcal {P}}$, $\gamma _x:[0,1]\rightarrow \mathbb {R}^2$ is a path from 0 to *x*, with no self-intersection, a nowhere vanishing derivative, and such that $\phi ([\ell _x])=x\in {\mathbb {F}}_{\mathcal {P}}$, where $[\ell _x]$ is the previously defined homotopy element associated to $\gamma _x$.
- For all $$x,y\in {\mathcal {P}}$$, $$\gamma _x(s)=\gamma _y(t) \implies s=t=0 \hbox { or } x=y$$.
- The family $([\ell _x]_{x\in {\mathcal {P}}})$ generates $\pi _1({\mathcal {P}})$.

We set ${{\,\textrm{Prop}\,}}_{\mathcal {P}}$ the set of proper isomorphism.

Proper isomorphsims always exist: if no two points of ${\mathcal {P}}$ are colinear with 0, then we can take each $\gamma _x$ as the straight-line path from 0 to *x*. Otherwise, and since the notion of proper isomorphism does not rely on the specific euclidean structure of $\mathbb {R}^2$, it suffices to apply a diffeomorphism to return to the case with no two points of ${\mathcal {P}}$ colinear with 0.

#### Remark 3.7

**Fig. 4:**
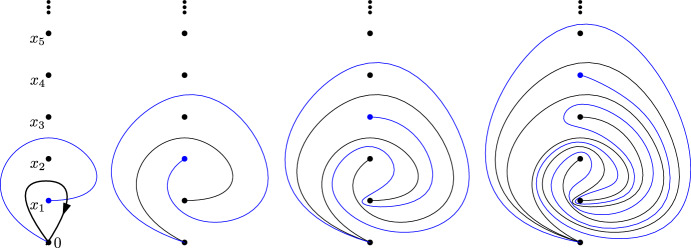
When ${\mathcal {P}}$ is no longer finite, the family $([\ell _x])_{x\in {\mathcal {P}}}$ can be free but not generating. In this example, the paths $\gamma _{x_i}$ are drawn one after the other. The path going to $x_{i+1}$ passes above the point $x_{i+2}$, and then navigates until it reaches $x_{i+1}$. The black loop which appears on the first picture does not lie on the group generated algebraically by the $[\ell _{x_i}]$. Formally, it would be described as $w [\ell _{x_1}] w^{-1}$ with *w* a bi-infinitely long word in the $[\ell _{x_i}]$

Contrary to the case when ${\mathcal {P}}$ is finite, the last condition is **not** superfluous. The subtlety is that the group generated by $([\ell _x])_{x\in {\mathcal {P}}}$ is a direct limit of groups, whilst $\pi _1({\mathcal {P}})$ is an inverse limit, in general larger. As a counter example, one can consider the case pictured in Fig. 4 below.

**Fig. 5:**
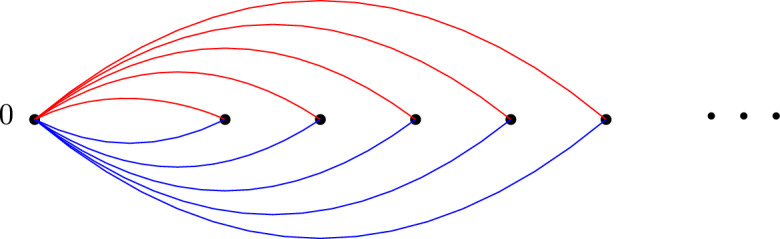
The two proper isomorphisms corresponding to the paths in red and blue are not related by the action of any braid

Besides, neither the direct limit of the finite braid groups nor $\pi _0({{\,\textrm{Aut}\,}}(\mathbb {R}^2\setminus {\mathcal {P}}))$ acts transitively on ${{\,\textrm{Prop}\,}}$ anymore. Figure 5 shows two proper isomorphisms which are not related by any such braids.

A proper basis ${\mathfrak {b}}=([\ell _x])_{x\in {\mathcal {P}}}$ induces an isomorphism between $\pi _1({\mathcal {P}})$ and the free group ${\mathbb {F}}_{ {\mathcal {P}} }$, and therefore allows to extend the definition of $\omega ^{{\mathfrak {b}}}$ to the case when ${\mathcal {P}}$ is locally finite.

#### Lemma 3.8

Let ${\mathcal {P}}$ be a locally finite set and $(h_x)_{x\in {\mathcal {P}}}\in G^{\mathcal {P}}$ be a family of independent random variables, each having a distribution invariant by conjugation. Then, the finite-dimensional distributions of $\omega _{\phi ,h}$ does not depend on $\phi \in {{\,\textrm{Prop}\,}}_{\mathcal {P}}$.

#### Proof

During this proof, for $\tilde{{\mathcal {P}}}\subseteq {\mathcal {P}}$, we write $[\ell _x]_{\tilde{{\mathcal {P}}}}$ the homotopy class of $\ell _x$ on $\mathbb {R}^2\setminus \tilde{{\mathcal {P}}}$.

Let $\phi ,\psi \in {{\,\textrm{Prop}\,}}_{{\mathcal {P}}}$ be two proper isomorphisms, associated respectively with $([\ell _x]_{\mathcal {P}})_{x\in {\mathcal {P}}}$ and $([\ell '_x]_{\mathcal {P}})_{x\in {\mathcal {P}}}$. Let $g^1,\dots , g^k\in \pi _1( {\mathcal {P}})$. Since $([\ell _x]_{\mathcal {P}})_{x\in {\mathcal {P}}}$ generates $\pi _1( {\mathcal {P}})$, there exists a finite set ${\mathcal {P}}'\subset {\mathcal {P}}$ such that for all $i\in \{1,\dots , k\}$, $g^i$ is spanned by $([\ell _x]_{\mathcal {P}})_{x\in {\mathcal {P}}'}$. Thus, to show that $(\omega _{\phi ,h}(g^i))_{i\in \{1,\dots , k\}}\overset{(d)}{=} (\omega _{\psi ,h}(g^i))_{i\in \{1,\dots , k\}}$, it suffices to show that for all finite set ${\mathcal {P}}'\subset {\mathcal {P}}$,

$$\begin{aligned}(\omega _{\phi ,h}([\ell _x]_{\mathcal {P}}))_{x\in {\mathcal {P}}'}\overset{(d)}{=} (\omega _{\psi ,h}([\ell _x]_{\mathcal {P}}))_{x\in {\mathcal {P}}'}.\end{aligned}$$

Since $([\ell '_x]_{\mathcal {P}})_{x\in {\mathcal {P}}}$ also generates $\pi _1( {\mathcal {P}})$, for all $x\in {\mathcal {P}}$, there exists $n_x\in {\mathbb {N}}$, $y_{x,1},\dots , y_{x,n_x}\in {\mathcal {P}}$ and $\varepsilon _{x,1},\dots , \varepsilon _{x,n_x}\in \{\pm 1\}$ such that

$$\begin{aligned} {[}\ell _x]_{{\mathcal {P}}}=[\ell '_{y_{x,1}}]_{{\mathcal {P}}}^{\varepsilon _{x,1}}\dots [\ell '_{y_{x,n_x}}]_{{\mathcal {P}}}^{\varepsilon _{x,n_x}}.\end{aligned}$$

Then,

$$\begin{aligned}\omega _{\psi ,h}([\ell _x]_{\mathcal {P}})=h_{y_{x,1}}^{\varepsilon _{x,1}}\dots h_{y_{x,n_x}}^{\varepsilon _{x,n_x}}.\end{aligned}$$

Let ${\mathcal {P}}''$ be the finite set $\{ y_{x,i}, x\in {\mathcal {P}}', i\in \{1,\dots , n_x\}\}$. For $x\in {\mathcal {P}}'$, the homotopy class of $\ell _x$ in $\pi _1({\mathcal {P}}'')$ is given by

$$\begin{aligned} {[}\ell _x]_{{\mathcal {P}}''}=\pi ( [\ell _x]_{{\mathcal {P}}})=\pi ([\ell '_{y_{x,1}}]_{{\mathcal {P}}}^{\varepsilon _{x,1}}\dots [\ell '_{y_{x,n_x}}]_{{\mathcal {P}}}^{\varepsilon _{x,n_x}} ) =[\ell '_{y_{x,1}}]_{{\mathcal {P}}''}^{\varepsilon _{x,1}}\dots [\ell '_{y_{x,n_x}}]_{{\mathcal {P}}''}^{\varepsilon _{x,n_x}},\end{aligned}$$

where $\pi$ is the canonical projection $[\ell ]_{{\mathcal {P}}}\mapsto [\ell ]_{{\mathcal {P}}''}$ from $\pi _1({\mathcal {P}})$ to $\pi _1({\mathcal {P}}'')$. It follows that

$$\begin{aligned} \omega _{\psi ,h_{|{\mathcal {P}}''}}( [\ell _x]_{{\mathcal {P}}''} )=h_{y_{x,1}}^{\varepsilon _{x,1}}\dots h_{y_{x,n_x}}^{\varepsilon _{x,n_x}}=\omega _{\psi ,h}([\ell _x]_{\mathcal {P}}).\end{aligned}$$

Since $\phi$, as an element of ${{\,\textrm{Prop}\,}}_{{\mathcal {P}}''}$, is associated with the family $([\ell _x]_{ {\mathcal {P}}''})_{x\in {\mathcal {P}}''}$,

$$\begin{aligned} \omega _{\phi ,h_{|{\mathcal {P}}''}}( [\ell _x]_{{\mathcal {P}}''} )=h_x=\omega _{\psi ,h}([\ell _x]_{\mathcal {P}}).\end{aligned}$$

Since ${\mathcal {P}}''$ is finite, one can apply Lemma 3.4 to the morphisms $\phi ,\psi \in {{\,\textrm{Prop}\,}}_{{\mathcal {P}}''}$, and deduce that

$$\begin{aligned} (\omega _{\phi ,h}( [\ell _x]_{{\mathcal {P}}} ))_{x\in {\mathcal {P}}''}= & {} (\omega _{\phi ,h_{|{\mathcal {P}}''}}( [\ell _x]_{{\mathcal {P}}''} ))_{x\in {\mathcal {P}}''} \overset{(d)}{=} (\omega _{\psi ,h_{|{\mathcal {P}}''}}( [\ell _x]_{{\mathcal {P}}''} ))_{x\in {\mathcal {P}}''} \\= & {} (\omega _{\psi ,h}( [\ell _x]_{{\mathcal {P}}} ))_{x\in {\mathcal {P}}''}, \end{aligned}$$

which concludes the proof. $\square$

#### Proposition 3.9

Assume that ${\mathcal {P}}_K^G$ is a Poisson process with intensity $K \lambda \otimes \nu$, where $\lambda$ is the Lebesgue measure on $\mathbb {R}^2$ and $\nu$ is a conjugation-invariant measure on *G*. Let ${\mathcal {P}}_K$ be the projection of ${\mathcal {P}}_K^G$ on $\mathbb {R}^2$, and $h_K: {\mathcal {P}}_K\rightarrow G$ be such that ${\mathcal {P}}_K^G=\{(x,h_K(x)):x\in {\mathcal {P}}_K\}$.

Then, the finite-dimensional distribution of $\omega _{\phi ,h_K}$ does not depend on the $\sigma ({\mathcal {P}}_K)$-measurable proper isomorphism $\phi$.

Besides, it is invariant by orientation and volume preserving diffeomorphisms, in the sense that for all deterministic orientation and volume preserving diffeomorphism $\psi$ of $\mathbb {R}^2$ with $\psi (0)=0$, and for all continuous oriented loops $\ell _1,\dots , \ell _k:[0,1] \rightarrow \mathbb {R}^2$ based at 0, and whose ranges has vanishing Lebesgue measure,

$$\begin{aligned} (\omega _{\phi ,h_K}[\ell _i]_{{\mathcal {P}}_K})_{i\in \{1,\dots , k\} }\overset{(d)}{=} (\omega _{\phi ,h_K}[\psi \circ \ell _i]_{{\mathcal {P}}_K})_{i\in \{1,\dots , k\} }. \end{aligned}$$

Remark that the vanishing range assumption is necessary for $\omega _{\phi ,h}[\ell _i]$ to be almost surely well-defined.

#### Proof

Let $\phi ,\psi$ be two $\sigma ({\mathcal {P}}_K)$-measurable elements of ${{\,\textrm{Prop}\,}}_{{\mathcal {P}}_K}$. Conditionally on $\sigma ({\mathcal {P}}_K)$, the variables $(h_K(x))_{x\in {\mathcal {P}}_K}$ are independent and each have a law $\nu$ invariant by conjugation. We can therefore apply Lemma 3.8, to deduce that conditionally on $\sigma ({\mathcal {P}}_K)$,

$$\begin{aligned}(\omega _{\phi ,h_K}[\ell _i])_{i\in \{1,\dots , k\} }\overset{(d)}{=} (\omega _{\phi ,h_K}[\psi \circ \ell _i])_{i\in \{1,\dots , k\} }.\end{aligned}$$

We conclude to the first assertion by integrating over $\sigma ({\mathcal {P}}_K)$.

To prove the second assertion, let $\psi ^G=\psi \times {{\,\textrm{id}\,}}_G$, $\tilde{{\mathcal {P}}}_K=\psi ({\mathcal {P}}_K)$, ${\tilde{h}}_K:{\tilde{P}}_K\rightarrow G$ the function which maps ${\tilde{x}}=\psi (x)$ to $h_K(x)$, $\tilde{{\mathcal {P}}}_K^G=\psi ^G({\mathcal {P}}_K^G)$, ${\bar{\psi }}:{\mathbb {F}}_{{\mathcal {P}}_K}\rightarrow {\mathbb {F}}_{\tilde{{\mathcal {P}}}_K}$ the unique group homomorphism that maps $x\in {\mathcal {P}}_K$ to $\psi (x)$, and ${\hat{\psi }}:\pi _1({\mathcal {P}}_K)\rightarrow \pi _1(\tilde{{\mathcal {P}}}_K )$ that maps $[\ell ]_{{\mathcal {P}}_K}$ to $[\psi \circ \ell ]_{\tilde{{\mathcal {P}}}_K}$ for all $\ell$. Let finally ${\tilde{\phi }}={\bar{\psi }}\circ \phi \circ {\hat{\psi }}^{-1}\in {{\,\textrm{Prop}\,}}_{\tilde{{\mathcal {P}}}}$. Then, we have the following commutative diagram.
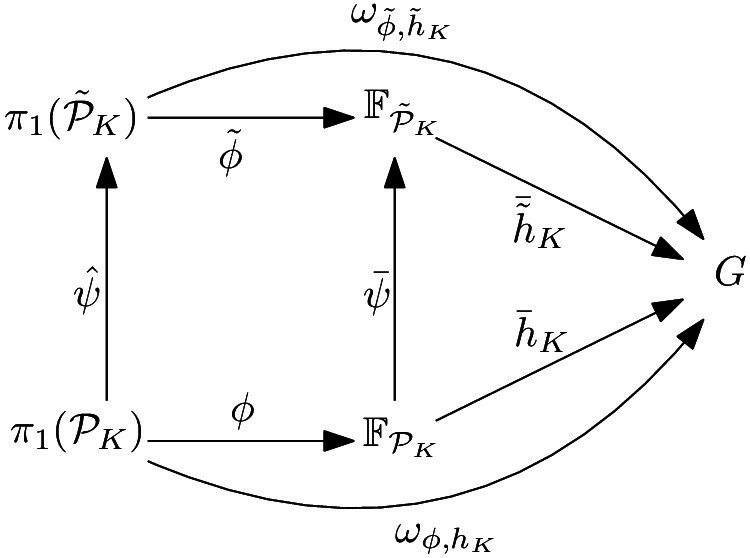


In particular, for all $\ell$, $\omega _{\phi ,h_K}([\ell ]_{{\mathcal {P}}_K})=\omega _{{\tilde{\phi }},{\tilde{h}}_K}\circ {\hat{\psi }}([\ell ]_{{\mathcal {P}}_K})=\omega _{{\tilde{\phi }},{\tilde{h}}_K}([\psi \circ \ell ]_{\tilde{{\mathcal {P}}}_K})$.

For ${\mathcal {P}}$ a locally finite set of $\mathbb {R}^2$, and $h\in {\mathcal {P}}^G$, let us denote ${\mathcal {P}}^G=\{(x,h(x)):x\in {\mathcal {P}}\}$, and $F_\phi ({\mathcal {P}}^G): \ell \mapsto \omega _{\phi ,h }([\ell ]_{{\mathcal {P}}})$.

The isomorphism $\psi ^G$ of $\mathbb {R}^2\times G$ preserves the measure $K\lambda \otimes \nu$, so that the sets ${\mathcal {P}}_K^G$ and $\tilde{{\mathcal {P}}}_K^G$ have the same distributions. We deduce that the finite-dimensional distributions of $F_\phi ({\mathcal {P}}^G_K)$ and $F_{{\tilde{\phi }}}(\tilde{{\mathcal {P}}}^G_K)$, which do not depend on $\phi$ nor on ${\tilde{\phi }}$, are equal. Therefore,

$$\begin{aligned} (\omega _{\phi ,h_K}[\ell _i]_{{\mathcal {P}}_K})_{i\in \{1,\dots , K\}}=(\omega _{{\tilde{\phi }},{\tilde{h}}_K}[\psi \circ \ell _i]_{\tilde{{\mathcal {P}}}_K})_{i\in \{1,\dots , K\}}\overset{(d)}{=}(\omega _{\phi ,h_K}[\psi \circ \ell _i]_{{{\mathcal {P}}}_K})_{i\in \{1,\dots , K\}}. \end{aligned}$$

$$\square $$

In the following, we assume that $\mathbb {R}^2$ is endowed with a Euclidean structure. For all *K*, almost surely, no pair of distinct points in ${\mathcal {P}}_K$ is colinear with 0, and a proper isomorphism $\phi _K$ is obtained by taking, for all $x\in {\mathcal {P}}$, as the path $\gamma _x$, the straight line segment from 0 to *x*. We set $\omega _K=\omega _{\phi _K,h_K}$. We implicitly identify $\pi _1({\mathcal {P}}_K)$ with ${\mathbb {F}}_{{\mathcal {P}}_K}$ using this isomorphism $\phi _K$.

## A much Simpler Problem

Let us recall that our goal is to study $\omega _K([{\bar{X}}])$, where ${\bar{X}}$ is the concatenation of the Brownian motion *X* with a straight line segment between its endpoints. This quantity can be written as a very large product of random elements in *G*,

$$\begin{aligned} \omega _K([{\bar{X}}])=g_{i_1}\dots g_{i_k}. \end{aligned}$$

If the group *G* was commutative, we could arrange this product as

3 $$\begin{aligned} g_1^{\theta (x_1,{\bar{X}})}\dots g_n^{\theta (x_n,{\bar{X}})}, \end{aligned}$$

where $(x_i,g_i)$ is an enumeration of our random set ${\mathcal {P}}^G_K$ and $\theta (x,{\bar{X}})$ is the integer winding number of ${\bar{X}}$ around *x*. Our goal, as we explained sooner, is actually to reduce our problem to the study of this simpler expression (3), even when *G* is not commutative. In this section, we show that the expression given by (3) does converge in distribution, as $K\rightarrow \infty$, and we identify the limit. Recall that the 1-stable law $\nu ^\sigma$ is defined by (1).

### Definition 4.1

We say that a probability measure $\nu$ on ${\mathfrak {g}}$ lies in the *strong attraction domain* of $\nu ^\sigma$, if $\nu$ is ${{\,\textrm{ad}\,}}$-invariant and there exists $\delta >0$ and a coupling $(X,Y): X\sim \nu ,\ Y\sim \nu ^\sigma$ such that ${\mathbb {E}}[|X-Y|^{1+\delta }]<+\infty$ and ${\mathbb {E}}[X-Y]=0$. We then write $\nu \in {\mathcal {D}}_\delta (\nu ^\sigma )$, and we write $[\nu ,\nu ^{\sigma }]$ for the law of such a coupling (*X*, *Y*).

### Proposition 4.2

Let *G* be a compact Lie group with Lie algebra ${\mathfrak {g}}$ endowed with a biinvariant scalar product. Let $\nu \in {\mathcal {D}}_\delta (\nu ^\sigma )$, for some $\sigma >0$.

Let $(X_i)$ be an i.i.d. sequence of random variables distributed according to $\nu$, and let $n_\lambda$ be an integer-valued random function of $\lambda$, independent from the sequence $(X_i)$, and such that $\frac{n_\lambda }{\lambda }$ converges in probability toward a deterministic $t>0$ as $\lambda \rightarrow \infty$. Then, as $\lambda \rightarrow \infty$, the random variable $\exp _G(\tfrac{X_1}{\lambda } )\dots \exp _G(\tfrac{X_{n_\lambda }}{\lambda })$ converges in distribution. The limit distribution is $\nu ^*=\nu ^*(t\sigma )$ defined below.

We now introduce very quickly the notion of geometric solution to ODE driven by càdlàg paths with finite *p*-variation, for $p<2$. This provides us with a rigorous definition for the distribution $\nu ^*$, and allow us to write a detailed version of Proposition 4.2 with intermediary steps to guide us through its proof.

For a ${\mathcal {C}}^1$ curve $\Gamma :[0,1]\rightarrow T_1G\cong {\mathfrak {g}}$ starting at 0, consider the ordinary differential equation

4 $$\begin{aligned} \gamma (0)=1 \qquad \frac{\;\textrm{d}}{\;\textrm{d}t}(\gamma (t_0)^{-1} \gamma (t) )_{|t=t_0}=\Gamma '(t). \end{aligned}$$

For $p<2$, Young integration theory teaches us that the solution map $I:\Gamma \mapsto \gamma$, extends continuously to the space ${\mathcal {C}}^{p-var}$ of continuous paths with finite *p*-variation (endowed with the *p*-variation distance). In fact, it can be extended further to the space ${\mathcal {D}}^{p-var}$ of càdlàg paths with finite *p*-variation variation distance, endowed with the Skororokhod-type *p*-variation metric $\alpha _p$ ([8, Definition 3.7, Proposition 3.10 *(ii)* and Proposition 3.18]. We still write *I* for the extended map, also known as the *Marcus* (or *geometric*) *canonical solution* to Eq. (4), and usually denoted

$$\begin{aligned} \;\textrm{d}\gamma (t)= \gamma (t) \diamond \;\textrm{d}\Gamma (t). \end{aligned}$$

When $\Gamma$ is a càdlàg semimartingale (in particular when $\Gamma$ is a Cauchy process), this also agree with the stochastic interpretation of the equation, defined in [14] prior to the notion of geometric canonical solution.

The distribution $\nu ^*(t\sigma )$ is then the distribution of $\gamma (t)$, when the driving process $\Gamma$ is a symmetric 1-stable process on ${\mathfrak {g}}$, whose distribution is uniquely determined by the property that $\Gamma (1)$ is distributed as $\nu ^\sigma$. Notice the definition of $\nu ^*(s)$ does not depend on the choice of *t* and $\sigma$ such that $s=t\sigma$, precisely because $\Gamma$ is 1-stable.

### Proposition

Let *G* be a compact Lie group with Lie algebra ${\mathfrak {g}}$ endowed with a biinvariant scalar product. Let ${\tilde{\nu }}^\sigma \in {\mathcal {D}}_\delta (\nu ^\sigma )$, for some $\sigma >0$.

Set $({\tilde{X}}_i,X_i)$ an i.i.d. sequence distributed according to $[{\tilde{\nu }}^\sigma ,\nu ^\sigma ]$. For $\lambda >0$, set $\Gamma ^{(\lambda )}$ and ${\tilde{\Gamma }}^{(\lambda )}$ be the continuous functions from $[0,+\infty )$ to ${\mathfrak {g}}$ given by

$$\begin{aligned} {\tilde{\Gamma }}^{(\lambda )}(t)&=\frac{1}{\lambda } \sum _{i=1}^{\lfloor \lambda t \rfloor } {\tilde{X}}_i+(\lambda t-\lfloor \lambda t\rfloor ) {\tilde{X}}_{\lfloor \lambda t\rfloor +1},\\ \Gamma ^{(\lambda )}(t)&=\frac{1}{\lambda } \sum _{i=1}^{\lfloor \lambda t \rfloor } X_i+(\lambda t-\lfloor \lambda t\rfloor ) X_{\lfloor \lambda t\rfloor +1}. \end{aligned}$$

Let also $\Gamma$ be a ${\mathfrak {g}}$-valued symmectric 1-stable process such that $\Gamma (1)$ is distributed as $\nu ^\sigma (1)$.

Then, the following hold.

1. For all $\lambda >0$ and all $i\le \frac{C}{\lambda }$, $I({\tilde{\Gamma }}^{(\lambda )})(\frac{i}{\lambda } )= \exp _G(\tfrac{{\tilde{X}}_1}{\lambda } )\dots \exp _G(\tfrac{{\tilde{X}}_i}{\lambda })$.
2. For all $p\in (1,1+\delta )$, and $C>0$, $\Vert d_{p-var,[0,C] }({\tilde{\Gamma }}^{(\lambda )}, \Gamma ^{\lambda } )\Vert _{L^1(\Omega )} \underset{\lambda \rightarrow \infty }{\longrightarrow }0$. In particular, $\alpha _{p-var,[0,C] }({\tilde{\Gamma }}^{(\lambda )}, \Gamma ^{\lambda })$ converges in distribution toward 0.
3. There exists a coupling of $\Gamma$ and $\Gamma ^{(\lambda )}$ such that $\Gamma ^{(\lambda )}$ is the piecewise-linear approximation of $\Gamma$ along the partition $(0,\lambda ^{-1}, 2\lambda ^{-1},\dots )$.
4. Let $p\in (1,2)$, $(\lambda _n)_{n\in {\mathbb {N}}}$ be a sequence of positive real number which diverges toward $+\infty$ as $n\rightarrow \infty$, and let all the $\Gamma ^{(\lambda _n)}$ be the piecewise-linear approximation of $\Gamma$, as above. Then, in probability, $\alpha _p(\Gamma ^{\lambda _n}, \Gamma ) \underset{n\rightarrow \infty }{\longrightarrow }0$.
5. For all $t>0$, the random variable $I({\tilde{\Gamma }}^\lambda )(t)$ converges in distribution, as $\lambda \rightarrow \infty$, toward $I(\Gamma )(t)$.
6. Let $n_\lambda$ be an integer-valued random function of $\lambda$, independent from the sequence ${\tilde{X}}_i$, and such that $\frac{n_\lambda }{\lambda }$ converges in probability toward a deterministic *t* as $\lambda \rightarrow \infty$. Then, as $\lambda \rightarrow \infty$, the random variable $\exp _G(\tfrac{X_1}{\lambda } )\dots \exp _G(\tfrac{X_{n_\lambda }}{\lambda })$ converges in distribution, and the limit law is the one of $I(\Gamma )(t)$.

### Proof

The first point follows from the fact that the family $( \frac{1}{\lambda } \sum _{i=1}^{j} X_i )_{j\in {\mathbb {N}} }$ is distributed as $(\Gamma (\tfrac{j}{\lambda }))_{j\in {\mathbb {N}}}$, and that when $x:[0, \lambda ^{-1} ]\rightarrow {\mathfrak {g}}$ is a straight-line segment from *a* to *b*, $I(x) (\lambda ^{-1})=\exp (b-a)$.

For the second item, we use the following interpolation inequality [15, Proposition 5.5]: for all $p\in (1,+\infty )$, for all continuous *f*,

5 $$\begin{aligned} \Vert f\Vert _{p-var,[0,C]}\le \Vert f\Vert _{1-var,[0,C]}^{\frac{1}{p}}\Vert f\Vert _0^{1-\frac{1}{p}} \end{aligned}$$

where $$\Vert f\Vert _0=\sup _{s,t\in [0,C]} |f(s)-f(t)|$$.

We also use the following inequality [23, Lemma 2]^3^.

### Lemma 4.3

Let $X_1,\dots , X_n$ be a family of independent and centered ${\mathbb {R}}$-valued random variables with moment of order $p<2$. Then,

6 $$\begin{aligned} {\mathbb {E}}\big [\big |\sum _{i=1}^n X_i\big |^p \big ]^{\frac{1}{p}}\le 2 \big (\sum _{i=1}^n {\mathbb {E}}[|X_i|^p ]\big )^{\frac{1}{p}}. \end{aligned}$$

We then obtain, for $p>1$:

$$\begin{aligned}&{\mathbb {E}}[ \Vert {\tilde{\Gamma }}^{\lambda }-\Gamma ^{\lambda } \Vert _{p-var,[0,C]} ]\\&\quad \le {\mathbb {E}}[ \Vert {\tilde{\Gamma }}^{\lambda }-\Gamma ^{\lambda }\Vert _{1-var,[0,C]}^{\frac{1}{p}} \Vert {\tilde{\Gamma }}^{\lambda }-\Gamma ^{\lambda }\Vert _{0}^{1-\frac{1}{p}} ] \qquad \hbox { (using (}5\hbox {))}\\&\quad \le {\mathbb {E}}[ \Vert {\tilde{\Gamma }}^{\lambda }-\Gamma ^{\lambda }\Vert _{1-var,[0,C]}]^{\frac{1}{p}} {\mathbb {E}}[\Vert {\tilde{\Gamma }}^{\lambda }-\Gamma ^{\lambda }\Vert _{0}]^{1-\frac{1}{p}} \qquad \hbox {(H}\ddot{\textrm{o}}\hbox {lder's inequality)} \\&\quad \le \big (\sum _{i=1}^{\lceil \lambda C \rceil } \tfrac{1}{\lambda }{\mathbb {E}}[|{\tilde{X}}_i-X_i|] \big )^{\frac{1}{p}} \big (2 {\mathbb {E}}[\Vert {\tilde{\Gamma }}^{\lambda }-\Gamma ^{\lambda }\Vert _{\infty }] \big )^{1-\frac{1}{p}} \qquad \hbox {(}\Vert f\Vert _0\le 2\Vert f\Vert _\infty \hbox {)}\\&\quad \le (\frac{\lceil \lambda C\rceil }{\lambda })^\frac{1}{p} {\mathbb {E}}[|{\tilde{X}}_1-X_1|]^{\frac{1}{p}} 2^{1-\frac{1}{p}} {\mathbb {E}}[ \max _{i\in \{0,\dots , \lceil C\lambda \rceil \}} \big | \sum _{j=1}^{i} \tfrac{1}{\lambda } ({\tilde{X}}_j-X_j) \big | ]^{1-\frac{1}{p}}. \end{aligned}$$

We now fix an arbitrary $q\in (1,1+\delta )$, and we apply Doob’s maximal inequality:

$$\begin{aligned} {\mathbb {E}}[ \Vert {\tilde{\Gamma }}^{\lambda }-\Gamma ^{\lambda } \Vert _{p-var} ]&\le C_{p,C} \lambda ^{-(1-\frac{1}{p})} \big ( \tfrac{q}{q-1} {\mathbb {E}}\big [\big |\sum _{j=1}^{\lceil C\lambda \rceil } ({\tilde{X}}_j-X_j)\big |^q \big ]^{\frac{1}{q}} \big )^{ 1-\frac{1}{p}} \\&\le C_{p,q,C}\lambda ^{-(1-\frac{1}{p})} \Big (2 \Big (\sum _{j=1}^{\lceil C\lambda \rceil } {\mathbb {E}}\big [ |{\tilde{X}}_j-X_j|^q \big ]\Big )^{\frac{1}{q}} \Big )^{ 1-\frac{1}{p}} \ \hbox {(using (}6\hbox {))}\\&\le C'_{p,q,C} \lambda ^{-(1-\frac{1}{p}) (1-\frac{1}{q}) } \underset{n\rightarrow +\infty }{\longrightarrow }0. \end{aligned}$$

This proves the second item.

For the third point, since ${\Gamma }^{(\lambda )}_t$ is piecewise-linear with interpolation points $( 0, \lambda ^{-1}, 2\lambda ^{-1},\dots )$, it suffices to show that

$$\begin{aligned} ({\Gamma }^{(\lambda )}_0, {\Gamma }^{(\lambda )}_{\lambda ^{-1}},\dots )\overset{(d)}{=}( \Gamma _0, {\Gamma }_{\lambda ^{-1}},\dots ), \end{aligned}$$

thus to show that the family of increments are identical in distributions. For both sides, the increments are i.i.d., and in both case distributed as $\nu ^\sigma$, which is enough to conclude.

The fourth item is a special case of Proposition 4.15 in [8].

For the fifth item, we use items 2 and 4, from which we deduce that ${\tilde{\Gamma }}^{(\lambda )}$ converges in distribution in $\alpha _p$-topology toward $\Gamma$. Take a probability space in which this convergence holds almost surely. Then, in the event the convergent occurs and *t* is a continuity point of $\Gamma$, we can use [8, Theorem 3.13], from which we deduce that $I({\tilde{\Gamma }}^{(\lambda )})(t)$ converges toward $I(\Gamma )(t)$. We conclude by noticing that *t* is almost surely a continuity point of $\Gamma$.

Finally for the final item, let $Z^\lambda =\exp _G( \frac{{\tilde{X}}_1}{\lambda } )\dots \exp _G( \frac{{\tilde{X}}_{n_\lambda } }{\lambda } )$. We focus on the event $E=\{ n_\lambda \ge \lambda t \}$, since the complementary event can be treated similarly, up to replacing the interval $[\lfloor \lambda t \rfloor , n_\lambda ]$ with $[n_\lambda ,\lfloor \lambda t \rfloor +1 ]$.

Under this event, the distance in *G* between $Z^\lambda$ and $I({\tilde{\Gamma }}^{\lambda })(t)$ is bounded by

$$\begin{aligned} \frac{1}{\lambda } \sum _{i\in {\mathbb {Z}}\cap [ \lfloor \lambda t \rfloor , n_\lambda ] } \Vert {\tilde{X}}_i\Vert\le & {} \frac{ n_\lambda -\lfloor \lambda t \rfloor +1 }{\lambda }\frac{1}{n_\lambda -\lfloor \lambda t \rfloor +1}\\{} & {} \Big ( \sum _{i \in {\mathbb {Z}}\cap [ \lfloor \lambda t \rfloor , n_\lambda ] } \Vert X_i\Vert + \sum _{i \in {\mathbb {Z}}\cap [ \lfloor \lambda t \rfloor , n_\lambda ] } \Vert {\tilde{X}}_i-X_i\Vert \Big ). \end{aligned}$$

We deduce that, on the event *E*,

$$\begin{aligned}&{\mathbb {P}}\big ( d_G(Z^\lambda ,I({\tilde{\Gamma }}^{\lambda })(t) )\ge \varepsilon \big |n_\lambda \big )\\&\quad \le \mathbb {1}_{ n_\lambda -\lfloor \lambda t \rfloor +1 \ge \varepsilon ^2 \lambda } + {\mathbb {P}}\big ( \frac{1 }{ n_\lambda -\lfloor \lambda t \rfloor +1 } \sum _{i \in {\mathbb {Z}}\cap [ \lfloor \lambda t \rfloor , n_\lambda ] } \Vert X_i\Vert \ge \frac{1}{2\varepsilon } \big |n_\lambda \big )\\&\qquad + {\mathbb {P}}\big ( \frac{1 }{ n_\lambda -\lfloor \lambda t \rfloor +1 } \sum _{i \in {\mathbb {Z}}\cap [ \lfloor \lambda t \rfloor , n_\lambda ] } \Vert {\tilde{X}}_i-X_i\Vert \ge \frac{1}{2\varepsilon } \big |n_\lambda \big )\\&\quad \le \mathbb {1}_{ n_\lambda -\lfloor \lambda t \rfloor +1 \ge \varepsilon ^2 \lambda } +( n_\lambda -\lfloor \lambda t \rfloor ) {\mathbb {P}}\big ( \Vert X_1\Vert \ge \frac{ n_\lambda -\lfloor \lambda t \rfloor +1 }{2\varepsilon } \big |n_\lambda \big )\\&\qquad +( n_\lambda -\lfloor \lambda t \rfloor ) {\mathbb {P}}\big ( \Vert {\tilde{X}}_1-X_1\Vert \ge \frac{ n_\lambda -\lfloor \lambda t \rfloor +1 }{2\varepsilon } \big |n_\lambda \big ). \end{aligned}$$

Since $X_1$ is 1-stable, there exists a constant *C* such that for all $z> 0$,

$$\begin{aligned} {\mathbb {P}}\big ( \Vert X_1\Vert \ge z \big ) \le \frac{C}{z}.\end{aligned}$$

Besides,

$$\begin{aligned} {\mathbb {P}}\big ( \Vert {\tilde{X}}_1 - X_1\Vert \ge z \big ) \le \frac{{\mathbb {E}}[ \Vert {\tilde{X}}_1-X_1\Vert ] }{z}.\end{aligned}$$

Thus, for $C'=\max (C,{\mathbb {E}}[ \Vert {\tilde{X}}_1-X_1\Vert ] )$, we get

$$\begin{aligned} {\mathbb {P}}\big ( d_G(Z^\lambda ,I({\tilde{\Gamma }}^{\lambda })(t) )\ge \varepsilon \big )\le {\mathbb {P}}( n_\lambda -\lfloor \lambda t \rfloor +1 \ge \varepsilon ^2 \lambda ) + 4\varepsilon C'\underset{\varepsilon \rightarrow 0}{\longrightarrow }0, \end{aligned}$$

which together with item 5 concludes the proof. $\square$

Before we explain how we will use Proposition 4.2, we need the following definition.

### Definition 4.4

For a point *x* outside the range of ${\bar{X}}$, the planar Brownian motion concatenated with a straight segment, we define $\theta (x)\in {\mathbb {Z}}$ the winding number of ${\bar{X}}$ around the point *x*.

### Theorem 4.5

Let ${\mathbb {P}}^X$ be the law of the Brownian motion (from [0, 1] to $\mathbb {R}^2$). For $R>0$, let *x* be a point distributed uniformly on *B*(0, *R*). Then, ${\mathbb {P}}^X$-almost surely on the event $\Vert X\Vert _\infty \le R$, the random variable $\theta (x)$ lies on the strong attraction domain of a Cauchy distribution with scale parameter $\frac{1}{2 \pi R^2}$.

### Proof

This is a direct corollary of Theorem 1.1 in our previous paper [24]. $\square$

### Lemma 4.6

Let *R* be a random variable on the strong attraction domain ${\mathcal {D}}_\delta (C)$ of a (real-valued) Cauchy distribution *C* with scale parameter *s*. Let *U* be a random variable independent from *R*, and distributed uniformly on the unit sphere of ${\mathfrak {g}}$. Then, *RU* lies on the attraction domain ${\mathcal {D}}_\delta (\nu ^\sigma )$ of the symmetric 1-stable distribution $\nu ^\sigma$ with $\sigma = \frac{ \Gamma \big ( \frac{d}{2}\big )}{\sqrt{\pi } \Gamma \big (\frac{d+1}{2} \big )}s$.

### Proof

One can and will assume that $s=1$. The general case is then recovered from a scaling argument.

Let *S* be a Cauchy random variable which is such that ${\mathbb {E}}[|R-S|^{1+\delta }]<+\infty$. Since ${\mathbb {E}}[\Vert RU-SU\Vert ^{1+\delta }]={\mathbb {E}}[|R-S|^{1+\delta }]<+\infty$, it suffices to show that there exists a random variable *Z* distributed as $\nu ^\sigma$ with ${\mathbb {E}}[\Vert SU-Z\Vert ^{1+\delta }]<+\infty$. Actually, we will show that there exists *Z* distributed as $\nu ^\sigma$ and such that $SU-Z$ is bounded.

For all $x\in [0,+\infty )$, let $\psi (x)$ be the unique positive real number solution of the equation

7 $$\begin{aligned} \frac{2}{\pi } \int _{\psi (x)}^{+\infty } \frac{\;\textrm{d}z}{1+z^2}=\frac{2 \Gamma \big (\tfrac{d+1}{2}\big ) }{\sqrt{\pi } \Gamma \big (\tfrac{d}{2}\big )} \int _{\sigma ^{-1} x}^{+\infty } \frac{z^{d-1} \;\textrm{d}z}{(1+z^2)^{\frac{d+1}{2} }}.\end{aligned}$$

The constants are tuned so that both sides are equal to 1 for $x=0=\psi (0)$. It is then easily seen that $\psi$ defines a continuous increasing bijection of $[0,+\infty )$. Set $\phi =\psi ^{-1}$. We set $Z_\phi ={{\,\textrm{sgn}\,}}(S)\phi (|S|)U$. We also set $Z_\sigma$ a random variable distributed as $\nu ^\sigma$.

Remark that $\Vert Z_\sigma \Vert$ and $\frac{Z_\sigma }{\Vert Z_\sigma \Vert }$ are independent variables, and that the latter is distributed uniformly over the unit sphere. The same is true for $Z_\sigma$ replaced with $Z_\phi$, and to show that $Z_\sigma \overset{(d)}{=} Z_\phi$ thus reduces to show that $\Vert Z_\sigma \Vert \overset{(d)}{=} \Vert Z_\phi \Vert$, which follows from a simple computation. Indeed,

$$\begin{aligned}{} & {} {\mathbb {P}}( \Vert Z_{\phi }\Vert \ge r )= {\mathbb {P}}( \phi (|S|) \ge r ) = 2{\mathbb {P}}(S\ge \psi (r)) = \frac{2}{\pi } \int _{\psi (r)}^{+\infty } \frac{\;\textrm{d}z}{1+z^2} =2\frac{\Gamma \big (\tfrac{d+1}{2}\big ) }{\sqrt{\pi } \Gamma \big (\tfrac{d}{2}\big )} \\{} & {} \quad \int _{\sigma ^{-1}r}^{+\infty } \frac{z^{d-1} \;\textrm{d}z}{(1+z^2)^{\frac{d+1}{2} }}. \end{aligned}$$

On the other hand,

$$\begin{aligned} {\mathbb {P}}( \Vert Z_{\sigma } \Vert \ge r )&= \int _{r}^{+\infty } |S^{d-1}| \frac{z^{d-1} \Gamma \big (\tfrac{d+1}{2}\big ) \;\textrm{d}z}{\pi ^{\frac{d+1}{2}}\sigma ^d (1+\sigma ^{-2} z^2)^{\tfrac{d+1}{2} } } =\frac{2\pi ^{\frac{d}{2}}\Gamma \big (\tfrac{d+1}{2}\big ) }{\pi ^{\frac{d+1}{2}} \Gamma \big (\tfrac{d}{2}\big )} \int _{\sigma ^{-1}r}^{+\infty } \frac{u^{d-1}\;\textrm{d}u}{(1+u^2)^{\frac{d+1}{2}} }\\&={\mathbb {P}}( \Vert Z_{\phi }U\Vert \ge r ). \end{aligned}$$

It follows that $Z_\phi$ is indeed distributed as $Z_\sigma$.

Let us now tune the parameter $\sigma$ so that ${{\,\textrm{sgn}\,}}(S)\phi (|S|)$ and *S* are close to each other.

Since

$$\begin{aligned}\int _{\psi (x)}^{+\infty } \frac{\;\textrm{d}z}{1+z^2}\underset{x\rightarrow \infty }{\sim }\frac{1}{\psi (x)} \qquad \hbox { and }\qquad \int _{\sigma ^{-1}x}^{+\infty } \frac{z^{d-1} \;\textrm{d}z}{(1+z^2)^{\frac{d+1}{2} }}\underset{x\rightarrow \infty }{\sim }\frac{1}{\sigma ^{-1}x}, \end{aligned}$$

Eq. (7) implies that $\frac{2}{\pi \psi (x)}\underset{x\rightarrow \infty }{\sim }\tfrac{2 \Gamma \big (\tfrac{d+1}{2} \big )}{\sqrt{\pi } \Gamma \big ( \frac{d}{2}\big ) \sigma ^{-1} x}$, or equivalently that $\psi (x)\underset{x\rightarrow \infty }{\sim }\tfrac{ \Gamma \big ( \frac{d}{2}\big ) x}{\sqrt{\pi } \Gamma \big (\tfrac{d+1}{2} \big )} \sigma ^{-1} x$, or equivalently that $\tfrac{\phi (x)}{x}\underset{x\rightarrow \infty }{\longrightarrow }\tfrac{\sqrt{\pi } \Gamma \big (\tfrac{d+1}{2} \big )}{ \Gamma \big ( \frac{d}{2}\big )} \sigma$.

For ${{\,\textrm{sgn}\,}}(S)\phi (|S|)$ and *S* to be close to each other, including when *S* is large, this limit must be one, so that we must set $\sigma :=\tfrac{ \Gamma \big ( \frac{d}{2}\big )}{\sqrt{\pi } \Gamma \big (\tfrac{d+1}{2} \big )}$.

A simple computation gives

$$\begin{aligned} \psi (x)\int _{\psi (x)}^{\infty } \frac{\;\textrm{d}z}{1+z^2}\underset{x\rightarrow +\infty }{=}1+O(x^{-2}), \quad \sigma ^{-1}x\int _{\sigma ^{-1} x}^{+\infty } \frac{z^{d-1}}{(1+z^2)^{\frac{d+1}{2} } }\;\textrm{d}z\underset{x\rightarrow +\infty }{=} 1+O(x^{-2} ). \end{aligned}$$

We deduce that

$$\begin{aligned} \psi (x)&=\Big (\int _{\psi (x)}^{\infty } \frac{\;\textrm{d}z}{1+z^2}\Big )^{-1}(1+O(x^{-2})) =\Big ( \sigma ^{-1} \int _{\sigma ^{-1} x}^{+\infty } \frac{z^{d-1}}{(1+z^2)^{\frac{d+1}{2} } }\;\textrm{d}z \Big )^{-1} (1+O(x^{-2}))\\&= \Big (\tfrac{1}{x}(1+O(x^{-2})) \Big )^{-1}(1+O(x^{-2}))=x+O(x^{-1}). \end{aligned}$$

It follows that $\psi (x)-x$ is bounded near $+\infty$, and by symmetry it is bounded on $\mathbb {R}$. Thus, ${{\,\textrm{sgn}\,}}(S)\phi (|S|) -S$ is bounded, and so is $Z_\phi -SU=({{\,\textrm{sgn}\,}}(S)\phi (|S|) -S) U$. $\square$

### Corollary 4.7

Let $\iota _K:{\mathcal {P}}_K\rightarrow {\mathbb {N}}$ be a (random) bijection, independent from $(X,{\mathcal {P}}_K^G)$ conditional to ${\mathcal {P}}_K$. Then, ${\mathbb {P}}_X$-almost surely, as $K\rightarrow \infty$, the product

$$\begin{aligned}\prod _{(g,x)\in {\mathcal {P}}_K^G\cap (B(0,\Vert X\Vert _\infty )\times G) } g^{\theta (x)},\end{aligned}$$

ordered according to $\iota _K$, converges in distribution towards $\nu ^{\sigma }$, for $\sigma =\frac{\Gamma \big (\tfrac{d}{2}\big )}{2\sqrt{\pi } \Gamma \big (\tfrac{d+1}{2}\big )}$.

### Proof

Let $x_1,\dots$ be the enumeration of ${\mathcal {P}}_K\cap B(0,\Vert X\Vert _\infty )$ such that $\iota _K(x_1)<\iota _K(x_2)<\dots$, and let $G_i\in {\mathfrak {g}}$ be the unique element such that $(x_i,G_i)\in {\mathcal {P}}_K^{\mathfrak {g}}$, and $g_i=\exp _G G_i$. Set $n_K:=|{\mathcal {P}}_K\cap B(0,\Vert X\Vert _\infty )|$, and let $\sigma _i$ be the elementary permutation of $\{1,\dots , n_K\}$ which switches the indices *i* and $i+1$. Conditionally on $(X,{\mathcal {P}}_K,\iota _K)$, the random variables $g_i^{\theta (x_i)}$ are conjugation invariant and globally independent. As we have seen already, it follows that conditionally on $(X,{\mathcal {P}}_K,\iota _K)$,

$$\begin{aligned}(g_i^{\theta (x_i)} )\overset{(d)}{=} ( g_1^{\theta (x_1)},\dots , g_{i-1}^{\theta (x_{i-1})}, g_{i+1}^{\theta (x_{i+1})} g_{i}^{\theta (x_{i})} g_{i+1}^{-\theta (x_{i+1})}, g_{i+1}^{\theta (x_{i+1})}, g_{i+2}^{\theta (x_{i+2})}, \dots ).\end{aligned}$$

In particular,

$$\begin{aligned} g_1^{\theta (x_1)}\dots g_{n_K}^{\theta (x_{n_k})} \overset{(d)}{=}g_1^{\theta (x_1)}\dots g_{i-1}^{\theta (x_{i-1})} g_{i+1}^{\theta (x_{i+1})} g_{i}^{\theta (x_{i})} g_{i+2}^{\theta (x_{i+2})} \dots g_{n_K}^{\theta (x_{n_k})}. \end{aligned}$$

Thus, for any permutation $\sigma$ which is independent from ${\mathcal {P}}^G_K$ conditionally on $(X,{\mathcal {P}}_K,\iota _K)$,

$$\begin{aligned} g_1^{\theta (x_1)}\dots g_{n_K}^{\theta (x_{n_k})} \overset{(d)}{=} g_{\sigma (1)}^{\theta (x_{\sigma (1)})}\dots g_{\sigma (n_K)}^{\theta (x_{\sigma (n_K)})}.\end{aligned}$$

We take $\sigma$ to be uniformly distributed among the permutations of $\{1,\dots , n_K\}$, and independent from $(X,{\mathcal {P}}^G_K,\iota _K)$ conditionally on $n_K$, and we set $y_i=x_{\sigma (i)}$, $H_i=G_{\sigma (i)}$, and $h_i=g_{\sigma (i)}$. Then, conditionally on $n_K$, the sequence $(y_i,H_i)_{i\in \{1,\dots , n_K\}}$ is independent from *X* and i.i.d. Furthermore each $(y_i,H_i)$ is composed of two independent variables, with $y_i$ distributed uniformly on $B(0,\Vert X\Vert _\infty )$ and $H_i$ distributed as $\nu ^\sigma$.

Notice this is in contrast with the initial sequence $(x_{i}, G_{i})_{i\in \{1,\dots , n_K\}}$, for which the choice of the bijection $\iota _K$ do not even ensure that $x_1$ is uniformly distributed on $B(0,\Vert X\Vert _\infty )$.

It then follows from Theorem 4.5 that ${\mathbb {P}}_X$-almost surely, for all *i*, conditionally on the event $i\le n_K$, $\theta _i:=\theta (y_i)$ lies in the strong attraction domain of a Cauchy distribution with scale parameter $\frac{1}{2\pi \Vert X\Vert _\infty ^2}$. By applying Lemma 4.6, we deduce that ${\mathbb {P}}_X$-almost surely, $\theta _i K H_i$ lies in the strong attraction domain of $\nu ^{{\tilde{\sigma }}}$, for ${\tilde{\sigma }}=\frac{\Gamma (\frac{d}{2})}{ \sqrt{\pi } \Gamma (\frac{d+1}{2}) 2\pi \Vert X\Vert _\infty ^2 }$.

By eventually extending the probability space on which ${\mathcal {P}}_K^G$ is defined, we can extend the sequence $(\theta _i, H_i)_{i\le n_K}$ into a sequence $(\theta _i,H_i)_{i\in {\mathbb {N}}}$ of i.i.d. random variables, globally independent from $n_K$ conditionally on $\Vert X\Vert _\infty$. Since $\frac{n_K}{K}$ almost surely converges toward $\pi \Vert X\Vert _\infty ^2$ as $K\rightarrow \infty$, we can apply Proposition 4.2. We deduce that ${\mathbb {P}}_X$ almost surely, conditionally on $n_K$, as $K\rightarrow \infty$, the *G*-valued random variable

$$\begin{aligned} h_1^{\theta _1}\dots h_n^{\theta _n} = \exp _G( \theta _1 H_1 )\dots \exp _G ( \theta _{n_K}H_{n_K} ) \end{aligned}$$

converges in distribution toward $\nu ^*=\nu ^*(\sigma )$, with

$$\begin{aligned} \sigma = \pi \Vert X\Vert _\infty ^2 {\tilde{\sigma }}= \frac{\Gamma (\frac{d}{2})}{ \sqrt{\pi } \Gamma (\frac{d+1}{2}) 2 }.\end{aligned}$$

This concludes the proof. $\square$

The problem that we now face, in order to prove the main theorem, is to replace this product with $\omega _K({\bar{X}})$.

## Free Groups

For two groups $K,K'$, we denote by $K* K'$ their free product. For a set *E*, we denote by ${\mathbb {F}}_E$ the free group freely generated by *E*.

### Free groups as semi-direct products

Let ${\mathcal {P}}=\{x_1,\dots ,x_k\}$ be a totally ordered finite set. We will build up a group isomorphism between the free group ${\mathbb {F}}_{{\mathcal {P}}}$ and a semi-direct product of free groups ${\mathbb {F}}_{{\mathcal {P}}{\setminus }\{x_k\}} \ltimes {\mathbb {F}}_{{\mathbb {F}}_{ {\mathcal {P}}{\setminus }\{x_k\} } }$.

Let *K* be a group and let $\pi :K* {\mathbb {Z}}\rightarrow K$ be the canonical projection. For simplicity, the canonical injections from *K* to ${\mathbb {F}}_K$ and from *K* to $K* {\mathbb {Z}}$ are not written explicitly. We write ${\textbf{1}}$ for the image of $1\in {\mathbb {Z}}$ into $K*{\mathbb {Z}}$ by the canonical injection. Here ${\textbf{1}}$ is a given generator of ${\mathbb {Z}}$, but we will use multiplicative notations otherwise (so for example ${\textbf{1}}^{k}$ is the usual $k\in {\mathbb {Z}}$). Notice also that ${\textbf{1}}$ is different from the unit element of $K*{\mathbb {Z}}$.

#### Lemma 5.1

Let *H* be the smallest normal subgroup of $K* {\mathbb {Z}}$ containing ${\textbf{1}}$. Then,

1. The kernel of $\pi$ is *H*.
2. The homomorphism $c: {\mathbb {F}}_K\rightarrow H\subseteq K* {\mathbb {Z}}$ which maps the generator $h\in K\subset {\mathbb {F}}_K$ to
  $$\begin{aligned} c(h)= h {\textbf{1}} h^{-1}\end{aligned}$$
  is an isomorphism.

Therefore,

$$\begin{aligned} 0\longrightarrow {\mathbb {F}}_K \overset{c}{\longrightarrow }\ K* {\mathbb {Z}} {\overset{\pi }{\rightleftarrows }} K\longrightarrow 0, \end{aligned}$$

is a split short exact sequence and $K* {\mathbb {Z}}\cong K \ltimes {\mathbb {F}}_{ K }$.

#### Proof

*First point:*

Since $\ker (\pi )$ is a normal subgroup and ${\textbf{1}} \in \ker (\pi )$, $H\subseteq \ker (\pi )$.

Let $g\in K* {\mathbb {Z}}$. Then, there exists $\varepsilon _1,\dots , \varepsilon _n\in \{\pm 1\}$ and $h'_1,\dots , h'_{n+1}\in K$ such that

$$\begin{aligned}g=h'_1{\textbf{1}}^{\varepsilon _1}h'_2\dots h'_n {\textbf{1}}^{\varepsilon _n} h'_{n+1}.\end{aligned}$$

Setting $h_i= h'_1\dots h'_i$, we get

$$\begin{aligned}g=(h_1{\textbf{1}}^{\varepsilon _1}h_1^{-1}) \dots (h_n {\textbf{1}}^{\varepsilon _n} h_n^{-1}) h_{n+1}.\end{aligned}$$

Then, $\pi (g)=\pi (h_{n+1})=h_{n+1}$. Therefore, $g\in \ker (\pi )$ implies $h_{n+1}=1_K$, which implies that $g\in \langle h {\textbf{1}}h^{-1}: h\in K \rangle _{K* {\mathbb {Z}} }=H$.

*Second point:* We only need to check that *c* is injective. Any $a\in {\mathbb {F}}_K\setminus \{1\}$ can be written as $a=h_1^{\alpha _1} \dots h_n^{\alpha _n}$ with $h_1\ne h_2\ne \dots \ne h_n\in K$ and $\alpha _1\dots \alpha _n\in {\mathbb {Z}}{\setminus }\{0\}$. Then *c*(*a*) is given by

$$\begin{aligned} h_1 {\textbf{1}}^\alpha _1 h_1^{-1}\dots h_n {\textbf{1}}^\alpha _n h_n^{-1}, \end{aligned}$$

which is a non-empty reduced word in $K* {\mathbb {Z}}$ since ${\textbf{1}}^\varepsilon _1$, $h_1^{-1} h_2$, ${\textbf{1}}^\varepsilon _2$, $h_2^{-1} h_3$, $\dots$, ${\textbf{1}}^\varepsilon _n$ are all different from 1 and lie alternatively in ${\mathbb {Z}}$ and *G*. Thus $c(a)\ne 1$, which concludes the proof. $\square$

#### Remark 5.2

We are very thankful to the anonymous reviewer who suggested to us this elementary proof of the second point, therefore drastically simplifying our initial proof, and who also noticed the proof works for a general group *K* rather than for the specific free groups *K* we will use it for.

Although we have not been able to find this lemma in the literature, we have little doubt this must be written somewhere; we apologize to the author we should have cited.

In the following, we write $\pi ^{k}$ for the canonical projection from ${\mathbb {F}}_{ {\mathcal {P}}}$ to ${\mathbb {F}}_{ {\mathcal {P}}{\setminus }\{x_k\}}$, and $c_k$ for the application from ${\mathbb {F}}_{{\mathbb {F}}_{ {\mathcal {P}}\setminus \{x_k\}}}$ to ${\mathbb {F}}_{ {\mathcal {P}}}\simeq {\mathbb {F}}_{ {\mathcal {P}}{\setminus }\{x_k\}} * {\mathbb {Z}}$ which maps $w\in {\mathbb {F}}_{ {\mathcal {P}}{\setminus }\{x_k\}}\subset {\mathbb {F}}_{{\mathbb {F}}_{ {\mathcal {P}}{\setminus }\{x_k\}}}$ to $wx_k w^{-1}$. When we want to explicitly the canonical inclusion of a set ${\mathcal {Q}}$ into the free group ${\mathbb {F}}_{{\mathcal {Q}}}$, we call it $\iota _{\mathcal {Q}}$.

By iteratively applying Lemma 5.1 (notice ${\mathbb {F}}_{ {\mathcal {P}}}\simeq {\mathbb {F}}_{ {\mathcal {P}}{\setminus }\{x_k\}} * {\mathbb {Z}} )$), we obtain an isomorphism

$$\begin{aligned} {\mathbb {F}}_{ {\mathcal {P}} }\cong \big ( \dots \big ( {\mathbb {F}}_{{\mathbb {F}}_{\emptyset }} \ltimes {\mathbb {F}}_{{\mathbb {F}}_{\{x_1\}}}\big )\ltimes {\mathbb {F}}_{{\mathbb {F}}_{\{x_1,x_2\}}}) \big )\ltimes \dots \big )\ltimes {\mathbb {F}}_{{\mathbb {F}}_{ {\mathcal {P}}-\{x_k\} }} \big ), \end{aligned}$$

where ${\mathbb {F}}_{\emptyset }$ is the group with a single element. We write $\phi ^j(g)$ the element on the component ${\mathbb {F}}_{{\mathbb {F}}_{ \{x_1,\dots , x_{j-1} \}}}$, so that

$$\begin{aligned}g=c^1 \circ \phi ^1(g)\dots c^k \circ \phi ^k(g).\end{aligned}$$

To be more down-to-earth, the only thing we are doing here is that we write $g\in {\mathbb {F}}_{ {\mathcal {P}}}$ as

$$\begin{aligned}g=g' (w_1x_k^{\varepsilon _1}w_1^{-1})\dots (w_nx_k^{\varepsilon _n}w_n^{-1}), \quad \hbox {with} \quad g',w_1,\dots , w_n \in {\mathbb {F}}_{ {\mathcal {P}}\setminus \{x_k\} },\end{aligned}$$

and we then iterate this procedure to further decompose $g'$ in a similar manner (but with $x_k$ replaced with $x_{k-1}$). As an example, the corresponding writing of $g=x_3x_2x_1x_4x_2x_4^{-1}$ isand more generally *g* is decomposed as a power of $x_1$, followed by a product of conjugates of $x_2$ (where the.“conjugator” only uses the letter $x_1$), followed by a product of conjugates of $x_3$ (where the conjugator only uses the letters $x_1$,$x_2$), and so on.

### Relevance of this decomposition

For a finite totally ordered set ${\mathcal {P}}=\{x_1,\dots , x_n\}$, we identify $x_j$ with *j*. For $g\in {\mathbb {F}}_{ {\mathcal {P}}}$ and $x_j\in {\mathcal {P}}$, we define

$$\begin{aligned} \pi ^{<j}:{\mathbb {F}}_{ {\mathcal {P}}}\rightarrow {\mathbb {F}}_{ \{1,\dots , {j-1} \}} \quad \hbox {and} \quad \pi ^{\le j}:{\mathbb {F}}_{ {\mathcal {P}}}\rightarrow {\mathbb {F}}_{ \{1,\dots , {j} \}} \end{aligned}$$

the canonical projections. In particular, we easily see that for all *j*,

$$\begin{aligned} \pi ^{\le j}(g)=c^1\circ \phi ^1(g)\dots c^j \circ \phi ^j(g),\end{aligned}$$

hence

8 $$\begin{aligned} c^j\circ \phi ^j(g)={\pi ^{<j}(g)}^{-1} \pi ^{\le j}(g). \end{aligned}$$

For a set ${\mathcal {Q}}$ which is either ${\mathcal {P}}$ or ${\mathbb {F}}_{\{1,\dots , k-1\} }$, and $g\in {\mathbb {F}}_{ {\mathcal {Q}}}$, we define an integer $\ell (g)$ and a finite sequence

$$\begin{aligned}(a_i(g), \alpha _i(g) )_{i\in \{1,\dots , \ell (g) \}} \end{aligned}$$

as the unique finite sequence with values in ${\mathcal {Q}}\times ({\mathbb {Z}}{\setminus }\{0\} )$, such that

$$\begin{aligned}g= \underbrace{a_1(g)\dots a_1(g)}_{\alpha _1(g) }\dots \dots \dots \underbrace{a_{\ell (g)}(g)\dots a_{\ell (g)}(g)}_{\alpha _{\ell (g)}(g) }=\prod _{i=1}^{\ell (g)} a_i(g)^{\alpha _i(g)},\end{aligned}$$

and such that $a_i(g)\ne a_{i+1}(g)$ for all $i\in \{1,\dots , \ell (g)-1\}$. For $i>\ell (g)$, we also set $\alpha _i(g)=0$.

For $x\in {\mathcal {Q}}$ and $g\in {\mathbb {F}}_{{\mathcal {Q}}}$, let $I^{x}(g)$ be the set of the indices *i* such that $a_i(g)=x$:

$$\begin{aligned} I^{x}(g)=\{i\in \{1,\dots , \ell (g)\}, a_i(g)=x\}. \end{aligned}$$

For $k\in \{1,\dots ,|I^{x}(g)|\}$, we set $i^x_{k}(g)$ the $k^{\hbox {th}}$ element of $I^{x}(g)$, so that

$$\begin{aligned} I^{x}(g)=\{ i^x_{1}(g),\dots , i^x_{|I_{x}(g)|}(g) \}, \quad i^x_{1}(g)<\dots < i^x_{|I^{x}(g)|}(g).\end{aligned}$$

We also set $$\alpha ^x_{k}(g)=\alpha _{i^x_{k}(g)}(g)$$, and for $$k > |I^{x}(g)| $$ we set $$\alpha ^x_{k}(g)=0$$. This gives us an ultimately vanishing sequence $$\alpha ^{x}(g)=(\alpha ^x_{k}(g) )_{k\in {\mathbb {N}}}$$, the sequence of the exponents to which *x* appears in *g*. As an example, for $$g=x^7y^{5}x^{-4}$$, $$I^{x}(g)=\{1,3\}$$, $$i^x_{1}=1$$, $$i^x_{2}=3$$, $$\alpha ^x_{1}=7$$, and $$\alpha ^x_{2}=-4$$.

We endow the set of integer valued and ultimately vanishing sequences with the order $\preccurlyeq$ obtain as the reflexive and transitive closure of the relation ${\mathcal {R}}$ given by

$$\begin{aligned} \alpha \ {\mathcal {R}} \ \alpha ' \iff \exists i\in {\mathbb {N}}: \forall j\in {\mathbb {N}}, \alpha _j=\left\{ \begin{array}{ll} \alpha '_j &{} \hbox {if } j<i,\\ \alpha '_i+\alpha '_{i+1} &{}\hbox {if } j=i, \\ \alpha '_{j+1} &{}\hbox {if } j>i. \end{array} \right. \end{aligned}$$

If $\alpha$ and $\alpha '$ are partitions, $\alpha \preccurlyeq \alpha '$ means that $\alpha '$ is a finer partition than $\alpha$,^4^ but in our case the sequences $\alpha$ and $\alpha '$ can take negative values so that they are not partitions in general.

Remark that, setting $\Vert u\Vert _{l^1}=\sum _{n\in {\mathbb {N}}} |u_n|$,

$$\begin{aligned}u\preccurlyeq v\implies \Vert u\Vert _{l^1}\le \Vert v\Vert _{l^1}.\end{aligned}$$

The following lemma explains in which sense writing *g* as the product $c^1\phi ^1(g)\dots c^k\phi ^k(g)$ makes it ‘shorter’ than writing it as $a_1(g)^{\alpha _1(g)}\dots a_{\ell (g)}(g)^{\alpha _{\ell (g)}(g)}$.

#### Lemma 5.3

Let $x_j\in {\mathcal {P}}$, $g\in {\mathbb {F}}_{ {\mathcal {P}}}$ and $h\in {\mathbb {F}}_{ {\mathbb {F}}_{ \{1, \dots j-1 \} }}$. Then,

$$\begin{aligned} \alpha ^j(c^j(h))\preccurlyeq \alpha (h ) \qquad \hbox {and} \qquad \alpha ^j( c^j\circ \phi ^j(g) )= \alpha ^j( \pi ^{\le j} (g) ) \preccurlyeq \alpha ^j(g).\end{aligned}$$

In particular,

$$\begin{aligned} \Vert \alpha ^j( c^j\circ \phi ^j(g) )\Vert _{l^1}\le \Vert \alpha ^j(g)\Vert _{l^1}. \end{aligned}$$

#### Proof

First, remark that for $g\in {\mathbb {F}}_{ {\mathcal {P}}}$, if one can find a sequence $u=(u_1,\dots ,u_n,0,\dots )$ and $g_1,\dots , g_{n+1}\in {\mathbb {F}}_{ {\mathcal {P}}{\setminus } \{ x_{j}\} }$ such that $g=g_1x_j^{u_1}g_2 x_j^{u_2}\dots x_j^{u_n} g_{n+1}$, then $\alpha ^j(g)\preccurlyeq u$.

Secondly, remark that for $h\in {\mathbb {F}}_{ {\mathbb {F}}_{ \{1, \dots j-1 \} }}$,

9 $$\begin{aligned} c^j(h)=\prod _{i=1}^{\ell (h)} (a_i(h) x_j a_i(h)^{-1})^{\alpha _i(h) }=\prod _{i=1}^{\ell (h)} a_i(h) x_j^{\alpha _i(h) }a_i(h)^{-1}. \end{aligned}$$

From these two facts, we deduce the first relation

$$\begin{aligned} \alpha ^j(c^j(h))\preccurlyeq \alpha (h ). \end{aligned}$$

From the first fact, we also deduce that for all *g*, *h*, $\alpha ^j(gh)\preccurlyeq \alpha ^j(g) \cdot \alpha ^j(h)$, where $\cdot$ is the usual concatenation of finite sequences. In particular, for $g\in {\mathbb {F}}_{ \{1, \dots j-1 \} }$, $\alpha ^j(gh)=\alpha ^j(h)$, and with (8) we obtain the second relation

$$\begin{aligned} \alpha ^j(c^j\circ \phi ^j(g) )=\alpha ^j(\pi ^{<j}(g)^{-1}\pi ^{\le j}(g) )=\alpha ^j(\pi ^{\le j}(g) )). \end{aligned}$$

Finally, from the definition of $\alpha ^j$ there exists $g_1,\dots , g_{n+1}\in {\mathbb {F}}_{ {\mathcal {P}}{\setminus } \{ x_{j}\} }$ such that

$$\begin{aligned}g=g_1x_j^{\alpha ^j_1(g)}g_2 x_j^{\alpha ^j_2(g)}\dots x_j^{\alpha ^j_n(g)} g_{n+1},\end{aligned}$$

and $\alpha ^j_l(g)=0$ for $l>n$. Thus,

$$\begin{aligned}\pi ^{\le j}(g)= \pi ^{\le j}(g_1)x_j^{\alpha ^j_1(g)}\pi ^{\le j}(g_2) x_j^{\alpha ^j_2(g)}\dots x_j^{\alpha ^j_n(g)} \pi ^{\le j}(g_{n+1}), \end{aligned}$$

and we can use again the first fact to deduce that $\alpha ^j(\pi ^{\le j}(g))\preccurlyeq \alpha ^j(g)$, which concludes the proof. $\square$

We will use this lemma in the following way. Given the set ${\mathcal {P}}$, choose a specific order on ${\mathcal {P}}$ with some geometric relevance. Then, instead of studying $\alpha ^j( c^j\circ \phi ^j(g) )$, study instead the sequence $\alpha ^j(\pi ^{\le j}(g))$. If *g* is given as the homotopy class of $\gamma$ in $\pi _1({\mathcal {P}})$, then $\pi ^{\le j}(g)$ is given as the homotopy class of $\gamma$ in $\pi _1(\{x_1,\dots , x_j\} )$, which might be easier to study if the order has been chosen appropriately.

When an order is fixed on ${\mathcal {P}}=\{x_1<x_2<\dots \}$, we will write ${\mathcal {P}}_{x_j}=\{x_1,\dots , x_j\}$.

## Relations with Paths

In this section, we let ${\mathcal {P}}$ be a finite (deterministic) subset of $\mathbb {R}^2\setminus \{0\}$, and $\gamma :[0,1]\rightarrow \mathbb {R}^2\setminus {\mathcal {P}}$ be a continuous function satisfying $\gamma (0)=0$. It is assumed that there is no pair of distinct points $x,y\in {\mathcal {P}}$ aligned with 0 (i.e. such that ${\widehat{x0y}}=0$), and that there is no pair of distinct points $x,y\in {\mathcal {P}}$ with equal norms. One can think about $\gamma$ as being our Brownian motion *X*, and about ${\mathcal {P}}$ as one of our Poisson processes ${\mathcal {P}}_K$, but the results hold with full generality.

For $x,y\in \mathbb {R}^2$, we use the notation [*x*, *y*] for the oriented line segment from *x* to *y*, and $\gamma \cdot \gamma '$ for the concatenation of $\gamma$ with $\gamma '$, when the endpoint of $\gamma$ is equal to the starting point of $\gamma '$. For purpose of notation, it if often practical to fix a parametrisation of the oriented curves we deal with, but the specific choice doesn’t matter and we do not make them explicit.

For all $0\le s<t\le 1$, we define $\gamma ^{s,t}$ the loop $[0,\gamma _s]\cdot \gamma _{[s,t]}\cdot [\gamma _t,0]$. We also set $\gamma ^t=\gamma ^{0,t}$, and $\gamma ^{s,t}=(\gamma ^{t,s})^{-1}$ for $s>t$.

The homotopy class in $\pi _1({\mathcal {P}})$ of a loop $\gamma$ in $\mathbb {R}^2\setminus {\mathcal {P}}$ is written $[\gamma ]$, its homotopy class in $\pi _1({\mathcal {P}}_x)$ is written $[\gamma ]_x=\pi ^{\le x}([\gamma ])$, and its homotopy class in a more general subset ${\mathcal {P}}'\subseteq {\mathcal {P}}$ is written $[\gamma ]_{{\mathcal {P}}'}$.

Remark that $[\gamma ^{s,t}]$ is ill-defined as soon as one of the line segments $[0,\gamma _s]$ and $[0,\gamma _s]$ intersects ${\mathcal {P}}$. Since we ultimately want $\gamma$ to be a Brownian motion, the set of times *s* and *t* for which this happens is uncountable, and it is not possible to extend $[\gamma ^{s,t}]$, or even $[\gamma ^t]$, by right or left continuity. In the following, each time we write $[\gamma ^{s,t}]$ or $[\gamma ^{s,t}]_x$ in an equation, it is implicitly assumed that the equation holds provided that these classes are well-defined.

Remark that $[\gamma ^{s,t}]_{x}=[\gamma ^{s,u}]_{x}[\gamma ^{u,t}]_{x}$, and in particular $[\gamma ^{s,t}]_{x}=[\gamma ^{s}]^{-1}_{x}[\gamma ^{t}]_{x}$.

### Following the Cayley geodesic along the path

Let us set $h_0=1,\dots , h_N=[\gamma ]$ the geodesic walk from 1 to $[\gamma ]$ in the Cayley graph $\Gamma$ of ${\mathbb {F}}_{ {\mathcal {P}}}\cong \pi _1({\mathcal {P}})$, with respect to the generating family ${\mathcal {P}}$, and with multiplication on the right: $g,g'$ is an edge of $\Gamma$ if $g=g'y^{\pm 1}$ for some $y\in {\mathcal {P}}$. In other words, $h_i$ is the prefix of length *i* of the word

$$\begin{aligned} \prod _{j=1}^{\ell (g)} a_i(g)^{\alpha _i(g)}.\end{aligned}$$

For $i\in \{0,\dots , N\}$, we also define $(y_i,\varepsilon _i)\in {\mathcal {P}}\times \{\pm 1\}$ the unique pair such that $h_i=h_{i-1}y_i^{\varepsilon _i}$ and

$$\begin{aligned} T_i=\inf \{t\in [0,1]: [\gamma ^t]=h_i\}. \end{aligned}$$

Be careful that, when $\gamma$ is random, the $T_i$ are, in general, *not* stopping times with respect to the filtration ${\mathcal {F}}=({\mathcal {F}}_t)_{t\in [0,1]}$ generated by $\gamma$: they are only stopping times with respect to the enlarged filtration $(\sigma ({\mathcal {F}}_t,[\gamma ]) )_{t\in [0,1]}$.

Remark that $T_0=0<T_1<\dots <T_N$. Indeed, the stochastic process $([\gamma ^t])_{t\in [0,1]}$ has steps in $\Gamma$, in the sense that for any $s<t$, there exists $n\in {\mathbb {N}}$ and $s=s_0<s_1<\dots <s_n=t$ such that $([\gamma ^{s_i}],[\gamma ^{s_{i+1}}])$ is an edge of $\Gamma$. In particular, for any $g\in \Gamma \setminus \{1\}$ and any set $\Gamma '\subset \Gamma \setminus \{1,g\}$ which disconnects 1 from *g*, $[\gamma ^t]=g\implies \exists s\in (0,t): [\gamma ^s]\in \Gamma '$. Since $\Gamma$ is a tree, for all $i\in \{2,\dots N\}$, $\{h_{i-1}\}$ disconnects $h_i$ from 1 in $\Gamma$ and we deduce that indeed $0=T_0<T_1<\dots <T_N$.

Define

10 $$\begin{aligned} \delta =\delta ({\mathcal {P}})=\min \{d(x,y): x,y\in {\mathcal {P}}\cup \{0\}, x\ne y\}, \end{aligned}$$

and

$$\begin{aligned} \theta _0=\frac{1}{2} \min \{|{\widehat{x0y}}|: x,y\in {\mathcal {P}}, x\ne y \}. \end{aligned}$$

For $i\in \{0,\dots , N\}$, we set $T_i^-\le T_i\le T_i^+$ such that $\widehat{\gamma _{T_i^-}0y_i}\ne 0$, $\widehat{\gamma _{T_i^+}0y_i}\ne 0$, $[\gamma ^{T_i^-}]=h_{i-1}$, $[\gamma ^{T_i^+}]=h_i$, and for all $t\in [T_i^-,T_i^+]$, $\gamma _t\notin \partial B(y_i,\delta )$ and $\widehat{\gamma _{t}0y_i}<\theta _0$.

Then, $$T_0\le T_0^+\le T_1^-\le T_1\le T_1^+\le T_2^-\le \dots \le T_N^+$$.

For $i\in \{1,\dots , N\}$, we set $U_i$ the possibly infinite time

$$\begin{aligned} U_i=\inf \{t>T_i: d(\gamma _t,y_i)\ge \delta \}. \end{aligned}$$

#### Lemma 6.1

Let $i<k$ be such that $y_i=y_k$. Assume that there exists $j_0$ such that $i<j_0<k$ and and $y_{j_0}\ne y_{i}$. Then, $U_i<T^-_{k}$.

#### Proof

Let $l=\min \{ j>i: y_j\ne y_i\}-1=\max \{j\ge i: \forall j'\le j, y_{j'}=y_i\}$ and $m=\min \{j>l: y_j=y_i\}$. Then, $l\ge i$ and the existence of $j_0$ ensures $m\le k$. Thus, $U_i\le U_l$ and $T^-_m\le T^-_k$, and it suffices to show that $U_{l}<T^-_m$. We assume that $\gamma _{T_l}$ and $\gamma _{T_m}$ both lie inside $B(y_i,\delta )$ since otherwise the result is trivial.

Then, $T_m^-<T_m$, $T_l^+>T_l$, and $[\gamma ^{T_m^-}]=h_{m-1}$. In particular, $[\gamma ^{T^+_l,T^-_l}]=h_l^{-1}h_{m-1}\ne 1$. Since $y_n\ne y_i$ for all $n\in \{l+1,\dots ,m-1\}$, the word

$$\begin{aligned} y_{l+1}^{\varepsilon _{l+1}}\dots y_{m-1}^{\varepsilon _{m-1}}\in {\mathbb {F}}_{{\mathcal {P}}\setminus \{y_i\} },\end{aligned}$$

which is equal to $h_l^{-1}h_{m-1}\ne 1$ when seen in $\pi _1({\mathcal {P}})\cong {\mathbb {F}}_{{\mathcal {P}}}\supset {\mathbb {F}}_{{\mathcal {P}}{\setminus } \{y_i\} }$, is also equal to $[\gamma ^{T^+_l,T^-_m}]_{{\mathcal {P}}\setminus \{y_i\} }$ when seen in $\pi _1({\mathcal {P}}\setminus \{y_i\})$. It follows that $[\gamma ^{T^+_l,T^-_m}]_{{\mathcal {P}}\setminus \{y_i\} }\ne 1$, and therefore $\gamma ^{T^+_l,T^-_m}$ is non-contractible in $E=\mathbb {R}^2\setminus ({\mathcal {P}}\setminus \{y_i\})$. The conditions on the angles are such that the triangle with vertices 0, $T^+_l$ and $T^-_m$ does not contain any points of ${\mathcal {P}}{\setminus } \{y_i\}$. Thus, the loop $\gamma ^{T^+_l,T^-_m}$ can be continuously deformed in *E* into $\gamma '=\gamma _{|[T^+_l,T^-_{m}]}\cdot [\gamma _{T^-_{m} },\gamma _{T^+_l}]$. Since $\gamma ^{T^+_l,T^-_m}$ is not contractible in *E*, $\gamma '$ is also non-contractible in *E*. In particular, since the open ball $B(y_i,\delta )$, which is contractible, is included in *E*, $\gamma '$ is not included in $B(y_i,\delta )$. Thus, either $\gamma _{|[T^+_l,T^-_{m}]}$ or $[\gamma _{T^+_{m} },\gamma _{T^-_l}]$ is not included in $B(y_i,\delta )$. In both cases, we can deduce that there exists $s\in [T^+_l,T^-_m]$ such that $\gamma _s\notin B(y_i,\delta )$. Since $U_i$ is smaller than such an *s*, we deduce that $U_i<T^-_m$. $\square$

### Half-turns

**Fig. 6:**
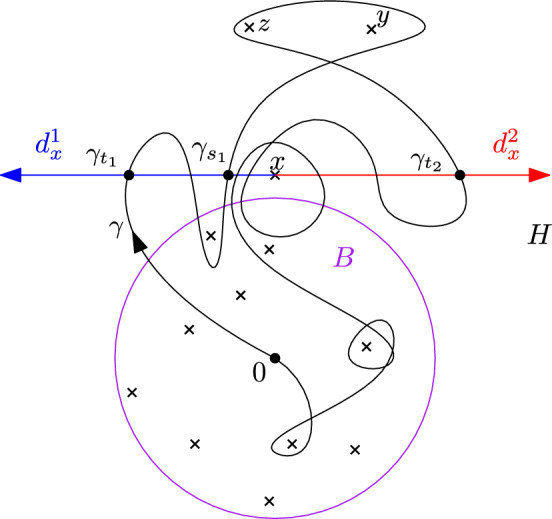
There is no universal bound on the number of times the letter *x* appears in $[\gamma ^{t_i,t_{i+1}}]$, but it can appear at most once in $[\gamma ^{t_i,t_{i+1}}]_x$. In the pictured case, $[\gamma ^{s_1,t_2}]=w_1xw_2y^{-1}w_3x^{-1}w_4z^{-1}xw_5$ for some words $w_1,\dots ,w_5\in {\mathcal {F}}_{{\mathcal {P}}_x{\setminus } \{x\}}$, but $[\gamma ^{s_1,t_2}]_x$ is simply given by $w'_1xw'_2$, with $w'_1,w'_2\in {\mathcal {F}}_{{\mathcal {P}}_x{\setminus } \{x\}}$

For $x\in {\mathcal {P}}$, we now define an integer $\theta _{\frac{1}{2}}(x,\gamma )$, the *number of half-turns of* $\gamma$ *around* *x*. Let $d_x^1$ and $d_x^2$ be the two half-lines delimited by *x* and orthogonal to the vector from 0 to *x* (see Fig. 6 below). Let $t_0=0$ and $t_1$ be the (possibly infinite) first time $\gamma$ hits $d_x^1$. Times $t_2,t_3,\dots$ are then defined recursively by the formulas

11 $$\begin{aligned} t_{2i}=\inf \{t>t_{2i-1}: \gamma _t\in d_x^2\},\ t_{2i+1}=\inf \{t>t_{2i}: \gamma _t\in d_x^1\}. \end{aligned}$$

Only finitely many of these times are less than 1, after which they are all infinite. The integer $\theta _{\frac{1}{2}}(x)=\theta _{\frac{1}{2}}(x,\gamma )$ is then defined as the maximal index *i* such that $t_i$ is finite, plus 1. This additional 1 is to account for the potential winding of $\gamma$ before it reaches $d_x^1$ for the first time.

In the following, the set ${\mathcal {P}}$ is ordered by the norm of its elements,

$$\begin{aligned} x<y\iff \Vert x\Vert _2<\Vert y\Vert _2.\end{aligned}$$

Since we assumed that all the points of ${\mathcal {P}}$ have different norms, this order is total.

What is somehow the key idea of this paper is that when ${\mathcal {P}}$ is endowed with this order, the number of half-turns $\theta _{\frac{1}{2}}(x,\gamma )$ is an upper bound for $\Vert \alpha ^x([\gamma ]_x)\Vert _{l^1}=\Vert \alpha ^x(c^x\circ \phi ^x( [\gamma ]))\Vert _{l^1}$. Let us remark, in particular, that $\theta _{\frac{1}{2}}(x,\gamma )$, as opposed to $\Vert \alpha ^x([\gamma ]_x)\Vert _{l^1}$, does not depend on ${\mathcal {P}}$ but only on *x* and $\gamma$, which makes it much easier to control when $\gamma$ is a Brownian motion.

#### Proposition 6.2

The following inequality holds:

$$\begin{aligned} \Vert \alpha ^x( [\gamma ]_x )\Vert _{\ell ^1} \le \theta _{\frac{1}{2}}(x,\gamma ).\end{aligned}$$

#### Proof

Set $t_0=0$, $t_{\theta _{\frac{1}{2}}(x,\gamma )}=1$. Since $[\gamma ]_x =[\gamma ^{t_0,t_1}]_x[\gamma ^{t_1,t_2}]_x \dots [\gamma ^{t_{n-1},t_n}]_x$ with $n=\theta _{\frac{1}{2}}(x, \gamma )$, and since

$$\begin{aligned}\Vert \alpha ^x(gg')\Vert _{\ell ^1}\le \Vert \alpha ^x(g)\Vert _{\ell ^1}+ \Vert \alpha ^x(g')\Vert _{\ell ^1}\end{aligned}$$

for all $g,g'$, it suffices to prove that $\Vert \alpha ^x([\gamma ^{t_i,t_{i+1}}]_x) \Vert _{\ell ^1}$ cannot exceed 1.

We recommend the reader to convince him or herself about the truth of this before looking at the proof. Figure 6 pictures the proof. We assume *i* is odd and $i\ne n-1$. The case when *i* is even, $i\ne 0$ and $i\ne n-1$ is dealt with by switching $d^1_x$ and $d^2_x$. Minor modifications, which are left to the reader, are required for the cases $i=0$ and $i+1= n$.

Since *i* is odd, $\gamma _{t_i}$ lies in $d^1_x$. Set

$$\begin{aligned} s_i=\sup \{t<t_{i+1}: \gamma _s\in d^1_x\}. \end{aligned}$$

Then, $\gamma _{|[t_i,s_i]}$ takes value on $\mathbb {R}^2\setminus d^2_x$. Let *B* be the closed ball centered at 0 and with radius $\frac{ \Vert x\Vert +\max \{\Vert y\Vert : y\in {\mathcal {P}}_x{\setminus } \{x\} \}}{2}$. In particular, *B* contains all the points of ${\mathcal {P}}_x$ but *x*, and $B'=B\setminus {\mathcal {P}}_x$ is a deformation retract of $\mathbb {R}^2\setminus ({\mathcal {P}}_x \cup d^2_x)$. Thus, we can continuously deform the loop $\gamma ^{t_i,s_i}$, inside $\mathbb {R}^2\setminus ({\mathcal {P}}_x \cup d^2_x)$, hence inside $\mathbb {R}^2 {\setminus } {\mathcal {P}}_x$, into a loop $\gamma '$ based at 0 and with values in $B'$.

It follows that the classes $[\gamma ^{t_i,s_i}]_{{\mathcal {P}}_x}$ and $[\gamma ^{t_i,s_i}]_{{\mathcal {P}}_x\setminus \{x\} }$ agree (using, once again, the identifications and inclusions $\pi _1({\mathcal {P}}_x {\setminus } \{x\})\cong {\mathbb {F}}_{{\mathcal {P}}_x {\setminus } \{x\}}\subset {\mathbb {F}}_{{\mathcal {P}}_x} \cong \pi _1({\mathcal {P}}_x )$), and therefore $\alpha ^x([\gamma ^{t_i,s_i}]_{x})=0$.

Let us now look at $[\gamma ^{s_i,t_{i+1}}]_{x}$. The path $\gamma _{[s_i,t_{i+1}] }$ is contained in one of the two closed half-spaces $H,H'$ delimited by the line $d^1_x\cup d^2_x$. If if lies on *H*, one can repeat the argument above, and deduce that $\alpha ^x([\gamma ^{s_i,t_{i+1}}]_{x})=0$. Otherwise, since there is no point in ${\mathcal {P}}_x \cap H'$ but *x*, and since $H'\setminus \{x\}$ is contractible, one can deform $\gamma _{[s_i,t_{i+1}] }$, continuously and with fixed endpoints, in $H'\setminus {\mathcal {P}}_x$, into any curve with the same endpoints and staying inside $H'\setminus \{x\}$. Replacing it with a curve $\gamma '$ with monotonic angle around 0, it is clear that $\Vert \alpha ^x(g^{s_i,t_{i+1} }_x)\Vert _{\ell ^1}=1$, which concludes the proof. $\square$

Let $x\in {\mathcal {P}}$, $g\in {\mathbb {F}}_{ {\mathcal {P}}}$. For a positive integer *i*, we now define $S^{x}_{i}(g)$ as the sum of all but the $i-1$ largest values of the sequence $|\alpha ^x(g)|$. In particular, $S^{x}_{1}(g)= \Vert \alpha ^x(g)\Vert _{\ell ^1}$.

#### Definition 6.3

Let $n=\max \{i: \alpha ^x_i(g)\ne 0\}$, and $\sigma \in S_n$ be such that

$$\begin{aligned}|\alpha ^x_{\sigma (1)}(g)| \ge |\alpha ^x_{\sigma (2)}(g)| \ge \dots \ge |\alpha ^x_{\sigma (n)}(g)|.\end{aligned}$$

Extend $\sigma$ into a bijection of ${\mathbb {N}}^*$ by setting $\sigma (i)=i$ for $i>n$.

Then, we define

$$\begin{aligned} \beta ^x_k(g)=\alpha ^x_{\sigma (k)}(g) \quad \hbox {and} \quad S^{x}_{i}(g)=\sum _{k=i}^{n} |\beta ^x_k(g)|. \end{aligned}$$

#### Remark 6.4

Because of the absolute values, the sequence $\beta ^x(g)=(\beta ^x_i(g))_{i\ge 1}$ is not always uniquely determined by (*x*, *g*). One can for example enforce the condition

$$\begin{aligned} \beta ^x_i(g)<0<\beta ^x_j(g)=-\beta ^x_i(g) \implies i<j \end{aligned}$$

in order to get unicity, but the peculiar choice of $\beta ^x(g)$ we make does not play a specific role in the remaining part of the paper.

#### Lemma 6.5

Let $u_1,\dots , u_n$ be a finite sequence of positive real numbers and *i* a positive integer.

Let $\sigma$ be a permutation of $\{1,\dots , n\}$ which is such that $u_{\sigma (1)}\ge u_{\sigma (2)}\ge \dots u_{\sigma (n)}$, and let

$$\begin{aligned}S_i=\sum _{k=i}^n u_{\sigma (k)},\end{aligned}$$

that is $S_i$ is the sum of all but the $i-1$ largest elements of the sequence.

Then, there exist $0=j_1\le j_2\le \dots \le j_{i+1}\le n$ such that for all $l\in \{1,\dots , i\}$,

$$\begin{aligned}\sum _{k=j_l+1}^{j_{l+1}} u_k \ge \frac{ S_i}{i}.\end{aligned}$$

#### Proof

Define recursively $j_l$ the minimal index $j\ge j_{l-1}$ such that

$$\begin{aligned}\sum _{k=j_{l-1}+1}^{j} u_k \ge \frac{ S_i}{i},\end{aligned}$$

or $j_l=\infty$ if such an index does not exist (in particular, if $j_{l-1}=\infty$), and assume by contradiction that $l_0:=\min \{l: j_l=\infty \}$ satisfies $l_0\le i$. For all $l<l_0$,

$$\begin{aligned}\sum _{k=j_{l-1}+1}^{j_l} u_k\le \sum _{k=j_{l-1}+1}^{j_l-1} u_k+u_{j_l}\le \frac{ S_i}{i} +u_{j_l}.\end{aligned}$$

By summing over $l\in \{1,\dots ,l_0-1\}$, we deduce that

$$\begin{aligned} \sum _{k=1}^{j_{l_0-1}} u_k\le (l_0-1)\frac{ S_i}{i}+ \sum _{k=1}^{ l_0-1 } u_{j_l} \le \frac{i-1}{i} S_i + (S_1-S_{l_0} )\le S_1-\frac{S_i}{i}.\end{aligned}$$

It follows that

$$\begin{aligned} \sum _{k=j_{l_0-1}+1}^n u_k=S_1-\sum _{k=1}^{j_{l_0-1}} u_k\ge \frac{S_i}{i}. \end{aligned}$$

We deduce that $j_{l_0}\le n$, which is in contradiction with the assumption that $j_{l_0}=\infty$. $\square$

Finally, one can deduce the following proposition.

#### Proposition 6.6

Let $x\in {\mathcal {P}}$, and *N*, *i* be two positive integers. Assume that $S^{x}_{i}([\gamma ]_x)\ge N$.

Then, there exist $0\le u_1\le \dots \le u_i= 1$ such that for all $k\in \{1,\dots , i-1\}$,

$$\begin{aligned}\theta _{\frac{1}{2}}(x,\gamma ^{u_k,u_{k+1}})\ge \tfrac{N}{i} \quad \hbox { and }\quad d(\gamma _{u_{k}},x)\ge \delta .\end{aligned}$$

#### Proof

We will show that there exist $0<t_1<s_1<u_1<t_2<\dots<u_{i-1}<t_i<s_i=1$ such that

- For all $$k\in \{1,\dots , i\}$$, $$\theta _{\frac{1}{2}}(x,\gamma ^{t_k,s_{k}})\ge \tfrac{N}{i}$$,
- For all $$k\in \{1,\dots , i-1\}$$, $$d(\gamma _{u_{k}},x)\ge \delta $$.

Since $\theta _{\frac{1}{2}}(x,\gamma ^{t,s})$ is monotonic in *s* and *t*, the two properties then still hold with $s_k$ replaced by $u_k$ and $t_k$ replaced by $u_{k-1}$, which allows to conclude. By Proposition 6.2, the first of these two conditions can be replaced with the condition that for all $k\in \{1,\dots , i\}$,$\Vert \alpha ^x([\gamma ]^{t_x,s_k}_x)\Vert _{\ell ^1}\ge \tfrac{N}{i}$.

Let $n=\# I^x([\gamma ]_x)$, and for $l\in \{1,\dots , n\}$ let $p^x_l$ be the length of the largest prefix *g* of $[\gamma ]_x$ such that $\alpha ^x_{l}(g)=0$, and $q^x_l$ be the length of the smallest prefix *g* of $[\gamma ]_x$ such that $\alpha ^x_{l}(g)=\alpha ^x_{l}([\gamma _x])$. For example, if $[\gamma ]_x=x^3y^4 z x^5y$, $p^x_1=0, q^x_1=3, p^x_2=3+4+1$, and $q^x_2=3+4+1+5$. Then, $p^x_1<q^x_1<p^x_2<\dots <q^x_n$ and $[\gamma ^{T^-_{p^x_l+1},T^+_{q^x_l}}]_x=x^{\alpha ^x_{l}([\gamma ]_x)}$.

By Lemma 6.5, there exist $0=j_1\le \dots \le j_{i+1}\le n$ such that for all $l\in \{1,\dots , i\}$,

$$\begin{aligned} \sum _{k=j_l+1}^{j_{l+1}} |\alpha ^x_{k}([\gamma ]_x )|\ge \tfrac{N}{i}. \end{aligned}$$

The first property then holds with $$t_l=T^-_{p^x_{j_l+1}+1} $$ and $$s_l=T^+_{q^x_{j_{l+1}}} $$.

As for the $u_l$, their existence is then ensured by Lemma 6.1. $\square$

## The Case of Brownian Paths

### Minimal spacing in a Poisson set

We finally consider the case given by ${\mathcal {P}}={\mathcal {P}}_K$. First, we need to get a bound on $\delta$ the minimal distance between two points in ${\mathcal {P}}_K\cup \{0\}$. Since ${\mathcal {P}}_K$ is infinite, $\delta ({\mathcal {P}}_K)$ is actually vanishing. Fortunately, one can freely replace ${\mathcal {P}}_K$ with its intersection with some large ball containing the Brownian motion *X*.

With this in mind, we set $F_R$ the event

$$\begin{aligned} F_R: \Vert X\Vert \le R,\end{aligned}$$

where $\Vert X\Vert$ is the maximum of $t\mapsto \Vert X_t\Vert$ and

$$\begin{aligned}{\mathcal {P}}_K^R={\mathcal {P}}_K\cap B(0,R).\end{aligned}$$

For a given *R*, the cardinal of ${\mathcal {P}}_K^R$ is equivalent (in distribution) to $\pi R^2 K$ as *K* goes to infinity, and we will see that $\delta ({\mathcal {P}}_K^R)$ is of order at least $K^{-1}$, so we also set

$$\begin{aligned} E_R: \# {\mathcal {P}}_K^R \le 4R^2 K\log (K) \hbox { and } \delta ({\mathcal {P}}_K^R)\ge (K\log (K))^{-1}. \end{aligned}$$

We could have replaced $\log (K)$ with any function *f* diverging slowly toward infinity, the goal being that $E_R$ has probability arbitrary close to 1 when *K* goes to infinity.

#### Lemma 7.1

As *K* goes to infinity,

$$\begin{aligned}{\mathbb {P}}\big (\delta ({\mathcal {P}}^R_K) \le (K \log (K))^{-1} \big )\longrightarrow 0.\end{aligned}$$

#### Proof

We cover *B*(0, *R*) with $N=\lfloor C (K\log (K))^{2} \rfloor$ balls $B_1,\dots , B_{N}$ of radius $(K\log (K))^{-1}$ (with *C* a constant depending only on *R*). This can be done for example by choosing the points on a properly scaled grid. Let then $B'_1,\dots , B'_{N}$ be the balls with the same centers $x_1,\dots , x_{N}$ and radius $2(K\log (K))^{-1}$.

If $\delta ({\mathcal {P}}^R_K) \le (K \log (K))^{-1}$, there exist two distinct points $x,y\in {\mathcal {P}}^R_K\cup \{0\}$ at distance less than $(K\log (K))^{-1}$ from each other. Let then *i* be such that $x\in B_i$. Then, both *x* and *y* lie in $B'_i$, and we deduce that

$$\begin{aligned} \{ \delta ({\mathcal {P}}^R_K) \le (K \log (K))^{-1}\}\subseteq & {} \{ \exists i\in \{1,\dots , N\}: \# ({\mathcal {P}}_K\cap B'_i)\ge 2\}\\ {}&\cup&\{ {\mathcal {P}}_K\cap B(0,(K\log (K))^{-1})\ne \emptyset \}. \end{aligned}$$

For a given *i*, $\# ({\mathcal {P}}_K\cap B'_i)$ is a Poisson variable with intensity $K\times \pi (K\log (K))^{-2}=\pi K^{-1}\log (K)^{-2}$, the probability that it is at least 2 is of order $K^{-2}\log (K)^{-4}$, so that

$$\begin{aligned} {\mathbb {P}}\Big (&\exists i\in \{1,\dots , \lfloor C (K\log (K))^{2} \rfloor \}: \# ({\mathcal {P}}_K\cap B'_i)\ge 2\Big )\\&\le C' (K\log (K))^{2} K^{-2}\log (K)^{-4}=C' \log (K)^{-2} \underset{K\rightarrow \infty }{\rightarrow }\ 0. \end{aligned}$$

It is left to the reader to show that the probability of the event $\{ {\mathcal {P}}_K\cap B(0,(K\log (K))^{-1})\ne \emptyset \}$ is small as well. $\square$

### Controlling the $\beta ^{x}_{i}$

It is now time to use the fact that our path *X* is Brownian. Let us denote $\theta (x)=\theta (x,{{\bar{X}}})$ the winding number of *X* around *x*, which counts algebraically the number of turns of *X* around *x*, and recall that $\theta _{\frac{1}{2}}(x)$ counts the absolute number of half-turns of *X* around *x*. We also set $\theta _{s,t}(x)$ as the winding number of $X^{s,t}$ around *x*. Working with a Brownian motion allows for two things. From one side, it allows to easily relates $\theta$ and $\theta _{\frac{1}{2}}$, as we will show on the next subsection. From the other side, it allows to use some already established estimations on $\theta$.

We will use the following idea. For the Brownian motion to wind a large amount of times around a given point *x*, the Brownian motion has to go extremely close to *x*. The winding are then mostly due to the small fraction of time it spend close to *x*. Since it is highly unlikely that the Brownian motion goes close to *z* twice, the winding are actually mostly due to a very small *interval* of time, during which it does not wind around any other point.

In particular, if we wait for the Brownian motion to wind a lot around *x*, and then to go far^5^ from *x*, it is unlikely that it will winds a lot around *x* after that. The following proposition gives a way to quantize this statement.

#### Lemma 7.2

Let *n* be a positive integer. Then, there exists a constant *C* such that for all $r\le 1$, for all $x\in \mathbb {R}^2$ with $\Vert x\Vert \ge r$, for all positive integers $N_1,\dots , N_n$,

$$\begin{aligned} {\mathbb {P}}\Big (&\exists s_1<t_1<u_1<s_2<\dots<u_{n-1}<s_n<t_n<1: \\&\forall i\in \{1,\dots ,n\}, |\theta _{s_i,t_i}(x)|\ge N_i, \forall i\in \{1,\dots ,n-1\}, d(X_{u_i},x)\ge r\Big ) \\&\le C \frac{ \log (r^{-1} \max _{i}\log (N_i) )^n}{N_1\dots N_n}. \end{aligned}$$

#### Proof

In [25, p. 117], we can find the following inequality on the maximal possible value of the winding function $\theta _{0,t}(1)$ as *t* varies: when $t\log (N)$ is large,

$$\begin{aligned}{\mathbb {P}}( \sup _{u\in [0,t] } \theta _u > N )\le \frac{8}{N}+\frac{2 \log (16 t\log (N))}{N}.\end{aligned}$$

From brownian scaling, we deduce that, provided $r^{-2}\log (N)$ is large, which is our case,

12 $$\begin{aligned} \sup _{x: \Vert x\Vert \ge r} {\mathbb {P}}( \sup _{t\in [0,1]} \theta _{0,t}(x) \ge N )\le C \frac{\log (r^{-2} \log (N)) }{N}, \end{aligned}$$

from which we also get

$$\begin{aligned} \sup _{x: \Vert x\Vert \ge r} {\mathbb {P}}( \sup _{s<t\in [0,1]} \theta _{s,t}(x)\ge N )\le C' \frac{\log (r^{-2} \log (N))}{N}. \end{aligned}$$

Let

$$\begin{aligned}{} & {} W_0=0,\quad V_{i+1}=\inf \{t>W_i:\exists s\in [W_i,t]\ s.t.\ |\theta _{s,t}(x)|\ge N_i\},\\{} & {} W_i=\inf \{u>V_i: d(X_{u},x)\ge r\}.\end{aligned}$$

For the event we study to happen, the stopping times $V_1,\dots ,V_n$ must all be finite. We conclude by using the Markov property *n* times at these stopping times, and by using the inequality (12) *n* times in a row. $\square$

#### Corollary 7.3

Let *n* be a positive integer and $R,\varepsilon >0$. Then, there exists a constant *C* such that for all $N_1,\dots , N_n$,

$$\begin{aligned} {\mathbb {P}}\Big (&E_R \cap \exists x\in {\mathcal {P}}_K^R , \exists s_1<t_1<u_1<s_2<\dots<u_{n-1}<s_n<t_n<1: \forall i\in \{1,\dots ,n\},\\&\quad |\theta _{s_i,t_i}(x)|\ge N_i, \forall i\in \{1,\dots ,n-1\}, d(X_{u_i},x)\ge \delta \Big )\\&\le C \frac{ K \log (K)^{n+1} \max _i \log (\log (N_i))}{N_1\dots N_n}. \end{aligned}$$

#### Proof

We enumerate ${\mathcal {P}}_K^R=\{x_1,\dots , x_{\#{\mathcal {P}}_K^R} \}$ independently from *X*. For $i>\#{\mathcal {P}}_K^R$, we set $x_i=x_1$ as a convention.

Then, for any deterministic $i\in \{1,\dots ,\lfloor K\log (K)\rfloor \}$, we can apply the previous lemma to $x=x_i$ and with $r=\min (\delta ({\mathcal {P}}^R_K),1)$.

The conclusion then follows from summation over *i*, using the fact that for *K* large enough,

$$\begin{aligned}K\log (K) \log (K\log (K))^n\le 2 K\log (K)^{n+1}.\end{aligned}$$

$$\square $$

One can finally give some controls on the $\beta ^{x,i}$. The following corollary allows to exclude three kind of events. The first convergence allows to exclude the possibility that *any* letter *x* appears once with an extremely large exponent, and then once again with a large exponent. The second convergence allows to exclude the possibility that *any* letter *x* appears twice, each time with a very large exponent. The third convergence allows to exclude the possibility that *many* letters *x* each appears twice, each time with a large exponent.

#### Corollary 7.4

Let $$\varepsilon \in (0,\tfrac{1}{6})$$. Then,

$$\begin{aligned} {\mathbb {P}}\Big (\exists x\in {\mathcal {P}}_K^R: |\beta ^x_1([{\bar{X}}]_x)|\ge K^{\frac{2}{3}}, |\beta ^x_2([{\bar{X}}]_x)|\ge K^{\frac{1}{2}-\varepsilon })\underset{K\rightarrow \infty }{\longrightarrow }0 \end{aligned}$$

and

$$\begin{aligned} {\mathbb {P}}\Big (\exists x\in {\mathcal {P}}_K^R: |\beta ^x_2([{\bar{X}}]_x)|\ge K^{\frac{1}{2}+\varepsilon }\Big )\underset{K\rightarrow \infty }{\longrightarrow }0. \end{aligned}$$

Besides,

$$\begin{aligned} {\mathbb {P}}\Big (\#\big \{ x\in {\mathcal {P}}_K^R: |\beta ^x_2([{\bar{X}}]_x)|\ge K^{\frac{1}{2}-\varepsilon }\big \} \ge K^{3\varepsilon } \Big )\underset{K\rightarrow \infty }{\longrightarrow }0. \end{aligned}$$

#### Proof

In the three cases, we control the probability of the given event intersected with $E_R\cap F_R$. Since ${\mathbb {P}}(E_R\cap F_R)$ can be made arbitrary close to 1, it is sufficient to conclude. Let $G_1$ be the event

$$\begin{aligned}\exists x\in {\mathcal {P}}_K^R: |\beta ^x_1([{\bar{X}}]_x)|\ge K^{\frac{2}{3}}, |\beta ^x_2([{\bar{X}}]_x)|\ge K^{\frac{1}{2}-\varepsilon }.\end{aligned}$$

On the event $G_1\cap E_R\cap F_R$, let us write $[{\bar{X}}]_x$ as $g_1 x^{ \beta _1 } g_2 x^{ \beta _2} g_3$ (the case $[{\bar{X}}]_x=g_1 x^{ \beta _2 } g_2 x^{ \beta _1} g_3$ is treated identically). Let $h_0,\dots , h_N$ be the geodesic path from 1 to $[{\bar{X}}]_x$, let *i*, *j*, *k*, *l* be the indices such that $h_i= g_1$, $h_j=g_1x^{ \beta _1 }$, $h_k=g_1 x^{ \beta _1 } g_2$, $h_l=g_1 x^{ \beta _1 } g_2 x^{ \beta _2}$, and for $m\in \{i,j,k,l\}$, let ${\tilde{T}}_m=T_{k_i}$ be the infimum of the times *t* such that the class of $X_{0,t}\cdot [X_{t},X_0]$ on ${\mathcal {P}}_x$ is equal to $h_i$. Then, the loop $X_{|[{\tilde{T}}_i,{\tilde{T}}_j]}\cdot [X_{{\tilde{T}}_j},X_{{\tilde{T}}_i}]$ winds $\beta _1$ times around *x*, and the loop $X_{|[{\tilde{T}}_k,{\tilde{T}}_l]}\cdot [X_{{\tilde{T}}_l},X_{{\tilde{T}}_k}]$ winds $\beta _2$ times around *x*.

According to Lemma 6.1, there exists $U\in [{\tilde{T}}_j,{\tilde{T}}_k]$ such that the distance between $X_{U}$ and *x* is at least $\delta$. Therefore, the considered event implies

$$\begin{aligned}\exists x\in {\mathcal {P}}_K, \exists s_1<t_1<u_1<s_2<t_2: |\theta _{s_1,t_1}(x)|\ge K^{\frac{2}{3}}, |\theta _{s_2,t_2}(x)|\ge K^{\frac{1}{2}-\varepsilon }, d(X_{u_1},x)\ge \delta , \end{aligned}$$

or the similar event with the exponents $\frac{2}{3}$ and $\frac{1}{2}-\varepsilon$ switched. We then apply Corollary 7.3, and we get a probability smaller than $CK \log (K)^{4} K^{-\frac{2}{3}}K^{-\frac{1}{2}+\varepsilon }$, which goes to 0 as $K\rightarrow \infty$ since $\varepsilon <\frac{1}{6}$. This proves the first convergence.

The second convergence is obtained in a similar manner, we leave it to the reader.

For the third one, we enumerate ${\mathcal {P}}^R_K=\{x_1,\dots , x_{\# {\mathcal {P}}^R_K}\}$ independently from *X*, and we set conventionally $x_i=x_1$ for $i>\# {\mathcal {P}}^R_K$ (as before).

For a given $i\in {\mathbb {N}}$, let $H_i$ be the event

$$\begin{aligned} |\beta ^{x_i}_2([{\bar{X}}]_x)| \ge K^{\frac{1}{2}-\varepsilon }.\end{aligned}$$

Applying, as before, Lemma 6.1 and then Lemma 7.2 (instead of Corollary 7.3), we obtain that

$$\begin{aligned} {\mathbb {P}}(F_R\cap E_R \cap H_i )\le C \log (K)^2 K^{-1+2\varepsilon }. \end{aligned}$$

Therefore,

$$\begin{aligned} {\mathbb {E}}[\# \{ i: H_i \hbox { holds }\} \mathbb {1}_{F_R\cap E_R} ] \le C K \log (K) \log (K)^2 K^{-1+2\varepsilon }=C\log (K)^3 K^{2\varepsilon }. \end{aligned}$$

From Markov inequality, we deduce that

$$\begin{aligned} {\mathbb {P}}\big (\# \{ i: H_i\cap F_R\cap E_R \hbox { holds }\} \ge K^{3\varepsilon } \big ) \le C\log (K)^3 K^{-\varepsilon }\underset{K\rightarrow \infty }{\longrightarrow }0. \end{aligned}$$

This concludes the proof. $\square$

The previous bounds tell us about the highest values $\beta ^x_1,\beta ^x_2,\beta ^x_3$, but it does not tell us anything about the behaviour of the ‘tails’ $S^x_i$. Controlling these tails is our next goal.

### Controlling the tails $S^x_i$

Our strategy is to apply Proposition 6.6, which relates $S^x_i$ with $\theta _{\frac{1}{2}}(x)$, to bound the probably that $\theta _{\frac{1}{2}}(x)$ is large in term of the probability that the winding number $\theta (x)$ is large, and then to use the already presented bound on the probability that $\theta (x)$ is large. In this strategy, only the middle piece, given by the following lemma, is still missing.

#### Lemma 7.5

There exists a finite constant *C* such that for all $N\ge 1$, and $x\in \mathbb {R}^2$,

$$\begin{aligned} {\mathbb {P}}(\theta _{\frac{1}{2}}(x)\ge N )\le C {\mathbb {P}}(\theta (x)\ge \sqrt{N} ).\end{aligned}$$

#### Proof

In short, it follows from the fact that $\theta (x)$ is, up to an error of $\pm 1$, the sum of $\theta _{\frac{1}{2}}(x)$ i.i.d. centered Bernoulli variables.

Let use recall that the stopping times $t_i$, defined by (11), corresponds to times when the Brownian motion hits some half-lines $d^1_x,d^2_x$. It is easily seen that the real-valued winding ${\tilde{\theta }}(x, X_{|[0,t_i]})$ and ${\tilde{\theta }}(x, X_{|[0,t_{i+1}]})$ are related by

$$\begin{aligned} \varepsilon _i:={\tilde{\theta }}(x, X_{|[0,t_{i+1} ]})-{\tilde{\theta }}(x, X_{|[0,t_{i}]})\in \{-\tfrac{1}{2}, \tfrac{1}{2} \}. \end{aligned}$$

The integer winding number $\theta (x)$ is then given by

$$\begin{aligned}\theta (x)=\sum _{i=1}^{\theta _{\frac{1}{2}}(x) } \varepsilon _i+\varepsilon _0,\end{aligned}$$

with $$\varepsilon _0\in \{-1,-\frac{1}{2},0,\frac{1}{2},1\}$$.

Since the $t_i$ are stopping times, we can apply the reflection property of the Brownian motion at the times $t_i$, with reflection around the axe $d^1_x\cup d^2_x$. We obtain a new Brownian motion with the same stopping times $t_i$, and we can deduce that, conditionally on $\theta _{\frac{1}{2}}(x)$, the variables $(\varepsilon _i)_{i\in \{1, \dots ,\theta _{\frac{1}{2}}(x)\} }$ are i.i.d. symmetric Bernoulli variables. Hence, for all $N\le M$,

$$\begin{aligned} {\mathbb {P}}\big (\theta (x)\ge \sqrt{N} \big |\theta _{\frac{1}{2}}(x)= M \big )&= {\mathbb {P}}\Big (\varepsilon _0+ \sum _{i=1}^{\theta _{\frac{1}{2}}(x) } \varepsilon _i\ge \sqrt{N}\Big |\theta _{\frac{1}{2}}(x)=M\Big )\\&\ge {\mathbb {P}}\Big (\varepsilon _0+ \sum _{i=1}^{\theta _{\frac{1}{2}}(x) } \varepsilon _i\ge \sqrt{M}\Big |\theta _{\frac{1}{2}}(x)=M\Big )=:p_M \end{aligned}$$

Since $p_M$ is positive for all $M\ge 2$ and since

$$\begin{aligned} p_M \underset{M \rightarrow \infty }{\longrightarrow }\ \int _{1}^{+\infty } \frac{e^{-2 t^2 }}{\sqrt{\pi /2}}\;\textrm{d}t> 0,\end{aligned}$$

we deduce that $\inf _{M\ge 1} p_M>0$, hence

$$\begin{aligned} \inf _{2\le N \le M} {\mathbb {P}}\big (\theta (x)\ge \sqrt{N} \big |\theta _{\frac{1}{2}}(x)= M \big )>0,\end{aligned}$$

thus

$$\begin{aligned}c:=\inf _{2\le N } {\mathbb {P}}\big (\theta (x)\ge \sqrt{N} \big |\theta _{\frac{1}{2}}(x) \ge N \big )>0.\end{aligned}$$

For any two real random variables *U*, *V*,

$$\begin{aligned}{\mathbb {P}}(U\ge u)\le \frac{{\mathbb {P}}(V\ge v ) }{{\mathbb {P}}\big ( V\ge v\big | U\ge u \big ) }.\end{aligned}$$

In particular, for all $N\ge 2$

$$\begin{aligned} {\mathbb {P}}(\theta _{\frac{1}{2}}(x)\ge N )\le \frac{{\mathbb {P}}(\theta (x)\ge \sqrt{N} ) }{ {\mathbb {P}}\big (\theta (x)\ge \sqrt{N} \big |\theta _{\frac{1}{2}}(x)\ge N \big ) } \le c^{-1}{\mathbb {P}}(\theta (x)\ge \sqrt{N} ), \end{aligned}$$

which concludes the proof. $\square$

One can now give the following bound on the tails $S^x_i$.

#### Lemma 7.6

Let $$\varepsilon \in (0,\tfrac{1}{10})$$. Then,

$$\begin{aligned} {\mathbb {P}}( \exists x\in {\mathcal {P}}_K: S^x_5([{\bar{X}}]_x )\ge K^{\tfrac{1}{2}-\varepsilon }) \underset{K\rightarrow \infty }{\longrightarrow }0 \end{aligned}$$

This lemma make us closer to the ‘much simpler problem’ we solved in Sect. 4. Instead of dealing with the whole apparitions *x* in $[{\bar{X}}]$, we will only have to deal with the apparitions that comes with a large exponent.

#### Proof

Set $N= \tfrac{K^{\frac{1}{2}-\varepsilon }}{5}$. For a given point $x\in \mathbb {R}^2$ with $\Vert x\Vert \ge \delta$, let

$$\begin{aligned}U_0=0,\quad U_{i+1}=\inf \{t: \theta _{\frac{1}{2}}(x,X^{U_i,t} )\ge N, d(X_{t},x)\ge \delta \}.\end{aligned}$$

Then, using Proposition 6.6, we have

$$\begin{aligned} {\mathbb {P}}(S^x_5([{\bar{X}}]_x )\ge K^{\frac{1}{2}-\varepsilon } )\le {\mathbb {P}}\big ( U_5 <1\big ). \end{aligned}$$

Since the $U_i$ are stopping times, one can apply the Markov property to deduce

$$\begin{aligned} {\mathbb {P}}\big ( U_5<1\big )\le {\mathbb {P}}(U_1<1)^5.\end{aligned}$$

Lemma 7.5 says that ${\mathbb {P}}(U_1<1)\le C {\mathbb {P}}(\theta (x) \ge \sqrt{N} )$, which thanks to Eq. (12) we know to be less than $C'\frac{\log (\delta ^{-2} \log (N))}{N^{\frac{1}{2}}}$.

Combining these inequalities together, we get

$$\begin{aligned} {\mathbb {P}}(S^x_5([{\bar{X}}]_x )\ge K^{\frac{1}{2}-\varepsilon } )\le C (K^{\frac{1}{2}-\varepsilon })^{-\frac{5}{2}} \log (\delta ^{-2} \log (K))^5. \end{aligned}$$

Since this holds for all $\Vert x\Vert \ge \delta$, we get

$$\begin{aligned} {\mathbb {P}}( E_R\cap F_R\cap \exists x\in {\mathcal {P}}^R_K:S^{(5)}(x,g^1_x )\ge K^{\frac{1}{2}-\varepsilon } ) \le C' R^2K\log (K)^6 K^{-\frac{5}{4}+\frac{5}{2}\varepsilon }\underset{K\rightarrow \infty }{\longrightarrow }0.\end{aligned}$$

$$\square $$

This estimation in itself is not very practical, but it can then be improved in the three following ways, which correspond to the three convergences of Corollary 7.4.

#### Proposition 7.7

Let $$\varepsilon \in (0,\tfrac{1}{20})$$. Then,

$$\begin{aligned}{} & {} {\mathbb {P}}\big ( \exists x\in {\mathcal {P}}_K:S^x_2([{\bar{X}}]_x )\ge K^{\frac{1}{2}-\varepsilon } \hbox { and } \beta ^x_1([{\bar{X}}]_x)\ge K^{\frac{2}{3}}\big )\underset{K\rightarrow \infty }{\longrightarrow }0, \\{} & {} {\mathbb {P}}\big ( \exists x\in {\mathcal {P}}_K: S^x_2([{\bar{X}}]_x )\ge K^{\frac{1}{2}+\varepsilon }\big )\underset{K\rightarrow \infty }{\longrightarrow }0, \\{} & {} {\mathbb {P}}\big ( \# \{x\in {\mathcal {P}}_K:S^x_2([{\bar{X}}]_x )\ge K^{\frac{1}{2}-\varepsilon } \}\ge K^{5\varepsilon } \big )\underset{K\rightarrow \infty }{\longrightarrow }0. \end{aligned}$$

#### Proof

The three proofs are similar, we only write the first one. Thanks to Lemma 7.6, we can assume that $S^x_5([{\bar{X}}]_x )\le K^{\frac{1}{2}-2\varepsilon }$ for all $x\in {\mathcal {P}}_K$. Then, for $S^x_2([{\bar{X}}]_x )$ to be larger than $K^{\frac{1}{2}-\varepsilon }$, $\beta ^x_2([{\bar{X}}]_x )$ has to be larger than $\frac{K^{\frac{1}{2}-\varepsilon } -K^{\frac{1}{2}-2\varepsilon } }{3}$, which is larger than $K^{\frac{1}{2}-\frac{3 \varepsilon }{2}}$ for *K* large enough. Since we also ask for $\beta ^x_1([{\bar{X}}]_x )$ to be large, we can apply the first convergence in Corollary 7.4 to conclude. $\square$

In the following, we fix some $\varepsilon \in (0,\tfrac{1}{20})$. We call $G_R$ the intersection of $F_R$, $E_R$, and the complementaries of the three events appearing in Proposition 7.7 (with the chosen $\varepsilon$). In particular, for any given *R*, $G_R$ has probability arbitrarily close to 1 when *R* is large.

#### Lemma 7.8

As $$K\rightarrow \infty $$,

$$\begin{aligned}K^{-\frac{1}{2}-\varepsilon } \# \{x\in {\mathcal {P}}: |\beta ^x_1([{\bar{X}}]_x )|> K^{\frac{1}{2}-\varepsilon }\} \hbox { and }K^{-\frac{1}{3}} \# \{x\in {\mathcal {P}}: |\beta ^x_1([{\bar{X}}]_x )| > K^{\frac{2}{3}}\} \end{aligned}$$

converge in probability towards $\frac{1}{\pi }$.

#### Proof

Let ${\mathcal {P}}'$ be the set of points *x* in ${\mathcal {P}}$ for which $S^x_2([{\bar{X}}]_x )\ge K^{\frac{1}{2}-2 \varepsilon }$. On the event $G_R$, we know that there is at most $K^{5\varepsilon }$ of them. Since $K^{-\frac{1}{2}+\varepsilon } K^{5\varepsilon }\underset{K\rightarrow \infty }{\rightarrow }\ 0$ and $K^{-\frac{1}{3}}K^{5\varepsilon } \underset{K\rightarrow \infty }{\rightarrow }\ 0$, it suffices to show that

$$\begin{aligned}K^{-\frac{1}{2}-\varepsilon } \# \{x\in {\mathcal {P}}\setminus {\mathcal {P}}': \beta ^x_1([{\bar{X}}]_x )> K^{\frac{1}{2}-\varepsilon }\} \hbox { and }K^{-\frac{1}{3}} \# \{x\in {\mathcal {P}}\setminus {\mathcal {P}}': \beta ^x_1([{\bar{X}}]_x ) > K^{\frac{2}{3}}\} \end{aligned}$$

converges in probability towards $\frac{1}{\pi }$.

Since $\sum _i |\alpha ^x_i([{\bar{X}}]_x)|= | \beta ^x_1([{\bar{X}}]_x)|+ S^x_2([{\bar{X}}]_x)$ and $\theta (x)=\sum _i \alpha ^x_i([{\bar{X}}]_x)$, we have

$$\begin{aligned} | \beta ^x_1([{\bar{X}}]_x)|- S^x_2([{\bar{X}}]_x)\le |\theta (x)|\le | \beta ^x_1([{\bar{X}}]_x)|- S^x_2([{\bar{X}}]_x).\end{aligned}$$

Thus,

13 $$\begin{aligned} \{x\in {\mathcal {P}}\setminus {\mathcal {P}}': |\theta (x)|> K^{\frac{1}{2}-\varepsilon }+ K^{\frac{1}{2}-2 \varepsilon } \} \subseteq&\{x\in {\mathcal {P}}\setminus {\mathcal {P}}' : |\beta ^x_1([{\bar{X}}]_x )|> K^{\frac{1}{2}-\varepsilon }\} \nonumber \\ {}&\subseteq \{x\in {\mathcal {P}}: |\theta (x)| > K^{\frac{1}{2}-\varepsilon }- K^{\frac{1}{2}-2 \varepsilon } \}. \end{aligned}$$

Let $D_k$ be the area of the set of points $z\in \mathbb {R}^2$ such that $|\theta (x)| \ge k$. Then $kD_k$ converges towards $\tfrac{1}{\pi }$ in $L^2$ and therefore in probability (see [27].^6^). It follows that both

$$\begin{aligned}{} & {} K^{-\frac{1}{2}-\varepsilon } \# \{x\in {\mathcal {P}}\setminus {\mathcal {P}}': |\theta (x)|> K^{\frac{1}{2}-\varepsilon }+ K^{\frac{1}{2}-2\varepsilon } \} \hbox { and }\\{} & {} K^{-\frac{1}{2}-\varepsilon } \# \{x\in {\mathcal {P}}: |\theta (x)| > K^{\frac{1}{2}-\varepsilon }- K^{\frac{1}{2}-2\varepsilon } \} \end{aligned}$$

converges in probability towards $\tfrac{1}{\pi }$ as *K* goes to infinity.

This proves the first convergence. The second is obtain in an identical way. $\square$

We now consider $H_R$ the large probability event

$$\begin{aligned} G_R&\cap&\big \{ K^{-\frac{1}{2}-\varepsilon } \# \{x\in {\mathcal {P}}: \beta ^x_1([{\bar{X}}]_x )> K^{\frac{1}{2}-\varepsilon }\} \le 1\big \}\\&\cap&\big \{ K^{-\frac{1}{3}} \# \{x\in {\mathcal {P}}: \beta ^x_1([{\bar{X}}]_x ) > K^{\frac{2}{3}}\} \le 1\big \}. \end{aligned}$$

## End of the Proof

In order to finally conclude the proof of the main theorem, we split ${\mathcal {P}}_K^R$ into a disjoint union ${\mathcal {P}}_K^R= {\mathcal {P}}^0\sqcup {\mathcal {P}}^1\sqcup {\mathcal {P}}^2\sqcup {\mathcal {P}}^3$, with

$$\begin{aligned} {\mathcal {P}}^0&=\{x \in {\mathcal {P}}_K^R : \beta ^x_1([{\bar{X}}]_x) > K^{\frac{2}{3} } \},\\ {\mathcal {P}}^1&=\{x \in {\mathcal {P}}_K^R : K^{\frac{1}{2}-\varepsilon } <\beta ^x_1([{\bar{X}}]_x) \le K^{\tfrac{2}{3}} \},\\ {\mathcal {P}}^2&=\{x \in {\mathcal {P}}_K^R : \beta ^x_1([{\bar{X}}]_x) \le K^{\frac{1}{2}-\varepsilon } \hbox { and } S^x_2([{\bar{X}}]_x) \ge K^{\frac{1}{2}-\varepsilon } \},\\ {\mathcal {P}}^3&=\{x \in {\mathcal {P}}_K^R : \beta ^x_1([{\bar{X}}]_x) \le K^{\frac{1}{2}-\varepsilon } \hbox { and } S^x_2([{\bar{X}}]_x) \le K^{\frac{1}{2}-\varepsilon } \}. \end{aligned}$$

The elements of ${\mathcal {P}}^0$ are somehow the most important, in the sense that there are the ones that we expect to contribute to the limit. Besides, for each of these points in ${\mathcal {P}}^0$, there is one particular place where it appears with a large exponent, and this place is the most important.

For $k\in \{1,\dots , \#{\mathcal {P}}^0\}$, let $y_k=x_{u(k)}$ be the $k^{\hbox {th}}$ element of ${\mathcal {P}}^0$ (with ${\mathcal {P}}^0$ inheriting the order of ${\mathcal {P}}_K$), which is also the $u(k)^{\hbox {th}}$ element of ${\mathcal {P}}_K$. For $y=y_k$, let $i_k$ be the unique index $i_k$ such that $\alpha ^{y}_{i_k}([{\bar{X}}]_{y} )=\beta _1^{y}([{\bar{X}}]_{y} )$. We write $c^{y}\circ \phi ^{y}([{\bar{X}}])$ as

$$\begin{aligned} c^{y}\circ \phi ^{y}([{\bar{X}}])=R^1_k M_k R^2_k \end{aligned}$$

with

$$\begin{aligned}{} & {} M_k= w_{k,i_k} y^{\beta _1^{y} ([{\bar{X}}]_{y}) } w_{k,i_k}^{-1}, \\{} & {} R^1_k = w_{k,1} y^{ \alpha ^{y}_{1}([{\bar{X}}]_{y} ) }w_{k,1}^{-1}\dots w_{k,i_k-1} y^{ \alpha ^{y}_{i_k-1}([{\bar{X}}]_{y} ) }w_{k,i_k-1}^{-1},\\{} & {} R^2_k=w_{k,i_k+1} y^{ \alpha ^{y}_{i_k+1}([{\bar{X}}]_{y} )} w_{k,i_k+1}^{-1} \dots w_{k,n} y^{ \alpha ^{y}_{n}([{\bar{X}}]_{y} ) }w_{k,n}^{-1}, \end{aligned}$$

with $w_{k,j}\in {\mathbb {F}}_{\{x_1,\dots , x_{u(k)-1}\} }$. Finally, let

$$\begin{aligned}P_k=\Big (\prod _{\begin{array}{c} x\in {\mathcal {P}}_K\\ y_{k-1}<x<y_k \end{array}} c^x\circ \phi ^x([{\bar{X}}])\Big ),\end{aligned}$$

so that

$$\begin{aligned} {[}{\bar{X}}]= & {} P_1 \cdot c^{y_1}\circ \phi ^{y_1}([{\bar{X}}]) \cdot \dots \cdot P_{{\#{\mathcal {P}}_0}} \cdot c^{y_{\#{\mathcal {P}}_0} }\circ \phi ^{y_{\#{\mathcal {P}}_0} }([{\bar{X}}])\cdot P_{{\#{\mathcal {P}}_0} +1}\\= & {} \Big (\prod _{k=1}^{\# {\mathcal {P}}^0} P_k\ R^1_k \ M_k\ R^2_k \Big ) P_{{\#{\mathcal {P}}_0} +1}. \end{aligned}$$

In the following, for $g_K,h_K\in \pi _1( {\mathcal {P}}_K)$, we write $g_K\approx h_K$ if for any compact and connected Lie group *G*, $\omega _K(g_K)$ converges in distribution if and only if $\omega _K(h_K)$ converges in distribution, and the limit distributions are the same if they exist. We also write $g_K\simeq h_K$ if $\omega _K(g_K h_K^{-1})$ converges in distribution towards 1, which is a stronger statement.

### Proposition 8.1

14 $$\begin{aligned} {[}{\bar{X}}]\simeq \Big (\prod _{k=1}^{\# {\mathcal {P}}^0} P_k\ M_k\ \Big ) P_{{\# {\mathcal {P}}^0}+1} \approx \prod _{k=1}^{\# {\mathcal {P}}^0}y_k^{\beta ^{y_k}_1([{\bar{X}}])} \simeq \prod _{k=1}^{\# {\mathcal {P}}^0}y_k^{\theta (y_k )} \approx \prod _{k=1}^{\# {\mathcal {P}}_K}x_k^{\theta (x_k)}. \end{aligned}$$

Remark that this proposition, together with Corollary 4.7, allows to conclude to Theorem 1, for which it would suffice to have $\approx$ relations everywhere. The arguments that we use to show that $\simeq$ relations hold are very different from the arguments that we use to show that $\approx$ relations hold: $\prod _k X_k\simeq \prod _k Y_k$ typically holds when $X_k$ is very close from $Y_k$ for all *k*, whilst $\prod _k X_k\simeq \prod _k Y_k$ typically holds when $X_k$ is very close from *a conjugate of* $Y_k$ for all *k*.

To prove Proposition 8.1, we only lack two more lemmata, in which the compact Lie group *G* finally appears.

### Lemma 8.2

Let *G* be a Lie group. For all *K*, let $n_K$ be a random integer, and let $(X_i)_{i\in \{1,\dots , n_K\} }$ and $(Y_i)_{i\in \{1,\dots , n_K+1\} }$ be two sequences of *G*-valued random variables.

Assume that the product $Y_1\dots Y_{n_K+1}$ converges in distribution towards 1 as *K* goes to infinity, and that

$$\begin{aligned}(X_i)_{i\in \{1,\dots ,n_K\}}\overset{(d)}{=}((Y_1\dots Y_i) X_i (Y_1\dots Y_i)^{-1} )_{i\in \{1,\dots ,n_K\}}. \end{aligned}$$

Then, $X_1\dots X_{n_K}$ converges in distribution if and only if $Y_1 X_1\dots Y_{n_K}X_{n_K}Y_{n_K+1}$ converges in distribution, in which case the limiting distribution are the same.

### Proof

Remark that

15 $$\begin{aligned} Y_1 X_1\dots Y_{n_K} X_{n_K}Y_{n_K+1} = \Big (\prod _{i=1}^{n_K} ((Y_1\dots Y_i) X_i (Y_1\dots Y_i)^{-1})\Big ) Y_1\dots Y_{n_K}. \end{aligned}$$

Since $Y_1\dots Y_{n_K}$ converges to 1, the left-hand-side of (15) converges if and only if

$$\begin{aligned}\Big (\prod _{i=1}^{n_K} ((Y_1\dots Y_i) X_i (Y_1\dots Y_i)^{-1})\Big )\end{aligned}$$

converges as well, and the limits are the same. This last expression is equal in distribution to the product $X_1 \dots X_{n_K}$, hence the lemma. $\square$

### Proposition 8.3

For a totally ordered finite set ${\mathcal {P}}$ and $g\in {\mathbb {F}}_{ {\mathcal {P}}}$, set $|g|_2$ be the non-negative square root of

$$\begin{aligned} \sum _{x \in {\mathcal {P}}} S^x_1(\pi ^{\le x}(g) )^2=\sum _{x \in {\mathcal {P}}} \Vert \alpha ^x(\pi ^{\le x}(g) )\Vert _{\ell ^1}^2. \end{aligned}$$

Let *G* be a compact Lie group, endowed with a biinvariant metric. There exist $C,c>0$ such that for any totally ordered finite set ${\mathcal {P}}$ with $\# {\mathcal {P}}\ge 4$, for all $K \ge 1$, all $g\in {\mathbb {F}}_{ {\mathcal {P}}}$ with $|g|_2^2\le cK^2$, and all family of ${\mathfrak {g}}$-valued random variables $(H_i)_{i\in {\mathcal {P}}}$ with support on the ball of radius $K^{-1}$ and such that for all *x*,

$$\begin{aligned} {\mathbb {E}}[H_x| \sigma ((H_y)_{y<x} ) ]=0,\end{aligned}$$

the random group morphism $h_K: {\mathbb {F}}_{ {\mathcal {P}}} \rightarrow G$ determined by $h_K(e_i)=\exp _G( H_i)$ satisfies

$$\begin{aligned} {\mathbb {E}}[ d_G(h_K(g),1)] \le C \big ( K^{-1}|g|_2+ \frac{\log (\# {\mathcal {P}})^2}{\log (\log (\# {\mathcal {P}} ) ) } K^{-2} |g|_2^2 \big ). \end{aligned}$$

The proof of this is technical, and postponed to the next section.

### Remark 8.4

The restriction $\# {\mathcal {P}}\ge 4$ is only to ensure $\log (\log ( \# {\mathcal {P}}))$ is well-defined and positive. The result remain true without this restriction if the ratio of logarithms is replaced with 1 when $\# {\mathcal {P}}< 4$.

We believe but cannot prove that the second term can actually be omitted. This is possible indeed when the group is commutative, in which case the proposition follows from a basic analysis of the variance.

When the group is not commutative, it might be simpler to prove that

$$\begin{aligned} {\mathbb {E}}[ d_G(h_K(g),1)] \le C K^{-1}|g|'_2, \end{aligned}$$

where ${|g|'}_2^2=\sum _{x\in {\mathcal {P}}} S^x_1(g)^2$, but such a bound would not be sufficient for our purpose.

Assuming it to hold, we now prove Proposition 8.1.

### Proof of Proposition 8.1 assuming Proposition 8.3

We work on the large probability event $G_R$.

Let *G* be a compact Lie group, endowed with a biinvariant metric $d_G$. Since the metric is biinvariant,

$$\begin{aligned} d_G\Big (\omega _K([{\bar{X}}]), \omega _K\big ( \big (\prod _{k=1}^{\# {\mathcal {P}}^0} P_k\ M_k\ \big ) P_{k+1} \big )\Big )&\le \sum _{k=1}^{\# {\mathcal {P}}^0} d_G(\omega _K(P_kR^1_kM_kR^2_k), \omega _K(P_kM_k))\\&\le \sum _{k=1}^{\# {\mathcal {P}}^0} (d_G(\omega _K(R_k^1),1)+d(\omega _K(R_k^2),1))\\&\le \sum _{k=1}^{\# {\mathcal {P}}^0} \sum _{i\ne i_k} |\alpha ^{y_k}_i([{\bar{X}}]_{y_k})| d_G(h_K(y_k),1)\\&\le \sum _{x\in {\mathcal {P}}^0} S^x_2([{\bar{X}}]_x ) K^{-1}. \end{aligned}$$

The third inequalities follows from the general inequality

$$\begin{aligned} d_G(1,ghg^{-1}klk^{-1})\le d_G(1,ghg^{-1})+d_G(1,klk^{-1})=d_G(1,h)+d_G(1,l).\end{aligned}$$

Since $\# {\mathcal {P}}^0\le K^{\frac{1}{3}}$ and $S^x_2([{\bar{X}}]_x)\le K^{\frac{1}{2}}$ for all $x\in {\mathcal {P}}^0$, we end up with

$$\begin{aligned} d_G\Big (\omega _K([{\bar{X}}]), \omega _K\Big ( \Big (\prod _{k=1}^{\# {\mathcal {P}}^0} P_k\ M_k\ \Big ) P_{k+1} )\Big )\le K^{-\frac{1}{6}}\underset{K\rightarrow \infty }{\longrightarrow }0. \end{aligned}$$

This proves the first relation of (14).

We now look at the second one, which follows from Lemma 8.2 if one can apply it to the variables

$$\begin{aligned} X_k=\omega _K(y_k^{\beta _1^{y_k}([{\bar{X}}])}), \qquad Y_k=\omega _K(w_{k-1, i_{k-1} }^{-1} P_k w_{k,i_k}).\end{aligned}$$

We need to check that,

16 $$\begin{aligned} (\omega _K(X_i)) _{i\in 1,\dots n_K} \overset{(d)}{=} (\omega _K( (Y_1\dots Y_i ) X_i (Y_1\dots Y_i )^{-1} ) )_{i\in 1,\dots n_K}. \end{aligned}$$

Recall that for a set of independent random variables $(Z_1,\dots , Z_n)$ which are each conjugation-invariant and measurable functions $f_1,\dots f_n:G^{n-1}\rightarrow G$ (where $f_1:\{\}=G^0\rightarrow G$ is just a constant)

$$\begin{aligned} (Z_1,\dots , Z_n)\overset{(d)}{=} & {} ( f_1 Z_1 f_1^{-1}, f_2(Z_1)Z_2 f_2(Z_1)^{-1},\dots , f_n(Z_1,\dots , Z_{n-1}) \\{} & {} Z_n f_n(Z_1,\dots , Z_{n-1})^{-1} ). \end{aligned}$$

This can be proved easily by induction. By applying this to the sequence of points in ${\mathcal {P}}_K^G$, with $f_{x_k}=Y_k$ which only depends on the previous variables, we deduce (16).

Now we need to check the second assumption of Lemma 8.2, which amounts to show that

$$\begin{aligned}\omega _K\Big (\prod _{k=1}^{\# {\mathcal {P}}^0+1} P_k \Big )\underset{K\rightarrow \infty }{\longrightarrow }1.\end{aligned}$$

We want to apply Proposition 8.3, for which we need to bound

$$\begin{aligned} \big |\hspace{-0.2cm}\prod _{k=1}^{ \# {\mathcal {P}}^0+1} P_k\big |_2^2=\sum _{x\in {\mathcal {P}}^1\sqcup {\mathcal {P}}^2\sqcup {\mathcal {P}}^3} S^x_1([{\bar{X}}]_x )^2. \end{aligned}$$

For $x\in {\mathcal {P}}_1$, we know that $\beta ^x_1([{\bar{X}}]_x)\le K^{\frac{2}{3}}$, hence that $S^x_1([{\bar{X}}]_x)\le K^{\frac{2}{3}}+K^{\frac{1}{2}+\varepsilon }$. Since we also know that $\# {\mathcal {P}}^1\le K^{\frac{1}{2}+\varepsilon }$,

$$\begin{aligned} \sum _{x\in {\mathcal {P}}^1} S^x_1([{\bar{X}}]_x)^2\le K^{\frac{1}{2}+\varepsilon } ( K^{\frac{2}{3}}+K^{\frac{1}{2}+\varepsilon })^2\sim K^{\frac{11}{6}+\varepsilon }. \end{aligned}$$

For $x\in {\mathcal {P}}^2$, we know that $S^x_1([{\bar{X}}]_x)\le 2 K^{\frac{1}{2}+\varepsilon }$. Since we also know that $\# {\mathcal {P}}^2\le K^{5\varepsilon }$,

$$\begin{aligned} \sum _{x\in {\mathcal {P}}^2} S^x_1([{\bar{X}}]_x)^2\le 4 K^{1+7\varepsilon }. \end{aligned}$$

For $x\in {\mathcal {P}}^3$, we know that $S^x_1([{\bar{X}}]_x)\le 2 K^{\frac{1}{2}-\varepsilon }$. Since we also know that $\# {\mathcal {P}}^2\le \# {\mathcal {P}} \le K\log (K)$,

$$\begin{aligned} \sum _{x\in {\mathcal {P}}^3} S^x_1([{\bar{X}}]_x)^2\le 4 \log (K) K^{2-2\varepsilon }. \end{aligned}$$

Combining this three results together, we obtain, for *K* large enough,

$$\begin{aligned} \big |\hspace{-0.2cm}\prod _{k=1}^{\# {\mathcal {P}}^0+1} P_k\big |_2^2 \le K^{2-\varepsilon }. \end{aligned}$$

This is enough to apply Proposition 8.3, and to deduce that

$$\begin{aligned} {\mathbb {E}}\Big [ d_G\Big (\omega _K\big ( \prod _{k=1}^{\# {\mathcal {P}}^0+1} P_k\big ),1\Big )\Big ]\le C( K^{-\frac{\varepsilon }{2}}+\log (K)^2 K^{-\varepsilon }) \underset{K \rightarrow \infty }{\longrightarrow }0. \end{aligned}$$

It follows that $\omega _K\Big ( \prod _{k=1}^{\# {\mathcal {P}}^0+1} P_k\Big )$ converges to 1 in probability, which proves the second relation.

The two last relations are proved in a similar, but easier, way. First,

$$\begin{aligned} d_G\Big ( \prod _{k=1}^{\# {\mathcal {P}}^0} h_K(y_k)^{\beta ^{y_k}_1([{\bar{X}}]_{y_k})}, \prod _{k=1}^{\# {\mathcal {P}}^0} h_K(y_k)^{\theta (y_k)} \Big )&\le \sum _{k=1}^{\# {\mathcal {P}}^0} |\theta (y_k)-\beta ^{y_k}_1([{\bar{X}}]_{y_k})| d_G(h_K(y_k),1)\\&\le (\# {\mathcal {P}}^0) K^{-1} \max _{x\in {\mathcal {P}}_0}(|\theta (x)-\beta ^x_1([{\bar{X}}]_{x})| )\\&\le 2 K^{\frac{1}{3}} K^{-1} K^{\frac{1}{2}-\varepsilon }\underset{K\rightarrow \infty }{\longrightarrow }0. \end{aligned}$$

The last inequality follows from the events $G_R$ (recall the first bound in Proposition 7.7) and $H_R$ (recall Lemma 7.8). This proves the third relation.

For the fourth one, we apply Lemma 8.2 once more, with

$$\begin{aligned}X_k= \omega _K( y_k^{\theta (y_k)} ),\qquad Y_k= \omega _K( \prod _{y_{k-1}< x <y_{k}} x^{\theta (x)} ).\end{aligned}$$

Here the independence assumption is even easier to check. The only problem left is to show that the second assumption of Lemma 8.2 holds, that is to show that the product $\prod _{x\in {\mathcal {P}}_K{\setminus } {\mathcal {P}}^0} h_K(x)^{\theta (x,{\bar{X}})}$ converges towards 1 (recall that $h_K$ is the map from ${\mathcal {P}}_K$ to *G* such that $(x,h_K(x))\in {\mathcal {P}}_K^G$). Since

$$\begin{aligned}\sum _{x\in {\mathcal {P}}_K\setminus {\mathcal {P}}^0} \theta (x)^2\le \sum _{x\in {\mathcal {P}}_K\setminus {\mathcal {P}}^0} S^x_1([{\bar{X}}]_x)^2\le K^{2-\varepsilon },\end{aligned}$$

we can use Proposition 8.3 to conclude. Remark that the situation here is actually much easier than for the second relation: the letters here appear on the right order (so that we could actually avoid the use of Proposition 8.3 here). $\square$

It only remains to prove Proposition 8.3 to conclude the proof of Theorem 1.

## An Inequality for Words in Random Matrices

This section is devoted to the proof of the last piece missing, Proposition 8.3.

Several times, we will need to compare the exponential of a sum in ${\mathfrak {g}}$ with the product in *G* of the exponentials.

### Lemma 9.1

There exists a constant *C* such that all positive integer *n*, for all $X_1,\dots , X_n\in {\mathfrak {g}}$,

$$\begin{aligned} d_G\Big ( \prod _{i=1}^n \exp _G(X_i), \exp _G \big (\sum _{i=1}^n X_i\big )\Big )\le C \Big (\sum _{i=1}^n \Vert X_i\Vert \Big )^2. \end{aligned}$$

### Proof

First, let is remark that, from the Dynkin’s formula and the compacity of *G*, there exists a constant *C* such for all $X,Y\in {\mathfrak {g}}$,

$$\begin{aligned} d_G(\exp _G(X)\exp _G(Y), \exp _G(X+Y))\le C \Vert X\Vert \Vert Y\Vert . \end{aligned}$$

For $j\in \{0,\dots , n\}$, let $Y_j= \sum _{i=1}^j X_i$ and $z_j=\prod _{i=n-j+1}^n \exp _G(X_i)$. We are thus trying to bound $d(z_n,\exp _G(Y_n))$. Remark that $z_{n-j}=\exp _G(X_{j+1})z_{n-j-1}$ and $Y_{j+1}=X_{j+1}+Y_{j}$. Applying the triangle inequality between the points $z_n=\exp _G(Y_1) z_{n-1}, \dots , \exp _G(Y_{n-1}) z_{1}, \exp _G(Y_n)$, we obtain

$$\begin{aligned} d(z_n,\exp _G(Y_n))&\le \sum _{j=1}^{n-1} d_G\big (\exp _G(Y_j) z_{n-j} ,\exp _G(Y_{j+1} ) z_{n-j-1} \big )\\ {}&= \sum _{j=1}^{n-1} d_G\big (\exp _G(Y_j)\exp _G(X_{j+1})z_{n-j-1} ,\exp _G(Y_{j+1} ) z_{n-j-1} \big )\\&=\sum _{j=1}^{n-1} d_G\big (\exp _G(Y_j)\exp _G(X_{j+1}),\exp _G(Y_{j}+X_{j+1} ) \big ) \\&\le \sum _{1\le i<j \le n} C \Vert X_i\Vert \Vert X_{j}\Vert \le \frac{C}{2} \big (\sum _{j=1}^{n}\Vert X_{j}\Vert \big )^2. \end{aligned}$$

$$\square $$

Under the assumptions on *g* in Proposition 8.3, for all $x\in {\mathcal {P}}$, $d_G(h_K(c^x\circ \phi ^x(g)),1)<c$, so that the logarithm of $h_K(c^x\circ \phi ^x(g))$ is well-defined, as an element of ${\mathfrak {g}}$, provided that *c* is small enough. During this section, we will call this logarithm $X_x(g)$, so that $\exp _G(X_x(g))=h_K(c^x\circ \phi ^x(g))$.

### Lemma 9.2

Let *G* be a compact Lie group, endowed with a biinvariant metric. Let $(H_x)_{x\in {\mathcal {P}}}$ be a family of ${\mathfrak {g}}$-valued random variables, each with support on the ball of radius $K^{-1}$, and assume that for all *x*,

$$\begin{aligned} {\mathbb {E}}[H_x| \sigma ((H_y)_{y<x} ) ]=0. \end{aligned}$$

Let $h_K: {\mathbb {F}}_{ {\mathcal {P}}} \rightarrow G$ be the random group morphism determined by $h_K(e_i)=\exp _G( H_i)$.

There exists $c>0$ and a constant *C* such that for any totally ordered finite set ${\mathcal {P}}$, for all $K \ge 1$ and all $g\in {\mathbb {F}}_{ {\mathcal {P}}}$ with $|g|_2\le c K$,

$$\begin{aligned} {\mathbb {E}}\Big [ \Big \Vert \sum _{x\in {\mathcal {P}}} X_x(g) \Big \Vert ^2 \Big ] \le C K^{-2} |g|_2^2. \end{aligned}$$

### Proof

Let us recall that $c^x\circ \phi ^x(g)$ is a product of $S^x_1(\pi ^{\le x}(g))$ commutators of either *x* or $x^{-1}$:

$$\begin{aligned} c^x \circ \phi ^x(g)=\prod _{k=1}^{S^x_1(\pi ^{\le x}(g)) } w_{x,k} x^{\varepsilon _{x,k}} w_{x,k},\end{aligned}$$

with $\varepsilon _{x,k}\in \{-1,1\}$ and $w_{x,k}\in {\mathbb {F}}_{{ \{y:y<x\} }}$. Set

$$\begin{aligned} {\tilde{X}}_x(g)= \sum _k {{\,\textrm{Ad}\,}}_{h_K(w_{x,k}) }( H_x^{\varepsilon _{x,k}} ).\end{aligned}$$

Lemma 9.1 ensures that

$$\begin{aligned} d_G( \exp _G(X_x(g)), \exp _G({\tilde{X}}_x(g)))\le C K^{-2}S^x_1(\pi ^{\le x}(g))^2.\end{aligned}$$

Since $X_x(g)$ and ${\tilde{X}}_x(g)$ are both smaller than $K^{-1}S^x_1(\pi ^{\le x}(g))$, hence smaller than *c*, and since the Jacobian determinant of the exponential at 0 is $1<2$, for *c* small enough,

$$\begin{aligned} \Vert X_x(g)-{\tilde{X}}_x(g)\Vert \le 2 d_G( \exp _G(X_x(g)), \exp _G({\tilde{X}}_x(g)))\le C'K^{-2}S^x_1(\pi ^{\le x}(g))^2.\end{aligned}$$

From triangle inequalities,

$$\begin{aligned} {\mathbb {E}}\Big [ \big \Vert \sum _{x} X_x(g) \big \Vert ^2 \Big ]&\le 2 {\mathbb {E}}\Big [ \big \Vert \sum _{x} {\tilde{X}}_x(g) \big \Vert ^2 \Big ]+ 2{\mathbb {E}}\Big [ \big (\sum _{x} \big \Vert X_x(g)-{\tilde{X}}_x(g) \big \Vert \big )^2 \Big ] \\&\le 2{\mathbb {E}}\Big [ \Big \Vert \sum _{x} {\tilde{X}}_x(g) \Big \Vert ^2 \Big ]+ C K^{-4} |g|_2^{4}. \end{aligned}$$

In order to control the remaining term, let us remark that

$$\begin{aligned} {\mathbb {E}}\big [{{\,\textrm{Ad}\,}}_{ h_K(w_{x,k}) }(H_x) | \sigma ( (H_y)_{y<x} ) ]= h_K(w_{x,k}) {\mathbb {E}}[H_x | \sigma ( (H_y)_{y<x} ) ]h_K(w_{x,k})^{-1}=0,\end{aligned}$$

and therefore ${\mathbb {E}}\big [{\tilde{X}}_x(g) \big |({\tilde{X}}_y(g))_{y<x}\big ]=0$. The finite sequence $(S_x= \sum _{y<x} {\tilde{X}}_y)_{x\in {\mathcal {P}}}$ is thus a martingale. Let $(S^\alpha _x)_{\alpha }$ be the components of $S_x$ in some orthonormal basis $(e_\alpha )_{\alpha }$ of ${\mathfrak {g}}$. Then, for each $\alpha$, $(S^\alpha _x)_x$ is a martingale as well, whose $i^{\hbox {th}}$ steps ${\tilde{X}}^\alpha _{x_i}$ is supported on the interval

$$\begin{aligned}{[}-K^{-1} S^{x_i}_1(\pi ^{\le x_i}(g) ), K^{-1} S^{x_i}_1(\pi ^{\le x_i}(g))].\end{aligned}$$

Thus

$$\begin{aligned} {\mathbb {E}}[ (S^\alpha _x)^2 ]= \sum _{y<x} {\mathbb {E}}[ ({\tilde{X}}^\alpha _x)^2 ] \le C K^{-2} |g|_2^2. \end{aligned}$$

We sum over $\alpha \in \{1,\dots , \dim ({\mathfrak {g}}) \}$ to conclude. $\square$

We now intend to prove Proposition 8.3. We invite the reader to look at the similarity between Lemma 9.2 that we have just proved and Proposition 8.3: they differ by the fact that the former take places in the Lie algebra whilst the latter takes place in the Lie group. Some logarithmic (in the size of ${\mathcal {P}}$) corrections also appear in the Proposition. We think the same result does hold without these corrections, but we were not able to prove it.

### Proof of Proposition 8.3

Let us first have a naive approach of the problem. We want to show that ${\mathbb {E}}[d_G(h_K(g),1)^2]$ is small. From Lemma 9.2, we already know that

17 $$\begin{aligned} {\mathbb {E}}\Big [ d_G\big (\exp _G\big (\sum _{x\in {\mathcal {P}}} X_x(g)\big ), 1\big )^2 \Big ]={\mathbb {E}}\Big [ \big \Vert \sum _{x\in {\mathcal {P}}} X_x(g)\big \Vert ^2 \Big ] \le CK^{-2}|g|^2_2. \end{aligned}$$

Besides, Lemma 9.1 gives

18 $$\begin{aligned} d_G\Big (h_K(g),\exp _G\big (\sum _{x\in {\mathcal {P}}} X_x(g) \big )\Big )\le C \Big (\sum _{x\in {\mathcal {P}}} \Vert X_x(g)\Vert \Big )^2. \end{aligned}$$

The main problem is that we would like the square to be *under* the sum, and the best we can get seems to be^7^

19 $$\begin{aligned} {\mathbb {E}}\Big [\Big (\sum _{x\in {\mathcal {P}}} \Vert X_x(g)\Vert \Big )^2\Big ]\le (\# {\mathcal {P}}) \sum _{x\in {\mathcal {P}}} {\mathbb {E}}\Big [\Vert X_x(g)\Vert ^2\Big ]. \end{aligned}$$

We will now try to reduce this factor $(\# {\mathcal {P}})$. The previous estimation is enough to conclude if $\# {\mathcal {P}}<16$, and we now assume $\# {\mathcal {P}}\ge 16$, so that $\log (\log (\# {\mathcal {P}} ))\ge 1$.

We use a *divide and conquer* algorithm. We split the alphabet ${\mathcal {P}}$ into *M* subsets of size $\#{\mathcal {P}}/M$. The choice $M\simeq \log (\#{\mathcal {P}})$ gives a nearly optimal result. Each of the subsets is then recursively split in a similar way, until each subsets contains a single element.

**Fig. 7:**
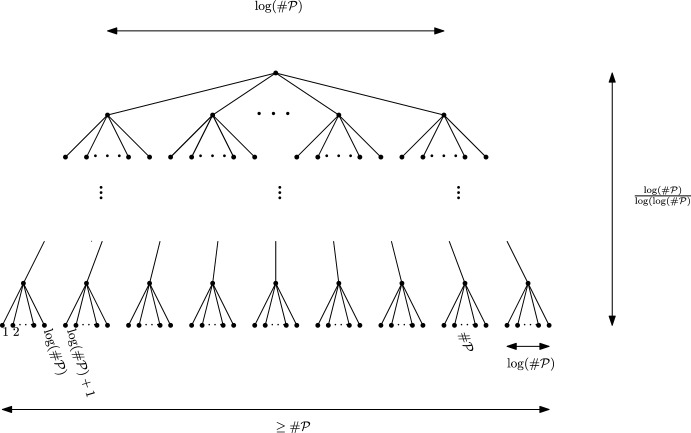
The tree $${\mathbb {T}}$$

Therefore, we consider a $\lceil \log (\#{\mathcal {P}}) \rceil$-regular rooted tree ${\mathbb {T}}$ of depth $D= \lceil \frac{ \log (\#{\mathcal {P}})}{\log (\log (\#{\mathcal {P}} ))}\rceil$, (see Fig. 7 below).

Since $\#{\mathcal {P}}=\log (\#{\mathcal {P}})^{\frac{\log (\#{\mathcal {P}})}{\log (\log (\#{\mathcal {P}}))}}\le \lceil \log (\#{\mathcal {P}}) \rceil ^D$, the tree ${\mathbb {T}}$ has more than $\#{\mathcal {P}}$ leafs. We fix some depth-first traversal of ${\mathbb {T}}$, and we enumerate the leaf accordingly. We label the $i^{\hbox {th}}$ leaf by $X_{x_i}$ if $i\le \# {\mathcal {P}}$, and by 0 otherwise. Then, we inductively label all the internal vertices, from the bottom to the root by the sum of the labels of all its children: for any internal vertex *v*, setting $\sigma (v)$ the set of the children of *v* and *l*(*v*) the set of leafs descendant from *v*,

$$\begin{aligned}X_v=\sum _{w\in \sigma (i)} X_w=\sum _{l\in l(i)} X_l.\end{aligned}$$

In particular, the root *r* is labeled by $X_r=\sum _{x\in {\mathcal {P}}} X_x$.

For $k\in \{1,\dots ,D \}$, we define ${\mathbb {T}}_k=\{ v: d_{{\mathbb {T}}}(v,r)=k-1\}$ the set of vertices at depth exactly *k*, which is naturally ordered, and $h^k= \prod _{v\in {\mathbb {T}}_k} \exp _G(X_v)$. In particular, $h^1=\exp _G\big ( \sum _{x\in {\mathcal {P}}} X_x\big )$ and $h^D= \exp _G(X_{x_1})\dots \exp _G(X_{x_{\# {\mathcal {P}}}} )$.

We already know by (17) that $h^1$ is close to 1, and our goal is to show that $h^D$ is also close to 1. It thus suffices to show that $d_G(h^i,h^{i+1})$ is small for all *i*.

Since $d_G$ is biinvariant, it satisfies the property that $d_G(ab,cd)\le d_G(a,c)+d_G(b,d)$ for any $a,b,c,d\in G$, and it follows that

$$\begin{aligned} d_G( h^i,h^{i+1})&= d_{G}\Big ( \prod _{v\in {\mathbb {T}}_i} \exp _G( \sum _{w\in \sigma (v)} X_w), \prod _{v\in {\mathbb {T}}_i} \prod _{w\in \sigma (v)} \exp _G(X_w) \Big )\\&\le \sum _{ v\in {\mathbb {T}}_i } d_G\big ( \exp _G( \sum _{w\in \sigma (v)} X_w), \prod _{w\in \sigma (v)} \exp _G(X_w) \big )\\&\le C \sum _{ v\in {\mathbb {T}}_i } \Big ( \sum _{w\in \sigma (v)} \Vert X_w\Vert \Big )^2\quad \hbox {(using Lemma}~9.1\hbox {).}\\&\le C (\#\sigma (v))\sum _{ w\in {\mathbb {T}}_{i+1} } \Vert X_w\Vert ^2. \end{aligned}$$

Therefore,

$$\begin{aligned} {\mathbb {E}}\big [ d_G( h^i,h^{i+1})\big ]&\le C \lceil \log (\# {\mathcal {P}})\rceil \sum _{ w\in {\mathbb {T}}_{i+1} } {\mathbb {E}}\big [ \Vert X_w\Vert ^2\big ] \end{aligned}$$

For $w\in {\mathbb {T}}$, let $g_w\in {\mathbb {F}}_{{\mathcal {P}}}= \prod _{i\in l(w)} c^{x_i}\circ \phi ^{x_i}(g)$, so that $h_K(g_w)=\prod _{i\in l(w)} \exp _G( X_{x_i} )$. For $i\in l(w)$, $X_{x_i}(g_w)=X_{x_i}(g)$ whilst for $i\notin l(w)$, $X_{x_i}(g_w)=0$. We also set $X_w=\sum _{i\in l(w)} X_i$. By applying Lemma 9.2 to $g_w$, we get

$$\begin{aligned} {\mathbb {E}} \big [\Vert X_w\Vert ^2 \big ]\le CK^{-2} \sum _{x\in l(w)} S^x_1(\pi ^{\le x}(g))^2,\end{aligned}$$

and therefore

$$\begin{aligned}\sum _{ w\in {\mathbb {T}}_{i+1} } {\mathbb {E}}[\Vert X_w\Vert ^2]\le CK^{-2}\sum _{ w\in {\mathbb {T}}_{i+1} } \sum _{x\in l(w)} S^x_1(\pi ^{\le x}(g))^2=CK^{-2}|g|_2^2.\end{aligned}$$

We end up with

$$\begin{aligned} {\mathbb {E}}[\;\textrm{d}_G(h^D,1) ]&\le {\mathbb {E}}[ d_G(h^1,1)^2]^{\tfrac{1}{2}}+\sum _{k=1}^{D-1} {\mathbb {E}}[d_G(h^i,h^{i+1})]\\&\le CK^{-1}|g|_2 + C D \lceil \log (\# {\mathcal {P}})\rceil K^{-2}|g|_2^2\\&\le C K^{-1}|g|_2+ C' \frac{\log (\# {\mathcal {P}})^2}{\log (\log (\# {\mathcal {P}}))}K^{-2}|g|_2^2, \end{aligned}$$

as announced. $$\square $$

## Data Availability

Data sharing not applicable to this article as no datasets were generated or analysed during the current study.

## Footnotes

1 The terminology *stable-like* is sometimes preferred, because such laws lack the nice scaling property they have in the case $G=\mathbb {R}$.

2 To be slightly more specific, we are comparing here the homotopy class of $X_{|[0,s]}\cdot [X_s,X_0]$ with the homotopy class of $X_{|[0,t]}\cdot [X_t,X_0]$.

3 Despite the title of the cited article, the given inequality *does* concern the case $p<2$.

4 Be careful with the fact that this is reversed compared to the usual notation $\alpha '\le \alpha$ for $\alpha '$ finer than $\alpha$.

5 In our case, ‘far’ is actually rather close, but not ‘extremely close’.

6 The factor 2 from which the expression here differs from the one in [27] comes from the absolute value in our definition of $D_k$.

7 Actually, $\# {\mathcal {P}}$ can easily be replaced with $\sqrt{\# {\mathcal {P}}}$ in Eq. (19). It suffices to remark that we can replace $\Big (\sum _{x\in {\mathcal {P}}} \Vert X_x(g)\Vert \Big )^2$ with $\Big (\sum _{x\in {\mathcal {P}}} \Vert X_x(g)\Vert \Big ) \max _x \sum _{y<x} \Vert X_y(g)\Vert$ in Lemma 9.2, and get a maximal inequality in Lemma 9.1.
